# Taurine-Based Hybrid Drugs as Potential Anticancer Therapeutic Agents: In Vitro, In Vivo Evaluations

**DOI:** 10.3390/ph18071056

**Published:** 2025-07-18

**Authors:** Saltanat Nakypova, Andrey Smolobochkin, Tanzilya Rizbayeva, Rakhymzhan Turmanov, Almir Gazizov, Nurgali Akylbekov, Rakhmetulla Zhapparbergenov, Roza Narmanova, Saltanat Ibadullayeva, Alena Zalaltdinova, Marat Syzdykbayev, Julia Voronina, Anna Lyubina, Alexandra Voloshina, Elena Klimanova, Tatiana Sashenkova, Denis Mishchenko, Alexander Burilov

**Affiliations:** 1 Faculty of Chemistry and Chemical Technology, Al Farabi Kazakh National University, Al-Farabi Avenue 71, Almaty 050040, Kazakhstan; nakypova.saltanat@mail.ru; 2 Arbuzov Institute of Organic and Physical Chemistry, FRC Kazan Scientific Center, Russian Academy of Sciences, Academician Arbuzov Street 8, Kazan 420088, Russia; rizbaeva.tanzilya.92@mail.ru (T.R.); agazizov@iopc.ru (A.G.); zalaltdinova.alena@iopc.ru (A.Z.); aplyubina@gmail.com (A.L.); sobaka-1968@mail.ru (A.V.); burilov@iopc.ru (A.B.); 3 Department of Chemistry, Abai Kazakh National Pedagogical University, Dostyk Avenue 13, Almaty 050010, Kazakhstan; 4 Laboratory of Engineering Profile “Physical and Chemical Methods of Analysis”, Korkyt Ata Kyzylorda University, Aiteke Bi Street 29A, Kyzylorda 120014, Kazakhstan; ulagat-91@mail.ru (R.Z.); roza_an@mail.ru (R.N.); salt_i@mail.ru (S.I.); marat.1980@mail.ru (M.S.); 5 “CNEC” LLP, Dariger Ali Lane 2, Kyzylorda 120001, Kazakhstan; 6 N.S. Kurnakov Institute of General and Inorganic Chemistry, Russian Academy of Sciences, Leninskii Avenue 31, Moscow 119991, Russia; juliavoronina@mail.ru; 7 Federal Research Center of Problems of Chemical Physics and Medicinal Chemistry, Russian Academy of Sciences, Academician Semenov Avenue 1, Chernogolovka 142432, Russia; enklimanova@mail.ru (E.K.); tsashen52@mail.ru (T.S.); mdv@icp.ac.ru (D.M.); 8 Faculty of Fundamental Physical-Chemical Engineering, M.V. Lomonosov Moscow State University, Leninskiye Gory 1, Moscow 119991, Russia; 9 Research and Educational Center in Chkrnogolovka, State University of Education, Radio Street 10a, Moscow 105005, Russia

**Keywords:** taurine, pyrrolidine, anticancer agents, aza-Michael reaction

## Abstract

**Background/Objectives**: The development of antitumor agents possessing low toxicity against non-cancerous cells is still a challenge in medicinal chemistry. In this paper, we report the antitumor activity of “hybrid structures” derived from the amino acid taurine. We have synthesized 26 compounds, structures of which were confirmed using NMR, X-ray diffractometry, and other techniques. Cytotoxicity of the obtained compounds has been evaluated using three human cancer cell lines. Pyrrolidine **4p** has exhibited the strongest antiproliferative activity against HL-60 cells with an IC_50_ of 76.7 μM, while IC_50_ against normal cells was 176.3 μM. Water-soluble derivatives of taurine have been tested for antileukemia activity in mice of the BDF1 line. Compound **4p** has been identified as the leading compound, which increases the mean survival time of mice from 40 to 100% as compared to the control group. Together, these results prove that taurine-based hybrid structures can be a promising scaffold for the discovery of potential antiproliferative agents.

## 1. Introduction

**Figure 1:**
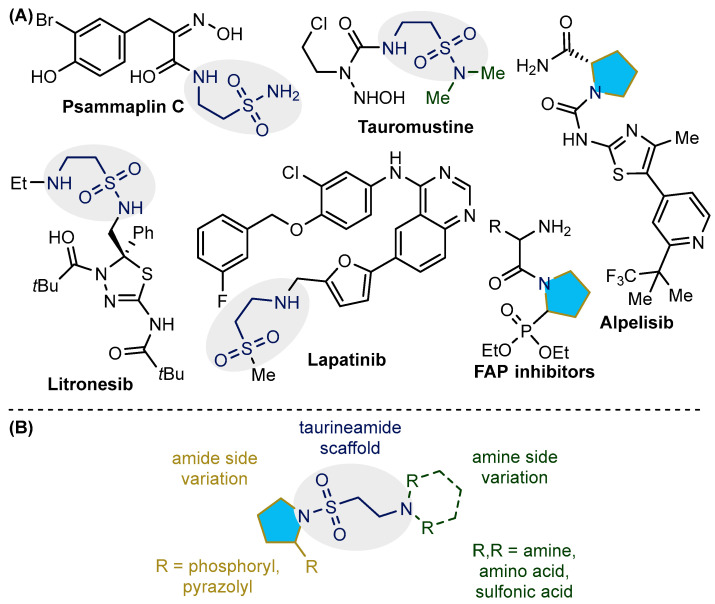
Taurine- and pyrrolidine-based biologically relevant compounds (**A**) and proposed design of taurine amide derivatives (**B**).

Taurine and its derivatives possess diverse biological activities. Taurine plays an important role in several essential biological processes such as the development of the central nervous system, calcium modulation, membrane stabilization, reproduction, and immunity [1,2,3]. Currently, there are reports positioning taurine derivatives as promising anticancer agents [4]. The recent paper on the binding of taurine with cyclin-dependent kinase CDK6 highlights taurine as a novel promising scaffold for the design of anticancer agents [5]. This is supported by the increase in the number of approved anticancer drugs with taurine moiety, as well as the ones undergoing clinical tests (Figure 1A). Examples are inhibitors of tyrosine kinase EGFR/HER-2 tauromustine [6,7,8] and lapatinib [9,10] and the inhibitor of kinesin Eg5 litronesib [11]. There is evidence that the chemotherapeutic taurolidine enhances antioxidative stress in tumor cells, which leads to apoptotic death [12]. Derivatives of benzoyltaurine amide display antitumor activity through mixed mechanisms [13]. Natural taurine amide psammaplin C is the inhibitor of carbonic anhydrase hCA XII, which is associated with cancer growth [14].

The synthesis of “hybrid structures” is a particularly promising approach to drug discovery [15]. This approach opens the way to developing improved drugs for the therapy of a number of diseases, including oncological, eliminating harsh side effects and resistance to chemotherapeutic drugs. It is quite interesting that pharmacophores in the hybrid molecular structure often preserve characteristics of initial compounds [16,17]. In addition, there are data showing that the introduction of taurine amide fragments notably improves characteristics of bioactive molecules [18].

With all of the above in mind, we designed a series of taurine amide derivatives for the development of novel compounds possessing anticancer activity (Figure 1B). For this purpose, we varied the amide fragment of taurine amide by adding a pyrrolidine ring with phosphoryl and pyrazolyl substituents at the second position. The fragment of 2-substituted pyrrolidine is included in a number of anticancer agents used in medical practice, such as dactinomycin [19], lasofoxifene [20], pacritinib [21,22], tipiracil [23,24], and alpelisib [25,26] (Figure 1A). Alpelisib is an alpha-specific phosphoinositide 3-kinase inhibitor [25]. 2-Phosphorylpyrrolidines can act as fibroblast activation protein alpha (FAPα) inhibitors [27], increased content of which is observed in 90% of carcinomas [28] and which is interesting as a target for cancer therapy [29]. Pyrazolone derivatives can inhibit the receptors of the tyrosine kinase c-mesenchymal–epithelial transition (c-Met)/vascular endothelial growth factor receptor 2 (VEGFR-2) [30] or induce apoptosis and inhibit the proliferation of cancer cells [31], which makes them promising for cancer therapy. Another possibility of modification of taurine amide is the introduction of substituents at the nitrogen atom, such as alkyl, heterocyclic, and amino acids, which could increase water solubility. Furthermore, incorporation of amino acid fragments could increase bioavailability [32,33,34].

In this paper, we describe the synthesis and investigations of anticancer activity of new taurine amide derivatives in vitro and in vivo. Some synthesized compounds displayed selective in vitro cytotoxicity against the M-HeLa cell line. In vivo studies showed that some taurine amides increase the mean survival time of tumor-bearing animals from 40 to 100%.

## 2. Results

### 2.1. Synthesis and Characterization of Taurine Amide Derivatives

The most convenient method for the synthesis of taurine amide derivatives is the aza-Michael reaction, i.e., nucleophilic addition to an activated double bond [35]. Its advantages include high yields of the product, availability of reagents, and simplicity of the experiment. We previously developed the method for the synthesis of 2-aryl-1-sulfonylpyrrolidines, which is based on the aza-Michael reaction of *N*-vinylsulfonylpyrrolidine containing the fragment of 4-chlororesorcinol at the second position with amines and hydrazines/hydrazides [36]. However, only simple amines were employed as nucleophiles previously.

**Scheme 1:**
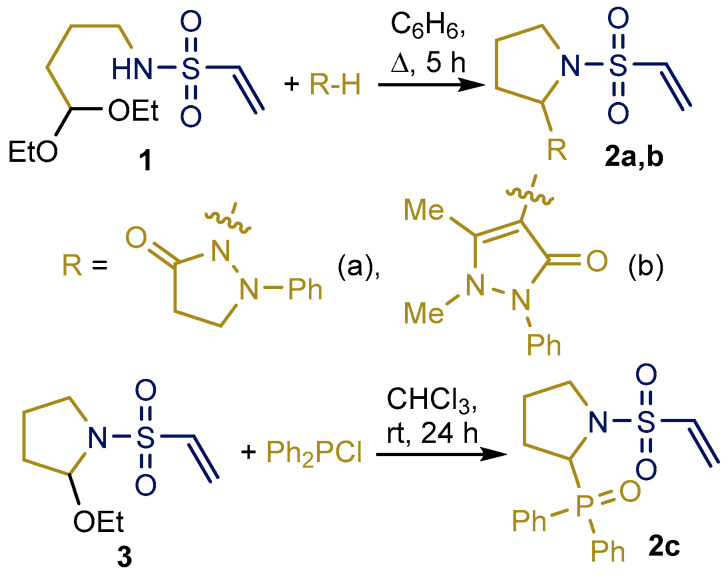
Synthesis of compounds **2a**–**c**.

To synthesize taurine derivatives, we firstly synthesized *N*-vinylsulfonylpyrrolidines **2a**–**c**. Compounds **2a,b** were prepared using a known procedure [37] via the reaction of acetal derivative **1** with 1-phenylpyrazolidine-3-one, antipyrine in refluxing benzene in the presence of trifluoroacetic acid (Scheme 1). In order to introduce phosphoryl fragments, we performed the reaction of commercially available diphenylchlorophosphine and 2-ethoxypyrrolidine **3** in chloroform at room temperature, which furnished 2-phosphorylpyrrolidine **2c** [38].

**Scheme 2:**
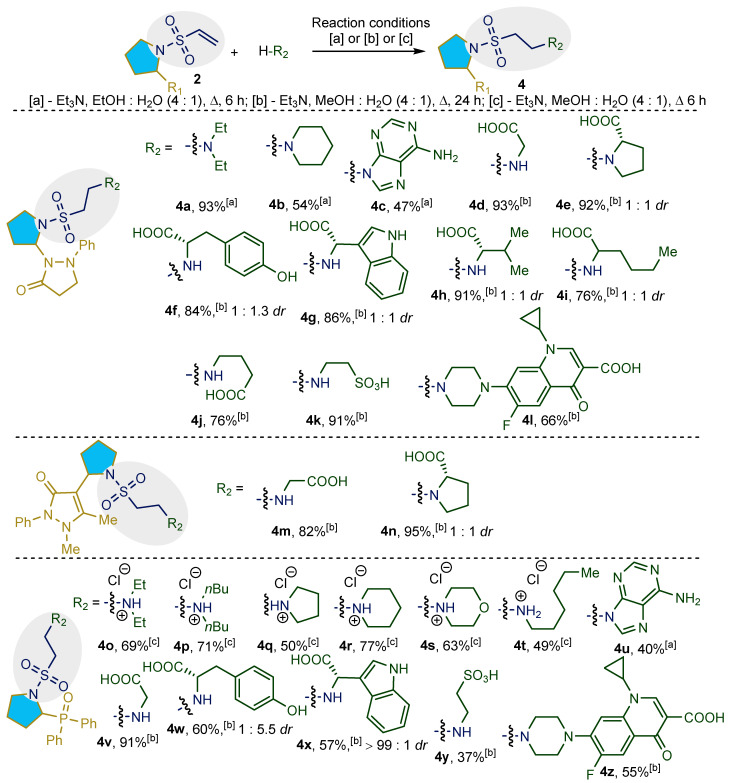
Synthesis of compounds **4a**–**z**.

We started our investigation by carrying out the reaction of pyrrolidine **2a** with diethylamine in previously reported conditions [36]. The reaction of compound **2a** with diethylamine in a refluxing ethanol–water mixture (4:1) in the presence of 10% Et_3_N afforded the product **4a** in 93% yield. Similarly, compounds **4b**,**c** bearing piperidine and adenine fragments were obtained (Scheme 2).

However, these conditions appeared to be unsuitable for amino acids. For example, the reaction of glycine with vinylpyrrolidine **2a** gave the product in 15% yield (Table 1, entry 1). Thus, additional optimization of the reaction conditions was required. An increase in the time of reaction to 24 h increased the yield of the desired product slightly (entry 2). Employment of water as a solvent did not affect the yield of the product (entry 3). We further screened a number of basic catalysts. When using DMAP or pyridine, the yield of the product increased to 35% (entry 4–6). Surprisingly, replacement of ethanol by methanol and refluxing the reaction mixture for 1 h increased the yield up to 75% (entry 7). An increase in the time of reaction to 24 h afforded the pyrrolidine derivative in 93% yield; therefore, these conditions were considered as optimal (entry 8).

Under optimized conditions, the scope of reaction was extended to various amino acids (Scheme 2). α-Amino acids (l-proline, l-tyrosine, l-tryptophan, l-valine, and d, l-norleucine) reacted with pyrrolidine **2a** smoothly to give taurine derivatives **4e**–**i**. The products were isolated as the equimolar mixture of diastereomers in 76–92% yields. It should be noted that predomination of a particular diastereomer is observed only in the case of compound **4f** (*dr* = 1:3). Employment of γ-aminobutyric acid as *N*-nucleophile also afforded 1-sulfonylpyrrolidine **4j** in 76% yield. Taurine sulfonic acid also undergoes the aza-Michael reaction smoothly to give compound **4k**. The ciprofloxacin antibiotic afforded compound **4l** in 66% yield. Interestingly, ciprofloxacin could induce apoptosis of cancerous cells [39,40,41]. Employment of *N*-vinylpyrrolidine containing an antipyrine fragment at the second position in the reactions with glycine and l-proline resulted in compounds **4m,n** in high yields (85 and 95%, respectively).

We further employed diphenyl (1-(vinylsulfonyl)pyrrolidin-2-yl)phosphine oxide **2c** as an activated alkene. The reaction of alkene **2c** with secondary amines in refluxing aqueous ethanol in the presence of Et_3_N gave the products **4o**–**s** with a high content of impurities. However, the treatment of the reaction mixture with hydrochloric acid resulted in higher than 50% isolated yields of pyrrolidines **4o**–**s**. Employment of 1-aminohexane also afforded pyrrolidine **4t**. α-Amino acids reacted smoothly with phosphine oxide **2c** to give taurine derivatives **4v**–**x**. Interestingly, compound **4w** was isolated with the predomination of one diastereomer (*dr* = 1:5.5), while in the case of pyrrolidine **4x** only one diastereomer was isolated. The pyrrolidines **4e**–**h** were isolated as equimolar mixtures of diastereomers in 92, 76, and 91% yields, respectively. However, we were able to separate the diastereomers for the compounds **4w** and **4x** (isolated *dr* = 1:5.5 and >99:1, respectively) due to the significant difference in the solubility of the diastereomers. However, this lowered the isolated yield (ca 60%). Taurine and ciprofloxacin undergo the aza-Michael reaction with compound **2c** to give the products **4y** and **4z** in 37 and 55% yields, respectively.

**Figure 2:**
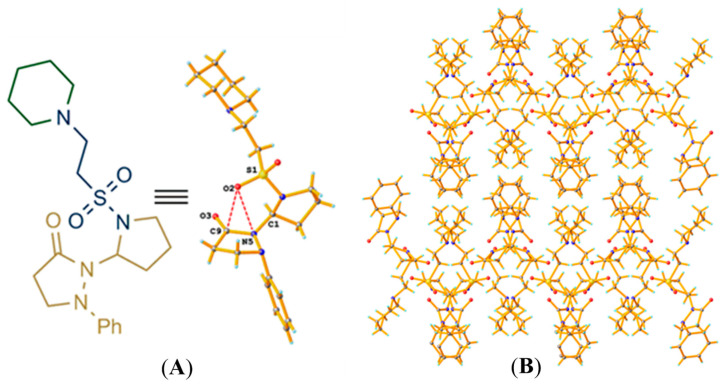
(**A**) Molecular structure and intramolecular lp…π interaction in the crystal of **4b**. Ellipsoids are given with a 50% probability. (**B**) Crystal packing of **4b**.

The molecular and crystal structure of compound **4b** was determined by X-ray analysis (Figure 2). The six-membered piperidine cycle is in the chair conformation. The five-membered cycles are rotated about 70 degrees relative to each other. This geometry is stabilized by the unusual intramolecular interaction involving an oxygen atom of the sulphone group. As was described earlier [42,43], the sulfide oxygen atoms have active lone electron pairs forming non-covalent interactions with any electron acceptor fragments. In compound **4b**, the acceptor appears to be a fragment of the delocalized pi-system O=C-N of the pyrazolidinone cycle. It is also possible to characterize this interaction as a strong lp…π [44,45], as evidenced by geometric parameters—the distance from the oxygen atom to the centroid of the pyrazolidinone cycle is 2.978(1) Å, that to the plane of the ring is –2.887(2) Å, and the angle S-O…centroid is 110.04(6)°. The molecular packing in the crystal of **4b** is formed by the CH…O interaction and represents infinite layers parallel to the plane b0c, connected into a three-dimensional structure by van der Waals interactions. Crystallographic data for structures reported in this paper have been deposited with the Cambridge Crystallographic Data Center (2169555).

### 2.2. In Vitro Studies of Anticancer Activity

With a series of taurine derivatives **4** in hand, we evaluated the cytotoxicity against normal (WI38) and tumor (M-HeLa, HuTu 80) human cell lines (Table 2). The compounds were tested against normal and tumor human cell lines at concentrations of 1–200 μM. As follows from Table 2, only a few compounds display moderate cytotoxicity against both normal and cancer cells. In this case, synthesized compounds were more active against both M-HeLa and HuTu. The most active compounds were **4l**, **4v**, **4w**, and **4y**. Cytotoxicity of compounds **4l** and **4y** against the M-HeLa cancer cell line was nearly twice as great as the cytotoxicity against normal cells (selectivity index = 1.8, selectivity index = 1.9). This is much better than the selectivity of the reference drug tamoxifen (selectivity index = 1.6). Pyrrolidine **4v** displayed selectivity that was nearly twice that of tamoxifen. On the whole, the pyrrolidines containing phosphine oxide and amino acid fragments were most active.

Due to the fact that the pyrrolidines containing a phosphine oxide fragment at position 2 of the ring displayed the highest activity, we additionally tested antileukemia cytotoxicity of compounds **4o**–**s** (Table 2). The highest activity against a cell line of promyelocyte leukemia was demonstrated by compound **4p**. The selectivity index of the HL-60 cell line compared to the leukocytes of peripheral blood of healthy donor RPMI 1788 was 2.3.

### 2.3. In Vivo Studies of Anticancer Activity

The intraperitoneal administration of the test compounds required them to be highly soluble in water, thus the choice of the test compounds **4o**–**r** was driven by this requirement. In addition, it was the phosphorus-containing derivatives of taurine that showed the best IC_50_. Moreover, compound **4p** turned out to be the most active towards leukemia cells. All these factors contributed to the choice of a series of water-soluble phosphorus-containing derivatives of taurine.

**Figure 3:**
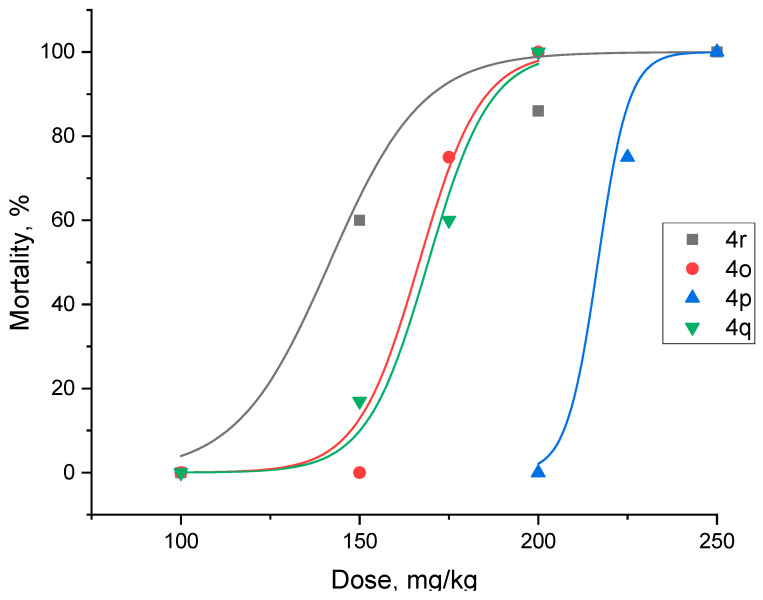
Dose–effect curves for the determination of acute toxicity parameters after single intraperitoneal addition of the solutions of compounds **4o**–**r** to hybrid BDF1 mice.

Firstly, acute in vivo toxicity was determined in order to calculate doses for the determination of the potential antileukemia activity. An acute toxicity study was carried out on the mice of the BDF1 line weighing from 20 to 24 g. In the experiment, clinically healthy animals were used, which were treated under the same conditions. To evaluate LD_50_, the toxicity gain curve was plotted against increasing doses (the Behrens method [46]) (Figure 3).

According to the results of the acute toxicity study after a single intraperitoneal addition of compounds **4o**–**r**, the half-lethal toxic doses were evaluated, which corresponded to 144, 2 ± 12, 1; 165 ± 18.2; 215 ± 16.7; and 170 ± 12.1. Thus, all test compounds are regarded as belonging to the third hazard class and represent moderately toxic compounds [47].

At the next stage, we evaluated them in vivo using syngeneic mouse leukosis P388, which plays an important role in the screening of potential antitumor agents [48]. The antitumor effect of the synthesized compounds was evaluated according to the increase in the average lifespan (ILS, %) of tumor-bearing animals. Due to the fact that physiologically relevant solvent, such as water or physiological solution [49], can be used as a solvent for parenteral administration of substances, we chose only water-soluble compounds **4o**–**r** for the in vivo study.

As follows from Table 3, pyrrolidines **4p** and **4q** exhibited antileukemia activity in the chosen dose range. It was revealed that the greatest antileukemic activity is intrinsic for compound **4p**, which increased the average lifespan of tumor-bearing animals from 40 to 100% relative to the control group. Compounds **4o** and **4r** did not display antitumor effect in the doses under study and chosen administration route.

## 3. Discussion

Overall, the present work successfully demonstrates the viability and generality of the “hybrid structures” approach towards the synthesis of taurine-based bioactive compounds. The structure of all compounds was confirmed by physico-chemical methods of analysis. Most of the compounds exhibit moderate cytotoxicity against cancer cell lines. The compound **4p** showed the best result against the leukemia cancer cell line (HL-60). The same compound exhibits antileukemic activity and extends the lifespan of the BDF1 mouse line. Presumably, the enhanced activity of compound **4p** is due to its structural features. It includes taurine, phosphine oxide, and pyrrolidine moieties, as well as two butyl substituents on the phosphorus atom.

Our results are in agreement with literature data, which confirm the viability of taurine derivatives as anticancer agents. Earlier, we reported anticancer properties of taurine-derived dibenzoxanthenes and diarylmethanes [50]. Notably, the taurine derivatives described herein are more potent compared to those reported by us earlier [50].

We emphasize that studies of anticancer properties of taurine and its derivatives are at their early stage, and no distinct mechanism of their action is yet available [51]. However, there are some publications discussing plausible mechanisms [5,52]. With further research, taurine derivatives will show great promise as a future platform for anticancer drug development.

## 4. Materials and Methods

The ^1^H and ^13^C NMR spectra were recorded on a Bruker Avance 600 spectrometer (Bruker, Billerica, MA, USA) with an operating frequency of 600 and 150 MHz, respectively, with respect to the residual proton signals of deuterated solvents (DMSO-*d*_6_, CDCl_3_). ^31^P NMR spectra were recorded on Bruker Avance II-400 spectrometer (working frequency 161.9 MHz) using 85% H_3_PO_4_ as an external standard. Crystalline samples were studied as a suspension in Vaseline oil. The melting points were determined in glass capillaries on a Stuart SMP 10 instrument (Stuart Scientific, Stone, UK). Elemental analysis of the compounds was carried out on a EuroEA3028-HT-OM high-temperature 2-reactor C, H, N-analyzer (Eurovector SpA, Pavia, Italy). ESI-TOF-MS spectra were recorded on a Bruker AmazonX instrument (Bruker). The IR spectra were recorded on a Tensor 27 spectrometer (Bruker) in the range 400–3600 cm^−1^. The crystalline products were examined as KBr discs. The halogen content was determined by the Schöniger method [53]. The X-ray diffraction data for the crystal **2a**,**b** were collected at 296 K on a Bruker SMART Apex II diffractometer (Bruker) equipped with a CCD detector (Mo-Kα, λ = 0.71073 Å, graphite monochromator). Semi-empirical absorption correction was applied by the SADABS program [54]. The structures were solved by direct methods and refined by the full-matrix least squares in the anisotropic approximation for non-hydrogen atoms. The calculations were carried out by the SHELX-2014 program package [55] using Olex2 1.2 [56]. Crystallographic data for structures reported in this paper have been deposited with the Cambridge Crystallographic Data Center (2169555). Compounds **2a**,**b** [37], **3** [38] were obtained using a previously developed method.

Reagents used: chlorodiphenylphosphine (Sigma-Aldrich, St. Louis, MO, USA, C39601), acetic acid (Sigma-Aldrich, A6283), triethylamine (Sigma-Aldrich, 471283), adenine (Tokyo Chemical Industry (TCI), Tokyo, Japan, A0149), glycine (TCI, G0099), l-proline (TCI, P0481), l-phenylalanine (TCI, P0134), l-(-)-tyrosine (TCI, T0550), l-valine (TCI, V0014), 2-aminoethanesulfonic acid (TCI, A0295), morpholine (TCI, M0465), hexylamine (TCI, H0134), l-tryptophan (TCI, T0541), ciprofloxacin (TCI, C2510). The solvents used were ethanol (EKOS-1, Moscow, Russia, Chemical Abstracts Service (CAS) number 64-17-5), diethyl ether (EKOS-1, CAS number 60-29-7), DMSO (EKOS-1, CAS number 67-68-5), chloroform (EKOS-1, CAS number 67-66-3).

### 4.1. Diphenyl(1-(vinylsulfonyl)pyrrolidin-2-yl)phosphine Oxide (***2c***)

To a solution of 2-ethoxy-1-(vinylsulfonyl)pyrrolidine **3** (0.30 g, 1.5 mmol) and chlorophosphine (0.33 g, 1.5 mmol) in dry chloroform (10 mL), acetic acid (0.1 mL, 1.75 mmol) was added. A reaction mixture was stirred at room temperature for 24 h. Then, solvent was removed on a rotary evaporator (IKA RV 3, Staufen im Breisgau, Germany) and residue thoroughly washed with diethyl ether (3 × 10 mL) and dried in vacuum to afford the target compound as a white solid. Mp: 142–144 °C; 87% yield; IR spectrum, ν, cm^−1^: 1348, 1441, 1594, 2759, 2865; ^1^H NMR (600 MHz, DMSO-*d*_6_) δ 7.93–7.88 (m, 2H), 7.84–7.79 (m, 2H), 7.60–7.50 (m, 6H), 6.75 (dd, *J* = 16.5, 9.9 Hz, 1H), 6.15–6.05 (m, 2H), 4.99–4.92 (m, 1H), 3.69–3.55 (m, 1H), 3.32–3.21 (m, 1H), 2.06–1.90 (m, 3H), 1.84–1.74 (m, 1H) ppm; ^13^C{^1^H} NMR (150 MHz, DMSO-*d*_6_) δ 133.9, 132.9 (d, *J* = 2.9 Hz), 132.5 (d, *J* = 8.9 Hz), 131.9 (d, *J* = 8.7 Hz), 129.8, 129.1 (d, *J* = 11.5 Hz), 59.1 (d, *J* = 85.0 Hz), 50.5, 27.3, 25.4 ppm; ^31^P NMR (161.9 MHz, DMSO-*d*_6_) δ 21.54 ppm; elemental analysis: calcd for C_18_H_20_NO_3_PS; C, 59.82; H, 5.58; N, 3.88; P, 8.57; S, 8.87; found C, 60.01; H, 5.75; N, 3.73; P, 8.42; S, 8.97. MS (ESI) *m*/*z* calcd for 361.4, found 362.5 [M + H]^+^, 383.8 [M + Na]^+^, 399.8 [M + K]^+^.

### 4.2. General Experimental Procedure for the Synthesis of ***4***

Method A. To a solution of compound **2** (1.38 mmol) in a 5 mL EtOH:H_2_O mixture (4:1), 0.14 mmol Et_3_N and *N*-nucleophile were added. The reaction mixture was refluxed for 6 h. The solvent was evaporated under reduced pressure, the residue was washed with diethyl ether (10 mL), and the white precipitate was recrystallized and dried under vacuum (10 torr, 10 h, 20 °C).

Method B. To a solution of compound **2** (1.38 mmol) in a 5 mL EtOH:H_2_O mixture (4:1), 0.14 mmol Et_3_N and *N*-nucleophile were added. The reaction mixture was refluxed for 24 h. The solvent was evaporated under reduced pressure, the residue was washed with diethyl ether (10 mL), and the white precipitate was recrystallized from benzene and dried under vacuum (10 torr, 10 h, 20 °C).

Method C. To a solution of compound **2** (1.38 mmol) in a 5 mL EtOH:H_2_O mixture (4:1), 0.14 mmol Et_3_N and *N*-nucleophile were added. The solvent was evaporated under reduced pressure, the residue was washed with diethyl ether (10 mL), and the white precipitate was dried under vacuum (10 torr, 10 h, 20 °C).

2-(1-((2-(Diethylamino)ethyl)sulfonyl)pyrrolidin-2-yl)-1-phenylpyrazolidin-3-one (**4a**): colorless oil; yield: 93% (method A); IR spectrum, ν, cm^−1^: 1149, 1596, 2480, 2820, 2972; ^1^H NMR (600 MHz, DMSO-*d*_6_) δ 7.31 (t, *J* = 7.9 Hz, 2H), 7.16 (d, *J* = 7.7 Hz, 2H), 7.08 (t, *J* = 7.3 Hz, 1H), 6.05–6.00 (m, 1H), 4.05–3.94 (m, 1H), 3.70–3.61 (m, 1H), 3.53–3.44 (m, 1H), 3.36–3.25 (m, 3H), 3.09–2.99 (m, 2H), 2.63 (q, *J* = 7.2 Hz, 4H), 2.58–2.49 (m, 1H), 2.45–2.33 (m, 1H), 2.07–1.95 (m, 2H), 1.89–1.79 (m, 1H), 1.76–1.66 (m, 1H), 1.08 (t, *J* = 7.2 Hz, 6H) ppm; ^13^C{^1^H} NMR (150 MHz, DMSO-*d*_6_) δ 177.1, 152.2, 129.1, 124.1, 119.3, 69.1, 57.9, 48.5, 47.4, 46.8, 46.3, 31.0, 29.3, 23.3, 11.1 ppm; elemental analysis: calculated for C_19_H_30_N_4_O_3_S; C, 57.84; H, 7.66; N, 14.20; S, 8.13; found C, 57.98; H, 7.85; N, 14.43; S, 7.86. MS (ESI) *m*/*z* calculated for 393.5, found 393.1 [M]^+^.

1-Phenyl-2-(1-((2-(piperidin-1-yl)ethyl)sulfonyl)pyrrolidin-2-yl)pyrazolidin-3-one (**4b**): Mp: 103–104 °C; yield: 54% (method A); IR spectrum, ν, cm^−1^: 1146, 1594, 2696, 2826, 2937; ^1^H NMR (600 MHz, DMSO-*d*_6_) δ 7.30 (t, *J* = 7.7 Hz, 2H), 7.18 (d, *J* = 7.9 Hz, 2H), 7.05 (t, *J* = 7.1 Hz, 1H), 6.06–5.96 (m, 1H), 3.83–3.69 (m, 2H), 3.44–3.35 (m, 1H), 3.29–3.22 (m, 2H), 3.17–3.08 (m, 1H), 2.75–2.68 (m, 1H), 2.65–2.56 (m, 1H), 2.43–2.25 (m, 6H), 2.12–2.03 (m, 1H), 1.93–1.84 (m, 1H), 1.79–1.67 (m, 2H), 1.57–1.44 (m, 4H), 1.41–1.30 (m, 2H) ppm; ^13^C{^1^H} NMR (150 MHz, DMSO-*d*_6_) δ 176.3, 152.7, 129.3, 123.7, 119.3, 69.5, 57.9, 54.2, 52.6, 48.5, 47.3, 31.2, 29.5, 25.9, 24.2, 23.6 ppm; elemental analysis: calculated for C_20_H_30_N_4_O_3_S; C, 59.09; H, 7.44; N, 13.78; S, 7.89; found C, 59.23; H, 7.61; N, 13.55; S, 7.76. MS (ESI) *m*/*z* calculated for 406.5, found 406.1 [M]^+^, 429.2 [M + Na]^+^.

2-(1-((2-(6-Amino-9*H*-purin-9-yl)ethyl)sulfonyl)pyrrolidin-2-yl)-1-phenylpyrazolidin-3-one (**4c**): Mp: 225–226 °C; yield: 47% (method A); IR spectrum, ν, cm^−1^: 1127, 1598, 1644, 1714, 2890, 3108; ^1^H NMR (600 MHz, DMSO-*d*_6_) δ 8.17 (d, *J* = 4.9 Hz, 1H), 7.30 (t, *J* = 7.6 Hz, 2H), 7.25–7.13 (m, 3H), 7.04 (t, *J* = 7.2 Hz, 1H), 6.01–5.89 (m, 1H), 4.65–4.42 (m, 2H), 3.95–3.67 (m, 4H), 3.24–3.07 (m, 2H), 2.42–2.25 (m, 2H), 2.10–1.95 (m, 1H), 1.92–1.82 (m, 1H), 1.80–1.63 (m, 2H) ppm; ^13^C{^1^H} NMR (150 MHz, DMSO-*d*_6_) δ 176.7, 156.9, 153.4, 153.0, 150.3, 141.9, 129.8, 124.4, 119.8, 119.6, 70.2, 58.4, 49.1, 48.6, 38.5, 31.6, 29.8, 24.2 ppm; elemental analysis: calculated for C_20_H_24_N_8_O_3_S; C, 52.62; H, 5.30; N, 24.55; S, 7.02; found; C, 52.76; H, 5.47; N, 24.34; S, 7.19. MS (ESI) *m*/*z* calculated for 456.5, found 457.1 [M + H]^+^, 479.5 [M + Na]^+^, 495.6 [M + K]^+^.

2-((2-((2-(5-Oxo-2-phenylpyrazolidin-1-yl)pyrrolidin-1-yl)sulfonyl)ethyl)amino)acetic acid (**4d**): Mp: 113–114 °C; yield: 93% (method B); IR spectrum, ν, cm^−1^: 1147, 1596, 1635, 1706, 2973, 3179; ^1^H NMR (600 MHz, DMSO-*d*_6_) δ 7.31 (t, *J* = 7.7 Hz, 2H), 7.18 (d, *J* = 7.6 Hz, 2H), 7.05 (t, *J* = 7.2 Hz, 1H), 5.99–5.92 (m, 1H), 3.83–3.71 (m, 2H), 3.47–3.34 (m, 3H), 3.28 (s, 2H), 3.02–2.95 (m, 2H), 2.93–2.86 (m, 1H), 2.41–2.24 (m, 2H), 2.12–2.02 (m, 1H), 1.93–1.83 (m, 1H), 1.77–1.67 (m, 2H) ppm; ^13^C{^1^H} NMR (150 MHz, DMSO-*d*_6_) δ 176.3, 172.1, 152.6, 129.3, 123.8, 119.3, 69.6, 57.9, 50.4, 46.0, 42.6, 31.1, 29.4, 23.7, 9.6 ppm; elemental analysis: calculated for C_17_H_24_N_4_O_5_S; C, 51.50; H, 6.10; N, 14.13; S, 8.09; found C, 51.77; H, 6.25; N, 14.29; S, 7.87. MS (ESI) *m*/*z* calculated for 396.4, found 397.5 [M + H]^+^.

(2*S*)-1-(2-((2-(5-Oxo-2-phenylpyrazolidin-1-yl)pyrrolidin-1-yl)sulfonyl)ethyl)pyrrolidine-2-carboxylic acid (**4e**): Mp: 97–98 °C; yield: 92% (method B); IR spectrum, ν, cm^−1^: 1147, 1631, 1706, 2887, 2980; mixture of diastereomers D_1_:D_2_ = 1:1; ^1^H NMR (600 MHz, DMSO-*d*_6_) δ 7.32 (t, *J* = 7.8 Hz, 2H), 7.19 (d, *J* = 7.8 Hz, 2H), 7.06 (t, *J* = 7.3 Hz, 1H), 5.99–5.92 (m, 1H), 3.83–3.71 (m, 2H), 3.40–3.34 (m, 1H), 3.33–3.24 (m, 3H), 3.20–3.04 (m, 4H), 3.04–2.94 (m, 2H), 2.93–2.82 (m, 1H), 2.41–2.25 (m, 2H), 2.13–1.99 (m, 2H), 1.87–1.73 (m, 4H) ppm; D_1_: ^13^C{^1^H} NMR (150 MHz, DMSO-*d*_6_) δ 176.3, 174.3, 152.5, 129.3, 123.8, 119.2, 69.6, 65.7, 61.1, 52.9, 48.0, 47.6, 45.9, 31.1, 29.3, 24.2, 23.6, 9.2 ppm; D_2_: ^13^C{^1^H} NMR (150 MHz, DMSO-*d*_6_) δ 176.2, 174.2, 152.5, 129.3, 123.8, 119.2, 69.5, 65.6, 57.9, 48.5, 47.7, 47.4, 45.6, 29.4, 29.1, 23.7, 23.4, 9.2 ppm; elemental analysis: calculated for C_20_H_28_N_4_O_5_S; C, 55.03; H, 6.47; N, 12.83; S, 7.35; found C, 55.20; H, 6.54; N, 12.69; S, 7.49. MS (ESI) *m*/*z* calculated for 436.5, found 437.1 [M + H]^+^, 459.1 [M + Na]^+^.

(2*S*)-3-(4-Hydroxyphenyl)-2-((2-((2-(5-oxo-2-phenylpyrazolidin-1-yl)pyrrolidin-1-yl)sulfonyl)ethyl)amino)propanoic acid (**4f**): Mp: 168–169 °C; yield: 84% (method B); IR spectrum, ν, cm^−1^: 1147, 1591, 1631, 1706, 2363, 2613, 2960, 3206, 3429; mixture of diastereomers D_1_:D_2_ = 1:1.3; D_1_: ^1^H NMR (600 MHz, DMSO-*d*_6_) δ 7.35–7.27 (m, 2H), 7.21–7.15 (m, 2H), 7.07–7.01 (m, 1H), 7.01–6.95 (m, 2H), 6.71–6.62 (m, 2H), 6.01–5.96 (m, 1H), 3.80–3.70 (m, 2H), 3.37–3.33 (m, 2H), 3.14–3.01 (m, 3H), 2.97–2.90 (m, 1H), 2.81–2.70 (m, 3H), 2.41–2.24 (m, 2H), 2.07–1.95 (m, 1H), 1.92–1.80 (m, 1H), 1.78–1.64 (m, 2H) ppm; D_2_: ^1^H NMR (600 MHz, DMSO-*d*_6_) δ 7.35–7.27 (m, 2H), 7.21–7.15 (m, 2H), 7.07–7.01 (m, 1H), 7.01–6.95 (m, 2H), 6.71–6.62 (m, 2H), 5.93–5.89 (m, 1H), 3.80–3.70 (m, 2H), 3.37–3.33 (m, 2H), 3.14–3.01 (m, 3H), 2.97–2.90 (m, 1H), 2.81–2.70 (m, 3H), 2.41–2.24 (m, 2H), 2.07–1.95 (m, 1H), 1.92–1.80 (m, 1H), 1.78–1.64 (m, 2H) ppm; D**_1_**: ^13^C{^1^H} NMR (150 MHz, DMSO-*d*_6_) δ 156.3, 152.6, 130.7, 130.5, 129.3, 128.2, 124.2, 121.3, 119.3, 115.6, 69.6, 63.1, 58.1, 48.7, 46.1, 42.0, 31.3, 29.5, 23.8, 9.2 ppm; D_2_: ^13^C{^1^H} NMR (150 MHz, DMSO-*d*_6_) δ (ppm) 156.1, 152.5, 130.7, 130.4, 129.3, 128.2, 123.7, 121.2, 119.2, 115.4, 69.5, 62.9, 57.9, 48.5, 46.1, 41.9, 31.1, 29.4, 23.6, 9.2 ppm; elemental analysis: calculated for C_24_H_30_N_4_O_6_S; C, 57.36; H, 6.02; N, 11.15; S, 6.38; found C, 57.50; H, 6.23; N, 11.19; S, 6.19. MS (ESI) *m*/*z* calculated for 502.5, found 503.3 [M + H]^+^, 525.4 [M + Na]^+^.

(2*S*)-2-(1*H*-Indol-3-yl)-2-((2-((2-(5-oxo-2-phenylpyrazolidin-1-yl)pyrrolidin-1-yl)sulfonyl)ethyl)amino)acetic acid (**4g**): Mp: 124–126 °C; yield: 86% (method B); IR spectrum, ν, cm^−1^: 1145, 1596, 1630, 1703, 2981, 3056; mixture of diastereomers D_1_:D_2_ = 1:1; ^1^H NMR (600 MHz, DMSO-*d*_6_) δ 7.59–7.50 (m, 1H), 7.36–7.27 (m, 3H), 7.21–7.12 (m, 3H), 7.07–7.02 (m, 2H), 6.99–6.92 (m, 1H), 6.01–5.95 (m, 1H), 3.79–3.71 (m, 2H), 3.52–3.46 (m, 2H), 3.32–3.22 (m, 3H), 3.05–2.97 (m, 2H), 2.04–1.94 (m, 1H), 1.91–1.78 (m, 1H), 1.75–1.61 (m, 2H) ppm; D_2_: ^1^H NMR (600 MHz, DMSO-*d*_6_) δ 7.59–7.50 (m, 1H), 7.36–7.27 (m, 3H), 7.21–7.12 (m, 3H), 7.07–7.02 (m, 2H), 6.99–6.92 (m, 1H), 5.94–5.88 (m, 1H), 3.79–3.71 (m, 2H), 3.52–3.46 (m, 2H), 3.32–3.22 (m, 3H), 3.05–2.97 (m, 2H), 2.04–1.94 (m, 1H), 1.91–1.78 (m, 1H), 1.75–1.61 (m, 2H) ppm; D_1_: ^13^C{^1^H} NMR (150 MHz, DMSO-*d*_6_) δ 176.3, 175.4, 155.5, 136.6, 129.3, 127.8, 124.1, 123.8, 121.4, 119.3, 118.8, 118.7, 111.7, 110.5, 69.5, 62.3, 57.9, 48.5, 42.1, 31.1, 28.9, 27.6, 23.6 ppm; D_2_: ^13^C{^1^H} NMR (150 MHz, DMSO-*d*_6_) δ (ppm) 176.2, 175.4, 155.5, 136.5, 129.3, 127.7, 124.0, 123.7, 121.2, 119.3, 118.8, 118.7, 111.7, 110.5, 69.5, 62.2, 55.1, 46.0, 41.9, 29.4, 28.8, 23.6, 10.2 ppm; elemental analysis: calculated for C_25_H_29_N_5_O_5_S; C, 58.69; H, 5.71; N, 13.69; S, 6.27; found C, 58.88; H, 5.93; N, 13.78; S, 6.16. MS (ESI) *m*/*z* calculated for 511.5, found 512.0 [M + H]^+^.

(2-((2-(5-Oxo-2-phenylpyrazolidin-1-yl)pyrrolidin-1-yl)sulfonyl)ethyl)-*L*-valine (**4h**): Mp: 183–184 °C; yield: 91% (method B); IR spectrum, ν, cm^−1^: 1148, 1584, 1620, 1708, 2364, 2626, 2880, 2968; mixture of diastereomers D_1_:D_2_ = 1:1; D_1_: ^1^H NMR (600 MHz, DMSO-*d*_6_) δ 7.36–7.27 (m, 2H), 7.22–7.15 (m, 2H), 7.08–7.02 (m, 1H), 6.02–5.97 (m, 1H), 3.83–3.74 (m, 2H), 3.20–3.10 (m, 3H), 3.02–2.91 (m, 3H), 2.79–2.70 (m, 1H), 2.37–2.27 (m, 2H), 2.09–2.01 (m, 1H), 1.93–1.84 (m, 2H), 1.78–1.68 (m, 2H), 0.95–0.86 (m, 6H) ppm; D_2_: ^1^H NMR (600 MHz, DMSO-*d*_6_) δ 7.36–7.27 (m, 2H), 7.22–7.15 (m, 2H), 7.08–7.02 (m, 1H), 5.95–5.91 (m, 1H), 3.83–3.74 (m, 2H), 3.20–3.10 (m, 3H), 3.02–2.91 (m, 3H), 2.79–2.70 (m, 1H), 2.37–2.27 (m, 2H), 2.09–2.01 (m, 1H), 1.93–1.84 (m, 2H), 1.78–1.68 (m, 2H), 0.95–0.86 (m, 6H) ppm; D_1_: ^13^C{^1^H} NMR (150 MHz, DMSO-*d*_6_) δ 176.3, 175.4, 152.6, 129.3, 123.8, 119.3, 69.6, 67.1, 59.8, 49.8, 48.6, 46.2, 42.5, 31.2, 29.4, 19.6, 18.9 ppm; D_2_: ^13^C{^1^H} NMR (150 MHz, DMSO-*d*_6_) δ (ppm) 176.2, 175.4, 152.6, 129.3, 123.8, 119.3, 69.5, 67.1, 57.9, 49.6, 48.5, 46.2, 42.5, 31.1, 23.7, 19.6, 18.8 ppm; elemental analysis: calculated for C_20_H_30_N_4_O_5_S; C, 54.78; H, 6.90; N, 12.78; S, 7.31; found C, 54.91; H, 7.12; N, 12.65; S, 7.19. MS (ESI) *m*/*z* calculated for 438.5, found 439.1 [M + H]^+^, 461.1 [M + Na]^+^.

2-((2-((2-(5-Oxo-2-phenylpyrazolidin-1-yl)pyrrolidin-1-yl)sulfonyl)ethyl)amino)hexanoic acid (**4i**): Mp: 135–136 °C; yield: 76% (method B); IR spectrum, ν, cm^−1^: 1148, 1618, 1709, 2873, 2935, 2958; mixture of diastereomers D_1_:D_2_ = 1:1; D_1_: ^1^H NMR (600 MHz, DMSO-*d*_6_) δ 7.38–7.27 (m, 2H), 7.24–7.14 (m, 2H), 7.10–7.01 (m, 1H), 6.05–5.99 (m, 1H), 3.86–3.70 (m, 2H), 3.40–3.25 (m, 3H), 3.20–2.10 (m, 2H), 3.07–2.99 (m, 1H), 2.86–2.75 (m, 1H), 2.41–2.24 (m, 2H), 2.13–2.00 (m, 1H), 1.95–1.88 (m, 1H), 1.79–1.66 (m, 2H), 1.63–1.47 (m, 2H), 1.32–1.18 (m, 4H), 0.91–0.80 (m, 3H) ppm; D_2_: ^1^H NMR (600 MHz, DMSO-*d*_6_) δ 7.38–7.27 (m, 2H), 7.24–7.14 (m, 2H), 7.10–7.01 (m, 1H), 5.97–5.92 (m, 1H), 3.86–3.70 (m, 2H), 3.40–3.25 (m, 3H), 3.20–2.10 (m, 2H), 3.07–2.99 (m, 1H), 2.86–2.75 (m, 1H), 2.41–2.24 (m, 2H), 2.13–2.00 (m, 1H), 1.95–1.88 (m, 1H), 1.79–1.66 (m, 2H), 1.63–1.47 (m, 2H), 1.32–1.18 (m, 4H), 0.91–0.80 (m, 3H) ppm; D_1_: ^13^C{^1^H} NMR (150 MHz, DMSO-*d*_6_) δ 176.3, 174.7, 152.5, 129.3, 123.8, 119.3, 69.6, 61.4, 57.9, 49.1, 48.7, 46.1, 41.7, 32.0, 31.1, 27.7, 23.6, 14.2, 9.0 ppm; D_2_: ^13^C{^1^H} NMR (150 MHz, DMSO-*d*_6_) δ (ppm) 176.3, 174.7, 152.5, 129.3, 123.8, 119.3, 69.5, 61.3, 57.9, 49.9, 48.6, 46.1, 41.6, 31.9, 29.4, 27.6, 22.5, 14.2, 9.0 ppm; elemental analysis: calculated for C_21_H_32_N_4_O_5_S; C, 55.73; H, 7.13; N, 12.38; S, 7.08; found C, 55.87; H, 7.21; N, 12.23; S, 6.87. MS (ESI) *m*/*z* calculated for 452.5, found 453.7 [M + H]^+^, 475.3 [M + Na]^+^.

4-((2-((2-(5-Oxo-2-phenylpyrazolidin-1-yl)pyrrolidin-1-yl)sulfonyl)ethyl)amino)butanoic acid (**4j**): colorless oil; yield: 76% (method B); IR spectrum, ν, cm^−1^: 1127, 1597, 1670, 2492, 2677, 2939; ^1^H NMR (600 MHz, DMSO-*d*_6_) δ 7.31 (t, *J* = 7.8 Hz, 2H), 7.18 (d, *J* = 7.9 Hz, 2H), 7.05 (t, *J* = 7.3 Hz, 1H), 5.98–5.91 (m, 1H), 3.82–3.74 (m, 2H), 3.61–3.52 (m, 2H), 3.44–3.39 (m, 2H), 3.36–2.25 (m, 3H), 3.16–3.10 (m, 1H), 3.03–2.98 (m, 1H), 2.91–2.81 (m, 1H), 2.38–2.32 (m, 2H), 2.23–2.18 (m, 1H), 2.12–2.04 (m, 1H), 1.97–1.88 (m, 2H), 1.78–1.70 (m, 2H) ppm; ^13^C{^1^H} NMR (150 MHz, DMSO-*d*_6_) δ 176.2, 174.5, 152.6, 129.3, 123.8, 119.3, 69.7, 58.0, 48.5, 46.8, 36.8, 31.2, 30.7, 29.4, 23.8, 18.0, 9.2 ppm; elemental analysis: calculated for C_19_H_28_N_4_O_5_S; C, 53.76; H, 6.65; N, 13.20; S, 7.55; found C, 53.59; H, 6.78; N, 13.20; S, 7.51. MS (ESI) *m*/*z* calculated for 424.5, found 447.7 [M + Na]^+^.

2-((2-((2-(5-Oxo-2-phenylpyrazolidin-1-yl)pyrrolidin-1-yl)sulfonyl)ethyl)amino)ethane-1-sulfonic acid (**4k**): Mp: 127–129 °C; yield: 91% (method B); IR spectrum, ν, cm^−1^: 1182, 1596, 1617, 1706, 2480, 2986, 3059; ^1^H NMR (600 MHz, DMSO-*d*_6_) δ 7.32 (t, *J* = 7.9 Hz, 2H), 7.19 (d, *J* = 7.7 Hz, 2H), 7.07 (t, *J* = 7.2 Hz, 1H), 6.01–5.92 (m, 1H), 3.85–3.72 (m, 2H), 3.60–3.50 (m, 2H), 3.28–3.21 (m, 4H), 2.84–2.78 (m, 2H), 2.77–2.70 (m, 2H), 2.42–2.29 (m, 2H), 2.17–2.18 (m, 1H), 1.93–1.86 (m, 1H), 1.80–1.70 (m, 2H) ppm; ^13^C{^1^H} NMR (150 MHz, DMSO-*d*_6_) δ 176.9, 152.1, 129.4, 124.3, 119.4, 69.5, 48.8, 48.0, 46.5, 44.5, 41.8, 36.2, 31.0, 29.3, 23.5 ppm; elemental analysis: calculated for C_17_H_26_N_4_O_6_S_2_; C, 45.73; H, 5.87; N, 12.55; S, 14.36; found C, 45.89; H, 5.94; N, 12.42; S, 14.36. MS (ESI) *m*/*z* calculated for 446.5, found 447.1 [M + H]^+^, 469.1 [M + Na]^+^.

1-Cyclopropyl-6-fluoro-4-oxo-7-(4-(2-((2-(5-oxo-2-phenylpyrazolidin-1-yl)pyrrolidin-1-yl)sulfonyl)ethyl)piperazin-1-yl)-1,4-dihydroquinoline-3-carboxylic acid (**4l**): Mp: 148–149 °C; yield: 66% (method B); IR spectrum, ν, cm^−1^: 1150, 1627, 1717, 2830, 2954; ^1^H NMR (600 MHz, DMSO-*d*_6_) δ 8.66 (s, 1H), 7.91 (d, *J* = 13.2 Hz, 1H), 7.57 (d, *J* = 7.3 Hz, 1H), 7.31 (t, *J* = 7.8 Hz, 2H), 7.19 (d, *J* = 8.0 Hz, 2H), 7.05 (t, *J* = 7.3 Hz, 1H), 6.08–6.03 (m, 1H), 3.86–3.75 (m, 3H), 3.51–3.45 (m, 1H), 3.38–3.32 (m, 6H), 3.20–3.12 (m, 1H), 2.88–2.82 (m, 1H), 2.80–2.75 (m, 1H), 2.73–2.66 (m, 4H), 2.40–2.28 (m, 2H), 2.14–2.07 (m, 1H), 1.93–1.86 (m, 1H), 1.78–1.70 (m, 2H), 1.34–1.29 (m, 2H), 1.21–1.16 (m, 2H) ppm; ^13^C{^1^H} NMR (150 MHz, DMSO-*d*_6_) δ 176.8, 176.4, 166.3, 153.4 (d, *J* = 249.1), 152.7, 148.4, 145.5 (d, *J* = 10.0), 139.6, 129.3, 123.7, 119.3, 119.1, 111.4 (d, *J* = 23.1), 107.2, 106.8, 69.6, 57.9, 52.5, 51.7, 49.8, 48.5, 47.1, 36.3, 31.3, 29.5, 23.7, 8.1 ppm; elemental analysis: calculated for C_32_H_37_FN_6_O_6_S; C, 58.88; H, 5.71; F, 2.91; N, 12.88; S, 4.91; found C, 59.03; H, 5.85; F, 3.09; N, 12.98; S, 4.76. MS (ESI) *m*/*z* calculated for 652.7, found 653.5 [M + H]^+^, 675.5 [M + Na]^+^, 691.5 [M + K]^+^.

(2-((2-(1,5-Dimethyl-3-oxo-2-phenyl-2,3-dihydro-1*H*-pyrazol-4-yl)pyrrolidin-1-yl)sulfonyl)ethyl)glycine (**4m**): Mp: 133–134 °C; yield: 82% (method B); IR spectrum, ν, cm^−1^: 1123, 1596, 1665, 2404, 2623, 2957; ^1^H NMR (600 MHz, DMSO-*d*_6_) δ 7.48 (t, *J* = 7.6 Hz, 2H), 7.34 (d, *J* = 7.4 Hz, 2H), 7.30 (t, *J* = 7.3 Hz, 1H), 4.73–4.63 (m, 1H), 3.54–3.47 (m, 2H), 3.42–3.36 (m, 2H), 3.21 (s, 2H), 3.03 (s, 3H), 2.98–2.94 (m, 2H), 2.39–2.32 (m, 1H), 2.25 (s, 3H), 2.17–2.13 (m, 1H), 2.10–2.03 (m, 1H), 1.87–1.76 (m, 1H) ppm; ^13^C{^1^H} NMR (150 MHz, DMSO-*d*_6_) δ 172.6, 165.8, 156.4, 137.1, 130.9, 128.1, 125.6, 110.0, 55.7, 51.7, 47.5, 43.9, 37.4, 33.1, 27.3, 12.6, 10.6 ppm; elemental analysis: calculated for C_19_H_26_N_4_O_5_S; C, 54.01; H, 6.20; N, 13.26; S, 7.59; found C, 54.16; H, 6.37; N, 13.38; S, 7.46. MS (ESI) *m*/*z* calculated for 422.5, found 423.1 [M + H]^+^.

(2-((2-(1,5-Dimethyl-3-oxo-2-phenyl-2,3-dihydro-1*H*-pyrazol-4-yl)pyrrolidin-1-yl)sulfonyl)ethyl)-L-proline (**4n**): colorless oil; yield: 95% (method B); IR spectrum, ν, cm^−1^: 1144, 1592, 1634, 1691, 2683, 2887, 2983; mixture of diastereomers D_1_:D_2_ = 1:1; D_1_: ^1^H NMR (600 MHz, DMSO-*d*_6_) δ 7.50–7.44 (m, 2H), 7.36–7.27 (m, 3H), 4.73–4.61 (m, 1H), 3.97–3.77 (m, 1H), 3.53–3.43 (m, 2H), 3.42–3.35 (m, 1H), 3.31–3.26 (m, 1H), 3.25–3.19 (m, 1H), 3.15–3.12 (m, 1H), 3.05 (s, 2H), 2.95–2.93 (m, 1H), 2.36–2.31 (m, 1H), 2.26 (s, 3H), 2.17–1.91 (m, 4H), 1.88–1.74 (m, 3H), 1.68–1.60 (m, 1H) ppm; D_2_: ^1^H NMR (600 MHz, DMSO-*d*_6_) δ 7.50–7.44 (m, 2H), 7.36–7.27 (m, 3H), 4.73–4.61 (m, 1H), 3.97–3.77 (m, 1H), 3.53–3.43 (m, 2H), 3.42–3.35 (m, 1H), 3.31–3.26 (m, 1H), 3.25–3.19 (m, 1H), 3.15–3.12 (m, 1H), 3.04 (s, 2H), 2.95–2.93 (m, 1H), 2.36–2.31 (m, 1H), 2.25 (s, 3H), 2.17–1.91 (m, 4H), 1.88–1.74 (m, 3H), 1.68–1.60 (m, 1H) ppm; D_1_: ^13^C{^1^H} NMR (150 MHz, DMSO-*d*_6_) δ 173.7, 165.4, 155.2, 135.9, 129.4, 126.5, 124.9, 108.3, 66.4, 60.6, 54.2, 53.1, 49.4, 48.8, 47.9, 45.9, 35.9, 29.2, 25.9, 23.4 ppm; D_2_: ^13^C{^1^H} NMR (150 MHz, DMSO-*d*_6_) δ 171.2, 164.3, 155.1, 135.6, 126.1, 124.3, 123.5, 108.1, 65.9, 56.5, 54.1, 52.9, 49.2, 48.1, 47.6, 45.6, 31.5, 29.0, 24.1, 19.0 ppm; elemental analysis: calculated for C_22_H_30_N_4_O_5_S; C, 57.13; H, 6.54; N, 12.11; S, 6.93; found C, 57.27; H, 6.76; N, 12.04; S, 7.14. MS (ESI) *m*/*z* calculated for 462.5, found 463.1 [M + H]^+^.

2-((2-(Diphenylphosphoryl)pyrrolidin-1-yl)sulfonyl)-*N*,*N*-diethylethanaminium chloride (**4o**): Mp: 127–130 °C; yield: 69% (method C); IR spectrum, ν, cm^−1^: 1348, 1441, 1598, 1670, 2756, 2863; ^1^H NMR (600 MHz, DMSO-*d*_6_) δ 7.97–7.83 (m, 4H), 7.61–7.43 (m, 6H), 5.28–5.10 (m, 1H), 3.73–3.57 (m, 2H), 3.29–3.21 (m, 2H), 3.19–3.06 (m, 4H), 2.91–2.84 (m, 2H), 2.26–2.12 (m, 1H), 2.00–1.90 (m, 2H), 1.86–1.75 (m, 1H), 1.28–1.13 (m, 6H) ppm; ^13^C{^1^H} NMR (150 MHz, DMSO-*d*_6_) δ 132.5 (d, 21.6 Hz), 132.2 (d, 29.9 Hz), 131.7 (d, 93.7 Hz), 131.7 (d, 76.7 Hz), 129.1 (d, 76.5 Hz), 129.0 (d, 77.4 Hz), 57.7 (d, 84.1 Hz), 50.2, 46.8 (d, 5.6 Hz), 41.6, 27.1, 25.2, 11.4, 8.9 ppm; ^31^P NMR (161.9 MHz, DMSO-*d*_6_) δ 31.54 ppm; elemental analysis: calculated for C_22_H_31_ClN_2_O_3_PS; C, 56.22; H, 6.65; Cl, 7.54; N, 5.96; P, 6.59; S, 6.82; found C, 56.37; H, 6.81; Cl, 7.24; N, 6.12; P, 6.64; S, 6.75. MS (ESI) *m*/*z* calculated for 469.9, found 435.2 [M − Cl]^+^.

*N*-Butyl-*N*-(2-((2-(diphenylphosphoryl)pyrrolidin-1-yl)sulfonyl)ethyl)butan-1-aminium chloride (**4p**): Mp: 118–121 °C; yield: 71% (method C); IR spectrum, ν, cm^−1^: 1344, 1445, 1598, 1670, 2756, 2861; ^1^H NMR (600 MHz, DMSO-*d*_6_) δ 7.95–7.82 (m, 4H), 7.61–7.49 (m, 6H), 5.26–5.11 (m, 1H), 3.74–3.57 (m, 3H), 3.27–3.20 (m, 1H), 3.17–3.11 (m, 1H), 3.08–3.02 (m, 1H), 2.99–2.89 (m, 2H), 2.86–2.79 (m, 2H), 2.24–2.09 (m, 1H), 1.98–1.89 (m, 2H), 1.85–1.75 (m, 1H), 1.65–1.56 (m, 4H), 1.33–1.27 (m, 4H), 0.95–0.83 (m, 6H) ppm; ^13^C{^1^H} NMR (150 MHz, DMSO-*d*_6_) δ 132.5 (d, 25.5 Hz), 132.2 (d, 21.6 Hz), 131.7 (d, 84.5 Hz), 131.6 (d, 84.2 Hz), 129.1 (d, 76.7 Hz), 128.9 (d, 77.5 Hz), 57.6 (d, 84.1 Hz), 52.3, 50.2, 46.9, 44.4, 27.8, 27.1, 25.2, 19.8, 13.9 ppm; ^31^P NMR (161.9 MHz, DMSO-*d*_6_) δ 31.58 ppm; elemental analysis: calculated for C_26_H_40_ClN_2_O_3_PS; C, 59.24; H, 7.65; Cl, 6.73; N, 5.31; P, 5.88; S, 6.08; found C, 59.32; H, 7.78; Cl, 6.56; N, 5.31; P, 5.65; S, 5.85. MS (ESI) *m*/*z* calculated for 527.1, found 491.3 [M − Cl]^+^.

1-(2-((2-(Diphenylphosphoryl)pyrrolidin-1-yl)sulfonyl)ethyl)pyrrolidin-1-ium chloride (**4q**): Mp: 96–99 °C; yield: 50% (method C); IR spectrum, ν, cm^−1^: 1347, 1441, 1596, 1672, 2755, 2853; ^1^H NMR (600 MHz, DMSO-*d*_6_) δ 7.99–7.82 (m, 4H), 7.64–7.46 (m, 6H), 5.23–5.08 (m, 1H), 3.69–3.57 (m, 1H), 3.29–3.16 (m, 1H), 3.08–2.93 (m, 2H), 2.56–2.50 (m, 2H), 2.42–2.30 (m, 4H), 2.21–2.06 (m, 1H), 2.03–1.89 (m, 2H), 1.87–1.75 (m, 1H), 1.71–1.58 (m, 4H) ppm; ^13^C{^1^H} NMR (150 MHz, DMSO-*d*_6_) δ 132.6 (d, 21.6 Hz), 132.2 (d, 13.1 Hz), 131.7 (d, 60.0 Hz), 131.6 (d, 59.4 Hz), 129.1 (d, 54.8 Hz), 128.9 (d, 55.1 Hz), 57.8 (d, 83.5 Hz), 53.4, 50.1, 47.9, 45.6 (d, 151.6 Hz), 26.9, 25.2, 23.1 ppm; ^31^P NMR (161.9 MHz, DMSO-*d*_6_) δ 30.34 ppm; elemental analysis: calculated for C_22_H_30_ClN_2_O_3_PS; C, 56.34; H, 6.45; Cl, 7.56; N, 5.97; P, 6.60; S, 6.84; found C, 56.21; H, 6.37; Cl, 7.42; N, 6.16; P, 6.62; S, 6.65. MS (ESI) *m*/*z* calculated for 468.9, found 433.0 [M − Cl]^+^.

1-(2-((2-(Diphenylphosphoryl)pyrrolidin-1-yl)sulfonyl)ethyl)piperidin-1-ium chloride (**4r**): Mp: 131–134 °C; yield: 77% (method C); IR spectrum, ν, cm^−1^: 1348, 1445, 1597, 1671, 2742, 2862; ^1^H NMR (600 MHz, DMSO-*d*_6_) δ 8.03–7.78 (m, 4H), 7.62–7.47 (m, 6H), 5.23–5.13 (m, 1H), 3.76–3.66 (m, 2H), 3.63–3.55 (m, 1H), 3.41–3.31 (m, 2H), 3.29–3.23 (m, 1H), 3.17–3.11 (m, 1H), 3.09–3.03 (m, 1H), 2.87–2.82 (m, 1H), 2.31–2.12 (m, 1H), 2.01–1.91 (m, 2H), 1.89–1.83 (m, 1H), 1.81–1.72 (m, 4H), 1.58–1.49 (m, 2H), 1.42–1.28 (m, 1H) ppm; ^13^C{^1^H} NMR (150 MHz, DMSO-*d*_6_) δ 132.5 (d, 17.6 Hz), 132.2 (d, 19.5 Hz), 131.7 (d, 89.1 Hz), 131.7 (d, 89.3 Hz), 129.1 (d, 82.0 Hz), 128.9 (d, 81.5 Hz), 57.9 (d, 84.1 Hz), 52.6 (d, 30.5 Hz), 50.1 (d, 35.6 Hz), 44.3, 43.9, 27.0, 25.2, 22.5, 21.6 ppm; ^31^P NMR (161.9 MHz, DMSO-*d*_6_) δ 32.79 ppm; elemental analysis: calculated for C_23_H_32_ClN_2_O_3_PS; C, 57.19; H, 6.68; Cl, 7.34; N, 5.80; P, 6.41; S, 6.64; found C, 57.35; H, 6.89; Cl, 7.21; N, 5.94; P, 6.40; S, 6.75. MS (ESI) *m*/*z* calculated for 483.0, found 447.2 [M − Cl]^+^.

4-(2-((2-(Diphenylphosphoryl)pyrrolidin-1-yl)sulfonyl)ethyl)morpholin-4-ium chloride (**4s**): Mp: 69–72 °C; yield: 63% (method C); IR spectrum, ν, cm^−1^: 1335, 1442, 1596, 1670, 2753, 2867; ^1^H NMR (600 MHz, DMSO-*d*_6_) δ 7.95–7.79 (m, 4H), 7.62–7.46 (m, 6H), 5.21–5.08 (m, 1H), 3.85–3.77 (m, 4H), 3.73–3.66 (m, 2H), 3.61–3.54 (m, 1H), 3.40–3.34 (m, 1H), 3.28–3.23 (m, 1H), 3.22–3.15 (m, 1H), 3.09–3.02 (m, 4H), 2.25–2.12 (m, 1H), 2.02–1.92 (m, 2H), 1.89–1.79 (m, 1H) ppm; ^13^C{^1^H} NMR (150 MHz, DMSO-*d*_6_) δ 132.4 (d, 25.8 Hz), 131.7 (d, 85.4 Hz), 131.7 (d, 86.1 Hz), 129.1 (d, 80.2 Hz), 129.0 (d, 80.5 Hz), 63.6, 58.1 (d, 83.9 Hz), 51.5, 50.0 (d, 48.9 Hz), 44.1, 43.1, 27.0, 25.1 ppm; ^31^P NMR (161.9 MHz, DMSO-*d*_6_) δ 31.61 ppm; elemental analysis: calculated for C_22_H_30_ClN_2_O_4_PS; C, 54.48; H, 6.23; Cl, 7.31; N, 5.78; P, 6.39; S, 6.61; found C, 54.59; H, 6.37; Cl, 7.18; N, 5.94; P, 6.33; S, 6.51. MS (ESI) *m*/*z* calculated for 484.9, found 449.2 [M − Cl]^+^.

*N*-(2-((2-(Diphenylphosphoryl)pyrrolidin-1-yl)sulfonyl)ethyl)hexan-1-aminium chloride (**4t**): colorless oil; yield: 49% (method C); IR spectrum, ν, cm^−1^: 1348, 1441, 1598, 1671, 2778, 2886; ^1^H NMR (600 MHz, DMSO-*d*_6_) δ 8.07–7.79 (m, 4H), 7.72–7.48 (m, 6H), 5.35–5.12 (m, 1H), 3.63–3.37 (m, 6H), 3.27–3.18 (m, 2H), 2.93–2.80 (m, 2H), 2.73–2.59 (m, 2H), 2.22–2.11 (m, 1H), 2.02–1.87 (m, 2H), 1.85–1.72 (m, 1H), 1.63–1.42 (m, 2H), 1.37–1.16 (m, 2H), 0.85 (t, *J* = 7.3 Hz, 3H) ppm; ^13^C{^1^H} NMR (150 MHz, DMSO-*d*_6_) δ 132.3 (d, 27.9 Hz), 131.7 (d, 84.3 Hz), 129.5 (d, 71.0 Hz), 128.9 (d, 80.1 Hz), 128.1 (d, 11.5 Hz), 53.6 (d, 84.3 Hz), 50.2, 47.5, 46.7, 41.8, 29.0, 27.1, 26.8, 25.4, 25.2, 19.9, 14.0 ppm; ^31^P NMR (161.9 MHz, DMSO-*d*_6_) δ 31.81 ppm. Elemental analysis: calculated for C_24_H_36_ClN_2_O_3_PS; C, 57.76; H, 7.27; Cl, 7.10; N, 5.61; P, 6.21; S, 6.43; found C, 57.97; H, 7.43; Cl, 7.33; N, 5.45; P, 6.09; S, 6.59. MS (ESI) *m*/*z* calculated for 499.1, found 486.4 [M − Cl + Na]^+^.

(1-((2-(6-Amino-9*H*-purin-9-yl)ethyl)sulfonyl)pyrrolidin-2-yl)diphenylphosphine oxide (**4u**): Mp: 228–229 °C; yield: 40% (method A); IR spectrum, ν, cm^−1^: 1308, 1419, 1603, 1668, 2689, 2799, 2983; ^1^H NMR (600 MHz, DMSO-*d*_6_) δ 12.83 (br s, 2H), 8.11 (s, 1H), 7.93–7.77 (m, 4H), 7.63–7.38 (m, 6H), 7.23 (s, 1H), 5.18–4.98 (m, 1H), 4.42–4.26 (m, 2H), 3.64–3.56 (m, 1H), 3.51–3.42 (m, 2H), 3.27–3.21 (m, 1H), 2.21–2.09 (m, 1H), 2.00–1.90 (m, 2H), 1.86–1.76 (m, 1H) ppm; ^13^C{^1^H} NMR (150 MHz, DMSO-*d*_6_) δ 156.4, 152.8, 149.8, 141.2, 139.7, 132.5 (d, 18.1 Hz), 132.1 (d, 21.9 Hz), 131.7 (d, 83.5 Hz), 131.6 (d, 84.0 Hz), 129.0 (d, 93.1 Hz), 128.9 (d, 93.4 Hz), 119.1, 58.1 (d, 84.3 Hz), 50.1, 49.1, 37.8, 26.9, 25.1 ppm; ^31^P NMR (161.9 MHz, DMSO-*d*_6_) δ 30.58 ppm; elemental analysis: calculated for C_23_H_25_N_6_O_3_PS; C, 55.64; H, 5.08; N, 16.93; P, 6.24; S, 6.46; found C, 55.72; H, 5.13; N, 17.14; P, 6.14; S, 6.31. MS (ESI) *m*/*z* calculated for 496.1, found 496.9 [M + H]^+^, 519.2 [M + Na]^+^, 535.2 [M + K]^+^.

2-((2-((2-(Diphenylphosphoryl)pyrrolidin-1-yl)sulfonyl)ethyl)amino)acetic acid (**4v**): Mp: 157–159 °C; yield: 91% (method A); IR spectrum, ν, cm^−1^: 1334, 1524, 1616, 2612, 2948; ^1^H NMR (600 MHz, DMSO-*d*_6_) δ 7.99–7.77 (m, 4H), 7.64–7.43 (m, 6H), 5.22–5.04 (m, 1H), 3.65–3.55 (m, 2H), 3.32–3.11 (m, 4H), 2.87–2.66 (m, 2H), 2.22–2.07 (m, 1H), 2.02–1.91 (m, 2H), 1.87–1.73 (m, 1H) ppm; ^13^C{^1^H} NMR (150 MHz, DMSO-*d*_6_) δ 171.9, 132.3 (d, 25.6 Hz), 131.7 (d, 87.7 Hz), 131.6 (d, 87.3 Hz), 129.0 (d, 88.7 Hz), 128.9 (d, 89.3 Hz), 57.8 (d, 84.9 Hz), 50.1 (d, 37.4 Hz), 46.1, 26.8, 25.3, 9.2 ppm; ^31^P NMR (161.9 MHz, DMSO-*d*_6_) δ 30.58 ppm; elemental analysis: calculated for C_20_H_25_N_2_O_5_PS; C, 55.04; H, 5.77; N, 6.42; P, 7.10; S, 7.35; found C, 54.87; H, 5.89; N, 6.60; P, 7.00; S, 7.17. MS (ESI) *m*/*z* calculated for 436.4, found 437.1 [M + H]^+^, 459.1 [M + Na]^+^.

(2-((2-(Diphenylphosphoryl)pyrrolidin-1-yl)sulfonyl)ethyl)-l-tyrosine (**4w**): Mp: 216–217 °C yield: 60%; (method B); mixture of diastereomers D_1_:D_2_ = 1:5.5; IR spectrum, ν, cm^−1^: 1330, 1515, 1590, 1613, 2606, 2836, 2946; D_1_: ^1^H NMR (600 MHz, DMSO-*d*_6_) δ ^1^H NMR (600 MHz, DMSO-*d*_6_) δ 7.92–7.78 (m, 4H), 7.58–7.45 (m, 6H), 7.04 (d, 8.8 Hz), 6.65 (d, 8.2 Hz), 5.12–5.05 (m, 1H), 3.87–3.80 (m, 1H), 3.58–3.53 (m, 2H), 2.99–2.89 (m, 2H), 2.74–2.69 (m, 1H), 2.62–2.55 (m, 1H), 2.19–2.05 (m, 1H), 1.99–1.88 (m, 2H), 1.85–1.74 (m, 1H), 1.36–1.28 (m, 1H), 1.21–1.16 (m, 1H) ppm; D_2_: ^1^H NMR (600 MHz, DMSO-*d*_6_) δ 7.92–7.78 (m, 4H), 7.58–7.45 (m, 6H), 6.98 (d, 8.3 Hz), 6.65 (d, 8.2 Hz), 5.12–5.05 (m, 1H), 3.87–3.80 (m, 1H), 3.58–3.53 (m, 2H), 2.99–2.89 (m, 2H), 2.74–2.69 (m, 1H), 2.62–2.55 (m, 1H), 2.19–2.05 (m, 1H), 1.99–1.88 (m, 2H), 1.85–1.74 (m, 1H), 1.36–1.28 (m, 1H), 1.21–1.16 (m, 1H) ppm; ^31^P NMR (161.9 MHz, DMSO-*d*_6_) δ 30.44 ppm. D_1_: ^13^C{^1^H} NMR (150 MHz, DMSO-*d*_6_) δ 176.8, 166.3, 132.3 (d, 28.2 Hz), 131.7 (d, 90.3 Hz), 131.6 (d, 89.9 Hz), 130.5, 128.9 (d, 93.4 Hz), 128.9 (d, 93.9 Hz), 128.9, 115.6, 62.7, 57.9 (d, 85.1 Hz), 50.0, 44.4, 38.1, 26.8, 25.3, 8.1 ppm; D_2_: ^13^C{^1^H} NMR (150 MHz, DMSO-*d*_6_) δ (ppm) 175.1, 156.3, 132.3 (d, 28.2 Hz), 131.7 (d, 90.3 Hz), 131.6 (d, 89.9 Hz), 130.5, 128.9 (d, 93.4 Hz), 128.9 (d, 93.9 Hz), 128.9, 115.4, 62.8, 57.9 (d, 85.1 Hz), 50.3, 46.1, 36.3, 26.8, 25.3, 9.6 ppm; ^31^P NMR (161.9 MHz, DMSO-*d*_6_) δ 30.44 ppm; elemental analysis: calculated for C_27_H_31_N_2_O_6_PS; C, 59.77; H, 5.76; N, 5.16; P, 5.71; S, 5.91; found C, 59.61; H, 5.59; N, 4.95; P, 5.86; S, 6.06. MS (ESI) *m*/*z* calculated for 542.5, found 543.5 [M + H]^+^, 565.5 [M + Na]^+^.

(2*S*)-2-((2-((2-(Diphenylphosphoryl)pyrrolidin-1-yl)sulfonyl)ethyl)amino)-2-(1*H*-indol-3-yl)acetic acid (**4x**): Mp: 179–180 °C; yield: 57% (method B); >99:1 *dr*; IR spectrum, ν, cm^−1^: 1321, 1437, 1614, 2951, 3055; ^1^H NMR (600 MHz, DMSO-*d*_6_) δ 7.95–7.74 (m, 4H), 7.62–7.43 (m, 7H), 7.33 (d, *J* = 8.4 Hz), 7.13 (d, *J* = 2.4 Hz), 7.06 (t, *J* = 7.7 Hz), 6.98 (t, *J* = 7.2 Hz), 5.17–5.06 (m, 1H), 3.56–3.50 (m, 2H), 3.05–2.92 (m, 3H), 2.82–2.72 (m, 1H), 2.67–2.59 (m, 1H), 2.19–2.05 (m, 1H), 2.00–1.88 (m, 2H), 1.84–1.71 (m, 1H) ppm; ^13^C{^1^H} NMR (150 MHz, DMSO-*d*_6_) δ 175.2, 136.5, 132.3 (d, 28.3 Hz), 131.7 (d, 90.3 Hz), 131.6 (d, 89.5 Hz), 128.9 (d, 94.2 Hz), 128.9 (d, 94.7 Hz), 127.8, 124.1, 121.3, 118.8, 118.7, 111.7, 110.3, 62.8, 57, 8 (d, 85.1 Hz), 50.1 (d, 59.7 Hz), 41.8, 28.7, 26.8, 25.3 ppm; ^31^P NMR (161.9 MHz, DMSO-*d*_6_) δ 30.45 ppm; elemental analysis: calculated for C_28_H_30_N_3_O_5_PS; C, 60.97; H, 5.48; N, 7.62; P, 5.62; S, 5.81; found C, 61.14; H, 5.63; N, 7.54; P, 5.79; S, 6.06. MS (ESI) *m*/*z* calculated for 551.6, found 552.4 [M + H]^+^.

2-((2-((2-(Diphenylphosphoryl)pyrrolidin-1-yl)sulfonyl)ethyl)amino)ethane-1-sulfonic acid (**4y**): Mp: 131–132 °C; yield: 37% (method B); IR spectrum, ν, cm^−1^: 1319, 1438, 1524, 1618, 2929, 3054; ^1^H NMR (600 MHz, DMSO-*d*_6_) δ 8.30 (s, 1H), 7.95–7.79 (m, 4H), 7.64–7.46 (m, 7H), 5.19–5.09 (m, 1H), 3.61–3.55 (m, 1H), 3.48–3.44 (m, 1H), 3.23–3.17 (m, 2H), 3.15–3.02 (m, 4H), 2.83-–2.80 (m, 1H), 2.76–2.70 (m, 1H), 2.24–2.12 (m, 1H), 2.02–1.89 (m, 2H), 1.87–1.77 (m, 1H) ppm; ^13^C{^1^H} NMR (150 MHz, DMSO-*d*_6_) δ 132.5 (d, 17.1 Hz), 131.7 (d, 68.9 Hz), 131.7 (d, 68.8 Hz), 129.1 (d, 73.3 Hz), 129.1 (d, 74.3 Hz), 57.9 (d, 84.1 Hz), 50.1, 46.3, 41.9, 36.6, 27.1, 25.3 ppm; ^31^P NMR (161.9 MHz, DMSO-*d*_6_) δ 30.88 ppm; elemental analysis: calculated for C_20_H_27_N_2_O_6_PS_2_; C, 49.37; H, 5.59; N, 5.76; P, 6.37; S, 13.18; found C, 49.21; H, 5.68; N, 5.76; P, 6.27; S, 13.18. MS (ESI) *m*/*z* calculated for 486.5, found 487.3 [M + H]^+^, 509.3 [M + Na]^+^.

1-Cyclopropyl-7-(4-(2-((2-(diphenylphosphoryl)pyrrolidin-1-yl)sulfonyl)ethyl)piperazin-1-yl)-6-fluoro-4-oxo-1,4-dihydroquinoline-3-carboxylic acid (**4z**): Mp: 120–121 °C; yield: 55% (method B); IR spectrum, ν, cm^−1^: 1335, 1469, 1628, 1726, 2831, 2948, 3056; ^1^H NMR (600 MHz, DMSO-*d*_6_) δ 8.63 (s, 1H), 7.95–7.81 (m, 5H), 7.60–7.49 (m, 7H), 5.25–5.11 (m, 1H), 3.73–3.63 (m, 1H), 3.55–3.21 (m, 10H), 3.13–2.99 (m, 2H), 2.83–2.80 (m, 1H), 2.76–2.73 (m, 1H), 2.58–2.56 (m, 1H), 2.24–2.09 (m, 1H), 2.05–1.92 (m, 2H), 1.88–1.76 (m, 1H), 1.36–1.31 (m, 1H), 1.23–1.14 (m, 2H), 1.11–1.04 (m, 1H) ppm; ^13^C{^1^H} NMR (150 MHz, DMSO-*d*_6_) δ 176.7, 166.3, 153.4 (d, 249.6 Hz), 148.3, 145.5 (d, 9.9 Hz), 139.5, 132.3 (d, 14.8 Hz), 131.7 (d, 67.2 Hz), 131.6 (d, 68.8 Hz), 129.0 (d, 53.6 Hz), 128.9 (d, 54.1 Hz), 57.7 (d, 85.1 Hz), 52.3, 51.4, 50.1, 48.0, 45.9, 36.2, 26.8, 25.4, 8.0 ppm; ^31^P NMR (161.9 MHz, DMSO-*d*_6_) δ 30.39 ppm; elemental analysis: calculated for C_35_H_38_FN_4_O_6_PS; C, 60.68; H, 5.53; F, 2.74; N, 8.09; P, 4.47; S, 4.63; found C, 60.56; H, 5.67; F, 2.74; N, 8.09; P, 4.64; S, 4.79. MS (ESI) *m*/*z* calculated for 692.7, found 693.7 [M + H]^+^, 715.7 [M + Na]^+^.

### 4.3. Cytotoxicity Assay

The toxic effect on cells was determined using the colorimetric method of cell proliferation with thiazolyl blue tetrazolium bromide (MTT) (Sigma-Aldrich). For this, 10 μL of MTT reagent in Hanks’ balanced salt solution (HBSS) (final concentration 0.5 mg/mL) was added to each well. The plates were incubated at 37 °C for 2–3 h in an atmosphere humidified with 5% CO_2_. Absorbance was recorded at 540 nm using an Invitrologic microplate reader (Medical Biological Union, Novosibirsk, Russia). Experiments for all compounds were repeated three times. IC_50_ was calculated using an online microscopy and analysis system—“Quest Graph™ IC_50_ Calculator” AAT Bioquest, Inc., Pleasanton, CA, USA, https://www.aatbio.com/tools/ic50-calculator (accessed on 11 May 2022). 4′,6-Diamidino-2-phenylindole (DAPI) and propidium iodide were purchased from Sigma-Aldrich. The M-HeLa clone 11 human epithelioid cervical carcinoma cell line, strain of HeLa, clone of M-HeLa; human duodenal cancer cell line (HuTu 80) from the Type Culture Collection of the Institute of Cytology (Russian Academy of Sciences, Moscow, Russia); and Chang liver cell line (Human liver cells) from N.F. Gamaleya Research Center of Epidemiology and Microbiology (Moscow, Russia) were used in the experiments. For the experiments we used tumor cell cultures of HL-60: human, peripheral blood, promyelocytic leukemia; RPMI 1788: human, healthy donor peripheral blood leukocytes from the Type Culture Collection of the Institute of Cytology (Russian Academy of Sciences).

The cells were cultured in a standard Eagle’s nutrient medium manufactured at the Chumakov Institute of Poliomyelitis and Virus Encephalitis (PanEco, Moscow, Russia) and supplemented with 10% fetal calf serum (Thermo Fisher Scientific, Waltham, MA, USA) and 1% non-essential amino acids (Thermo Fisher Scientific). The cells were plated into a 96-well plate (Nunc, Roskilde, Denmark) at a concentration of 1 × 10^5^ cells/mL, 150 μL of medium per well, and cultured in a CO_2_ incubator at 37 °C. Twenty-four hours after seeding the cells into wells, the compound under study was added at a preset dilution, 150 μL in each well. The dilutions of the compounds were prepared immediately in nutrient media; 5% DMSO that does not induce inhibition of cells at this concentration was added for better solubility. The experiments were repeated three times. Intact cells cultured in parallel with experimental cells were used as a control [56].

### 4.4. Evaluation of In Vivo Acute Toxicity

Acute toxicity was evaluated using mice of the BDF1 line weighing from 20 to 24 g. In the experiment, clinically healthy animals treated under the same conditions were used. A total of 4–6 animals were used for each dose. All experiments on animals were carried out according to the Nursery and Vivarium, Russian Academy of Sciences Federal Research Center of Problems of Chemical Physics and Medicinal Chemistry (FRC PCPMC RAS) and in accordance with the rules established by the Commission on Bioethics of the FRC PCPMC RAS. One day after administration of the specimen, the animals were continuously treated for 4 h. Then, death of animals and clinical pattern of intoxication were evaluated once a day for 14 d. The test compounds were added intraperitoneally once. The individual volume of each animal was calculated from the mass of the body and dose of the test compound.

To determine LD_50_ (the lethal dose for 50% of animals), the toxicity gain curve was plotted against the increasing dose (Behrens method). The lethal dose (LD_100_) for all animals and the maximum tolerable dose (MTD) values were also evaluated. Results of the studies are given in the form of tables and graphs.

### 4.5. Antitumor Activity

As model tumors for the determination of antitumor activity of phosphorus-containing derivatives of pyrrolidine, the strain of lymphatic leukemia P388 was chosen. Lymphatic leukemia P388 was transplanted into hybrid mice of the BDF1 line intraperitoneally in accordance with the standard procedure. The inoculum contained 10^6^ tumor cells in isotonic solution of NaCl [57]. Initial compounds were also added intraperitoneally in the form of aqueous solution at the doses from 18 to 83 mg/kg after 1, 5, and 9 d of transplantation of experimental tumor. In each experiment, the control group was represented by the cohort of animals with tumors not treated with the compounds. Each group consisted of six mice. The animals were inspected daily over at least 60 days. The effectiveness of therapy was evaluated by the number of surviving animals, as well as by the increase in the average lifespan (ILS%), which was evaluated as the percentage of the mean survival time (MST) of the treated group (t) as compared to the control group (c): ILS (%) = (ALS t/ALS c) × 100.

### 4.6. Statistical Treatment Methods

Statistical treatment of the results of the study was performed according to the guidelines [49]. To carry out statistical analysis, the GraphPad Prism 6.0 program (GraphPad Software, San Diego, CA, USA) was used. The data are provided in the form of M ± SD, where M is the mean value and SD is the root-mean-square deviation. The Student *t*-criterion was used as a parametric criterion. Statistically significant differences were determined at the confidence interval *p* < 0.05.

## 5. Conclusions

In summary, in this work, we synthesized for the first time a large library of novel “hybrid” derivatives of taurine. The suggested method could incorporate various substituents to target compounds both at nitrogen and sulfur atoms. In this case, the compounds possessing a particular biological activity can be devised. The cytotoxicity of the compounds against normal and cancer cell lines has been investigated. Compounds **4l**, **4o**, **4p**, **4y** display a moderate cytotoxicity against the M-HeLa cancer cell line. In this case, the best result was demonstrated by the pyrrolidines containing a phosphine oxide fragment at position 2 of the heterocycle. Pyrrolidine **4p** displayed IC_50_ = 76.7 µM against HL-60 leukemia cells, with a selectivity index of 2.3, which makes it a leading compound. The compound **4p** possesses the greatest antileukemic activity because its employment increased the mean survival time of tumor-bearing mice from 40 to 100%. Thus, further studies are necessary for the evaluation of the anticancer potential of “hybrid” taurine derivatives.

## Figures and Tables

**Table 1:** Optimization of the catalytic reaction conditions ^[a]^.

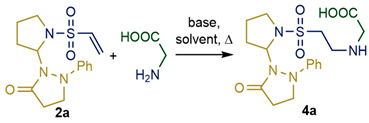				
Entry	Solvent	Base	Time [h]	Yield [%]
1	EtOH:H_2_O (4:1)	Et_3_N	6	15 ^[b]^
2	EtOH:H_2_O (4:1)	Et_3_N	24	20 ^[b]^
3	H_2_O	Et_3_N	24	10 ^[b]^
4	EtOH:H_2_O (4:1)	Pyridine	24	34 ^[b]^
5	EtOH:H_2_O (4:1)	DMAP	1	24 ^[b]^
6	EtOH:H_2_O (4:1)	DMAP	24	35 ^[b]^
7	MeOH:H_2_O (4:1)	Et_3_N	6	75 ^[b]^
8	MeOH:H_2_O (4:1)	Et_3_N	24	93 ^[c]^

^[a]^ The reaction was performed with 1.38 mmol of **2a**, 1.38 mmol glycine, and 0.14 mmol of base in 5 mL of solvent at reflux. ^[b]^ Yield according to NMR data. ^[c]^ Isolated yields.

**Table 2:** Cytotoxic effects of compounds **4** on the cancer and normal human cell lines ^[a]^.

Test Compounds	Cancer Cell Lines						Normal Cell Lines	
	M-HeLa ^[b]^		HuTu 80 ^[c]^		HL-60 ^[d]^		WI38 ^[e]^	RPMI 1788 ^[f]^
	IC_50_ (μM)	SI ^[g]^	IC_50_ (μM)	SI ^[g]^	IC_50_ (μM)	SI ^[g]^	IC_50_ (μM)	IC_50_ (μM)
**4** **b**	87 ± 6.8	1	>100	-	-	-	88.3 ± 7.1	-
**4c**	>100	-	>100	-	-	-	100	-
**4l**	85.4 ± 7.5	1.8	>100	-	-	-	154 ± 12	-
**4m**	>100	1	87.0 ± 7.0	>1.1	-	-	>100	-
**4o**	56.7 ± 4.8	1.5	>100	-	>100	-	84.3 ± 7.2	>100
**4p**	57.0 ± 4.6	1.1	94.4 ± 8.7	-	76.7 ± 6.1	2.3	63.2 ± 5.7	176.3 ± 3.4
**4** **q**	>100	-	>100	-	>100	-	>100	>100
**4** **r**	96.0 ± 8.6	>1	97.3 ± 8.2	>1	>100	-	>100	>100
**4s**	78.4 ± 6.6	1.2	>100	-	>100	-	91.8 ± 8.4	>100
**4u**	>100	-	>100	-	-	-	>100	-
**4v**	96.2 ± 8.4	2.2	>100	-	-	-	211 ± 17	-
**4x**	98.2 ± 8.3	1.3	>100	-	-	-	128 ± 10	-
**4y**	62.5 ± 5.3	1.9	100 ± 9.2	1.2	-	-	120 ± 9.3	-
**4z**	85.9 ± 8.2	1.4	64.6 ± 5.1	1.9	-	-	123 ± 9.6	-
Tamoxifen	28.0 ± 2.5	1.6	-	-	-	-	46.2 ± 3.5	-
5-Fluorouracil	75.6 ± 6.1	-	65.2 ± 5.5	1	-	-	62.0 ± 4.8	-
Doxorubicin	-	-	-	-	3.0 ± 4.7	1.1	-	3.3 ± 6.6

^[a]^ Three independent experiments were carried out; the activity was tested at concentrations of 1–200 µM; (-)—no selectivity. ^[b]^ M-HeLa—human cervix carcinoma; ^[c]^ HuTu 80—human duodenal adenocarcinoma; ^[d]^ HL-60—human leukemia; ^[e]^ WI38—human cell line composed of fibroblasts; ^[f]^ RPMI 1788—human lymphocyte cell line; ^[g]^ SI—selectivity index.

**Table 3:** Mean survival time and increased lifespan (ILS) of murine leukemia P388 upon individual treatment with **4o**–**r**.

Dose	4o		Dose	4p		Dose	4q		Dose	4r	
	MST ± SD ^[a]^ (Days)	ILS ^[b]^ (%)		MST ± SD (Days)	ILS (%)		MST ± SD (Days)	ILS (%)		MST ± SD (Days)	ILS (%)
0			0				0		0		
12	10.7 ± 1.0	7	16	20 ± 3.1	100	12	8.3 ± 0.6	−9	10	9.8 ± 0.2	−9
17	9.5 ± 0.8	−5	22	18 ± 3.2	80	17	10.3 ± 0.9	13	15	10.5 ± 0.8	−2
26	11.7 ± 1.9	17	34	17.5 ± 2.7	75	27	12.2 ± 1.7	33	22	11.2 ± 0.8	4
37	10.7 ± 0.9	7	48	15.5 ± 3.4	55	38	15.5 ± 3.9	69	31	10.8 ± 0.7	0
55	10.5 ± 1.2	5	72	14.2 ± 2.4	42	57	10.2 ± 0.2	11	47	10.7 ± 0.8	−1

^[a]^ MST, mean survival time; SD, standard deviation. ^[b]^ ILS%, the percentage of the median survival time (MST) of the treated group (t) to that of the control group (c). ILS% = ((MSTt/MSTc) − 1) × 100.

## Data Availability

The data presented in this study are contained within the article and Supplementary Materials. Further inquiries can be directed to the corresponding authors.

## Supplementary Materials

The following supporting information can be downloaded at: https://www.mdpi.com/article/10.3390/ph18071056/s1, Crystal data for **4b**—Table S1: H-bonds in crystals of investigated compound; Figure S1: (A) Molecular structure and intramolecular lp…π interaction in crystal of **4b**. Ellipsoids are given with a 50% probability. (B) crystal packing of **4b**. ^1^H, ^13^C and ^31^P NMR spectrum of compounds **2c**, **4a**–**z**—Figures S2–S73.
