# Supplementary material for: Taurine-Based Hybrid Drugs as Potential Anticancer Therapeutic Agents: In Vitro, In Vivo Evaluations
Source: Pharmaceuticals (Basel). 2025 Jul 18;18(7):1056. doi: 10.3390/ph18071056 (PMC12300679; doi:10.3390/ph18071056)
Supplement: Supplementary file 1 [file pharmaceuticals-18-01056-s001.zip › pharmaceuticals-3708041-supplementary.pdf]

Supporting Information

Taurine-Based Hybrid Drugs as Potential Anticancer Thera-peutic Agents: *In Vitro*, *In Vivo* Evaluations

Saltanat Nakypova, Andrey Smolobochkin, Tanzilya Rizbayeva, Rakhymzhan Turmanov, Almir Gazizov, Nurgali Akylbekov, Rakhmetulla Zhapparbergenov, Roza Narmanova, Saltanat Ibadullayeva, Alena Zalaltdinova, Marat Syzdykbayev, Julia Voronina, Anna Lyubina, Alexandra Voloshina, Elena Klimanova, Tatiana Sashenkova, Denis Mishchenko and Alexander Burilov

Table of Contents Page

Crystal data for **4b** 2

^{1}H, ^{13}C and ^{31}P NMR spectrum of compounds **2c**, **4a-z** 3

X-ray studies

Crystal data for X: $C_{20}H_{30}N_4O_3S$, M = 406.54, colorless crystal, monoclinic, space group $P2_1/c$, Z = 4, a= 14.2432 (11), b=10.1035 (7), c=14.2923 (10)Å, β=100.230 (3)˚, V = 2024.1(3)Å^3, ρ_{calc} = 1.334 g/cm^3, μ= 0.19 mm^{-1}, 18,858 reflections collected (*h*, *k*, *l*), 3986 independent (*R*int 0.036) and 3,463 observed reflections [$I > 2\sigma(I)$], 253 refined parameters, R = 0.0382, wR^2 = 0.0905, max. residual electron density is 0.37 (-0.37) eÅ^{-3}.

Table S1. H-bonds in crystals of investigated compound.

H-bond	D–H, Å	H...A, Å	D...A, Å	D-H...A, ˚
C1-H1...O3	0.98	2.53	2.897(2)	102
C1-H1...O1	0.98	2.36	3.291(2)	159
C2-H2B...O2	0.97	2.51	3.191(2)	127
C10-H10B...O1	0.97	2.56	3.291(2)	132
C16-H16B...O3	0.97	2.60	3.485(2)	152
C23-H23...N5	0.93	2.44	2.759(2)	100

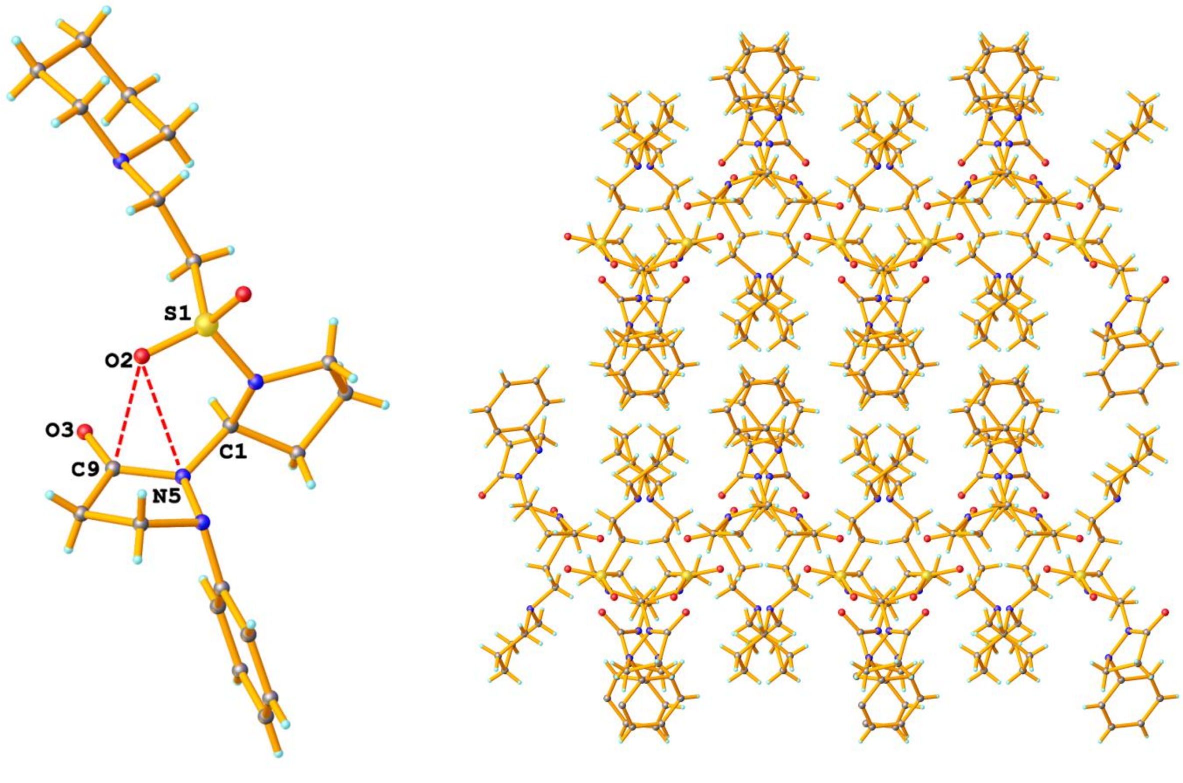

A B

Figure S1. A) Molecular structure and intramolecular lp…π interaction in crystal of **4b**. Ellipsoids are given with a 50% probability. B) crystal packing of **4b**.

Copies of NMR spectra

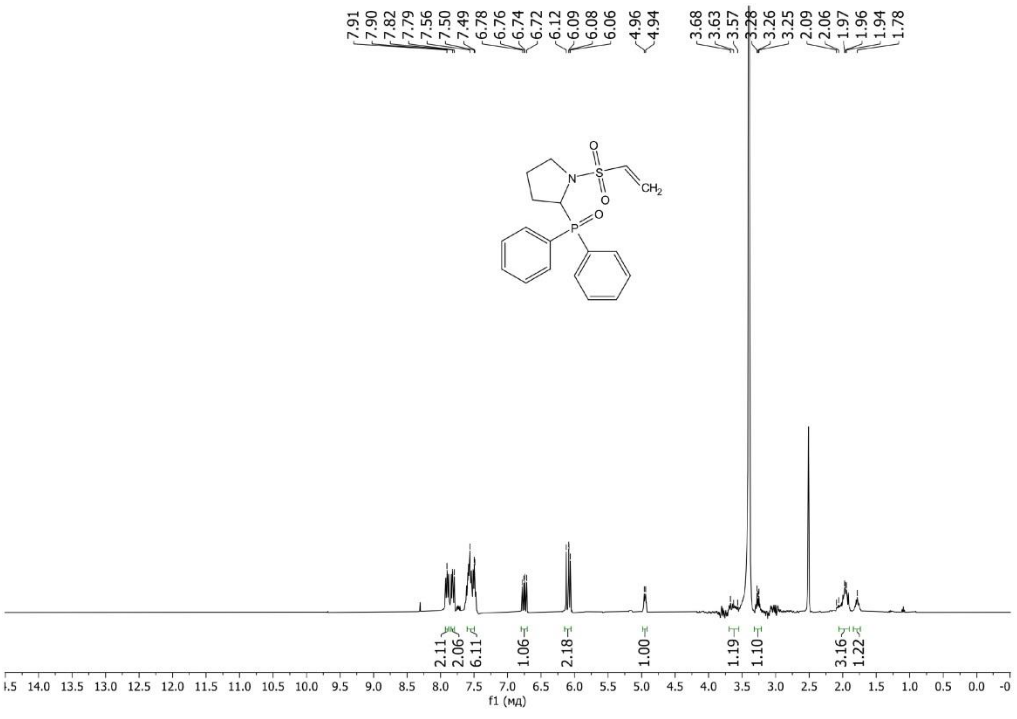

Figure S2. ^{1}H NMR (DMSO-d_6, 600 MHz) spectrum of the compound **2c**.

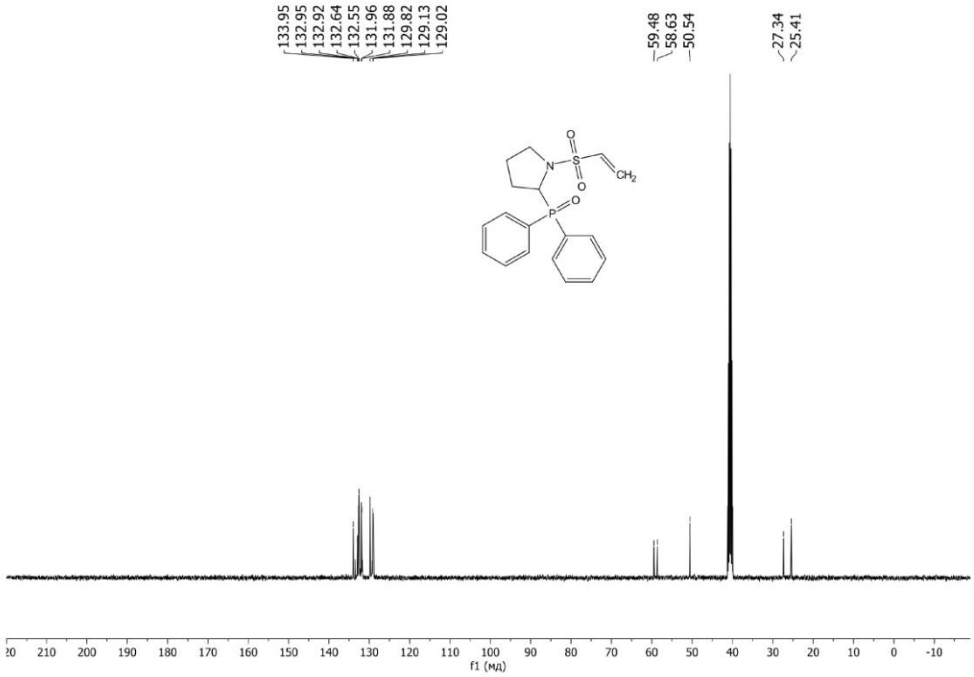

Figure S3. ^{13}C{^{1}H} NMR (DMSO-d_6, 150 MHz) spectrum of the compound **2c**.

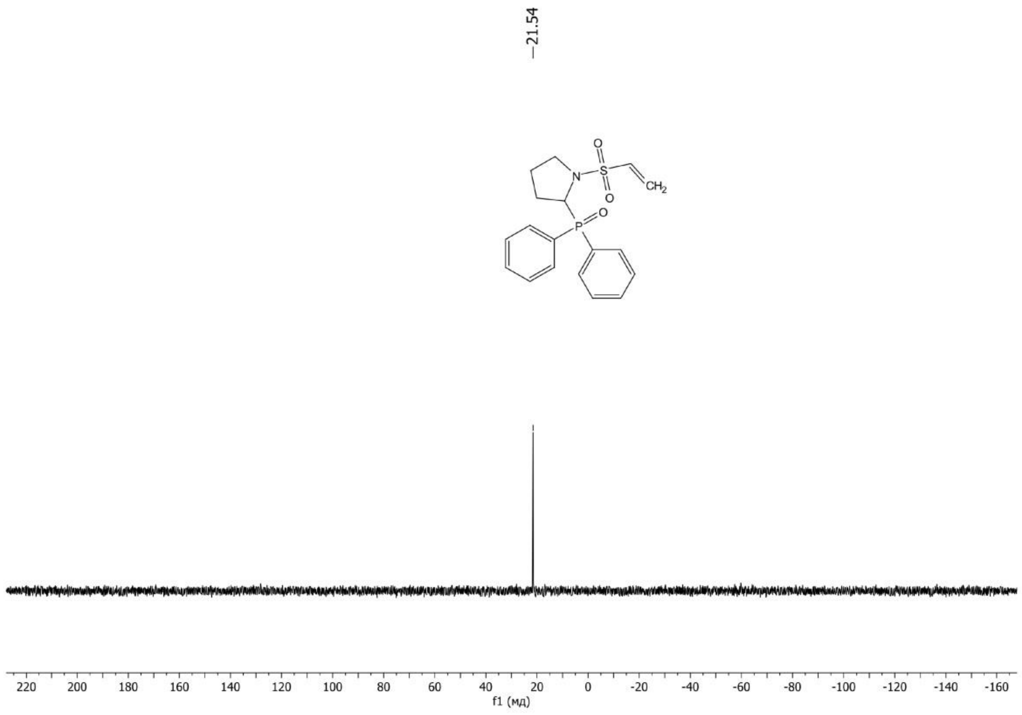

Figure S4. $^{31}P\{^{1}H\}$ NMR spectrum (DMSO-d_6, 161.9 MHz) of the compound **2c**.

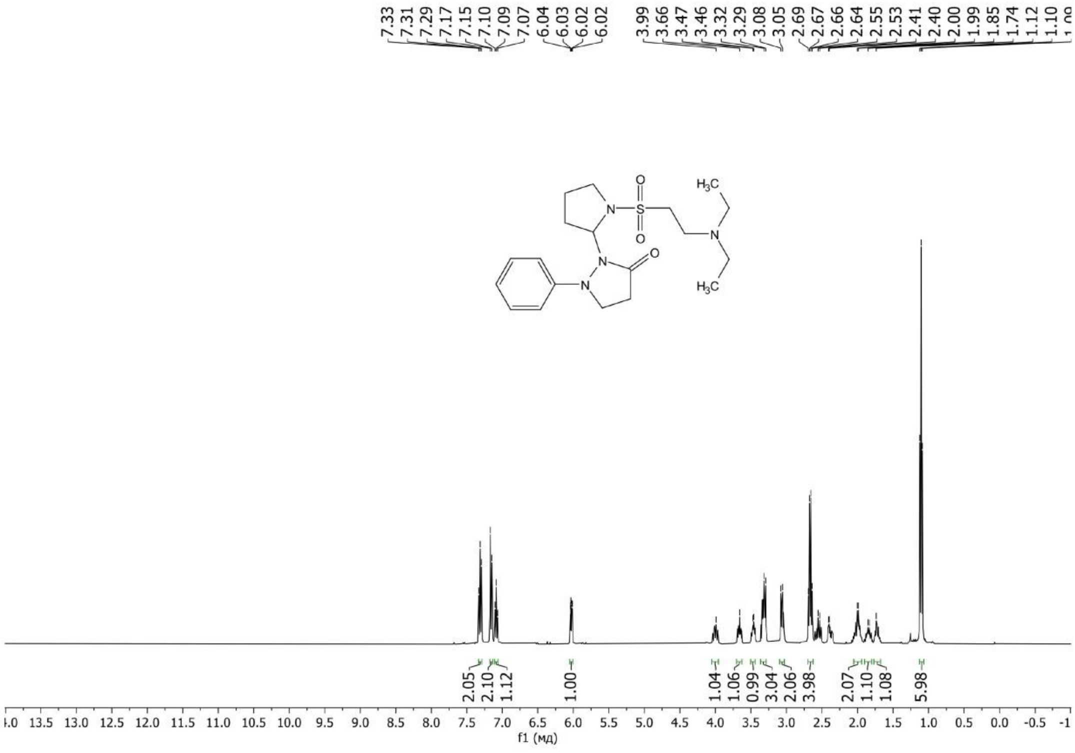

Figure S5. ^{1}H NMR (DMSO-d_6, 600 MHz) spectrum of the compound **4a**.

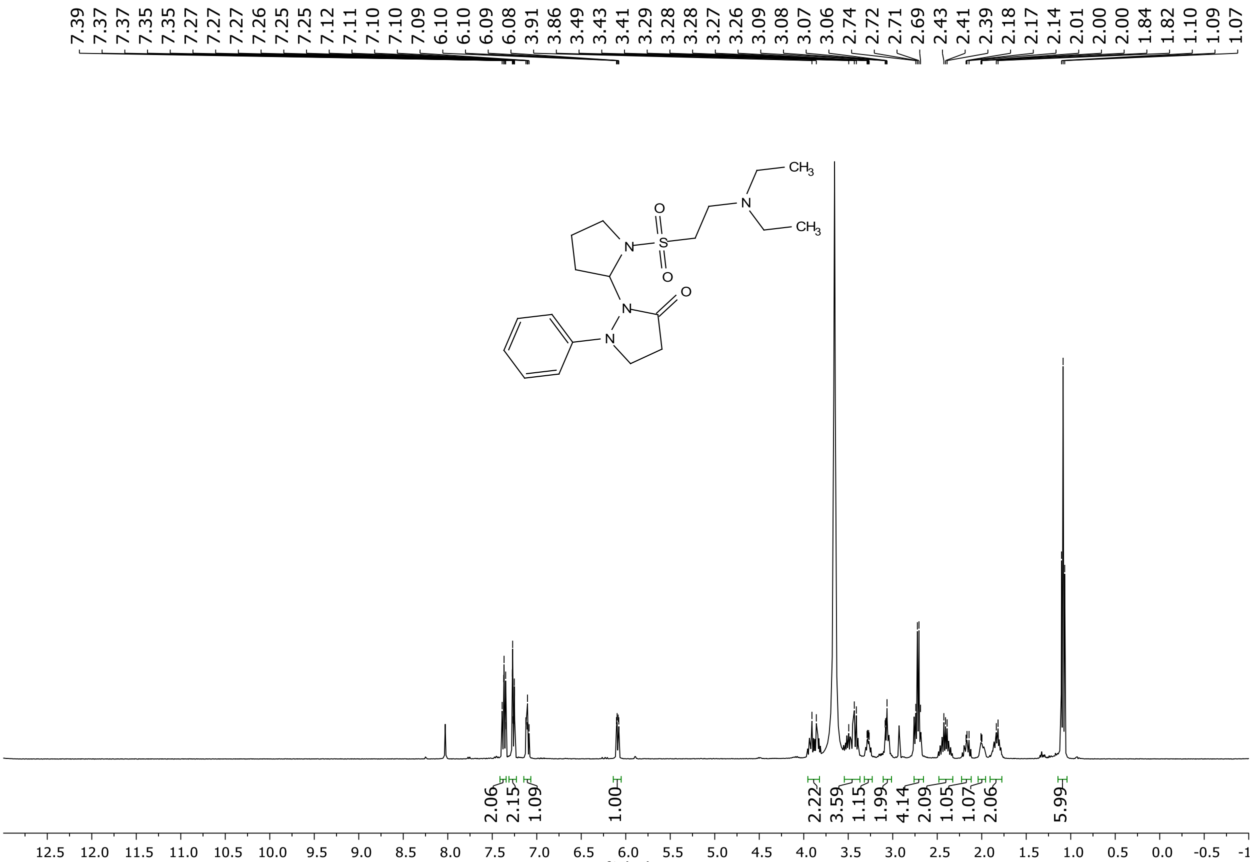

Figure S6. ^{1}H NMR (DMF-d_7, 600 MHz) spectrum of the compound **4a**.

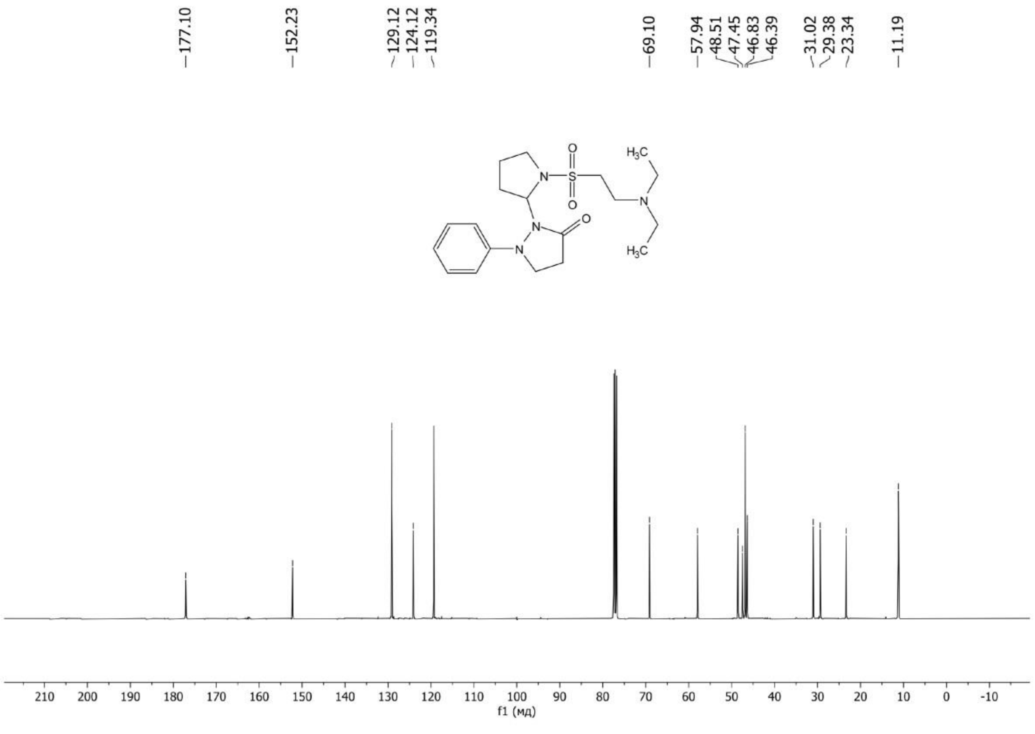

Figure S7. ^{13}C{^{1}H} NMR (DMSO-d_6, 150 MHz) spectrum of the compound **4a**.

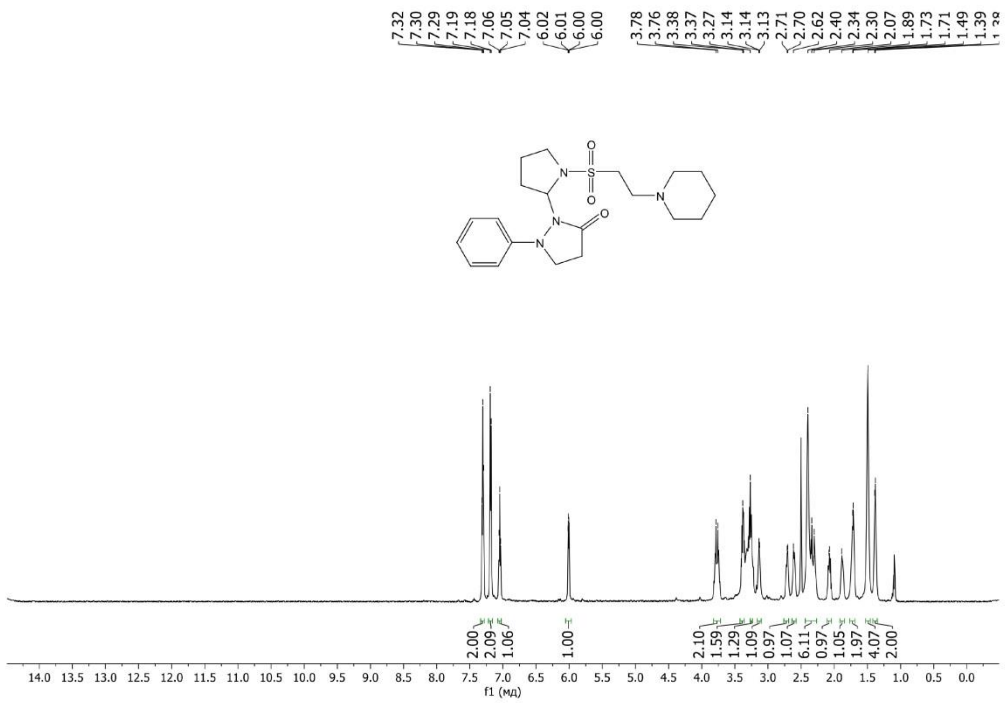

Figure S8. ^{1}H NMR (DMSO-d_6, 600 MHz) spectrum of the compound **4b**.

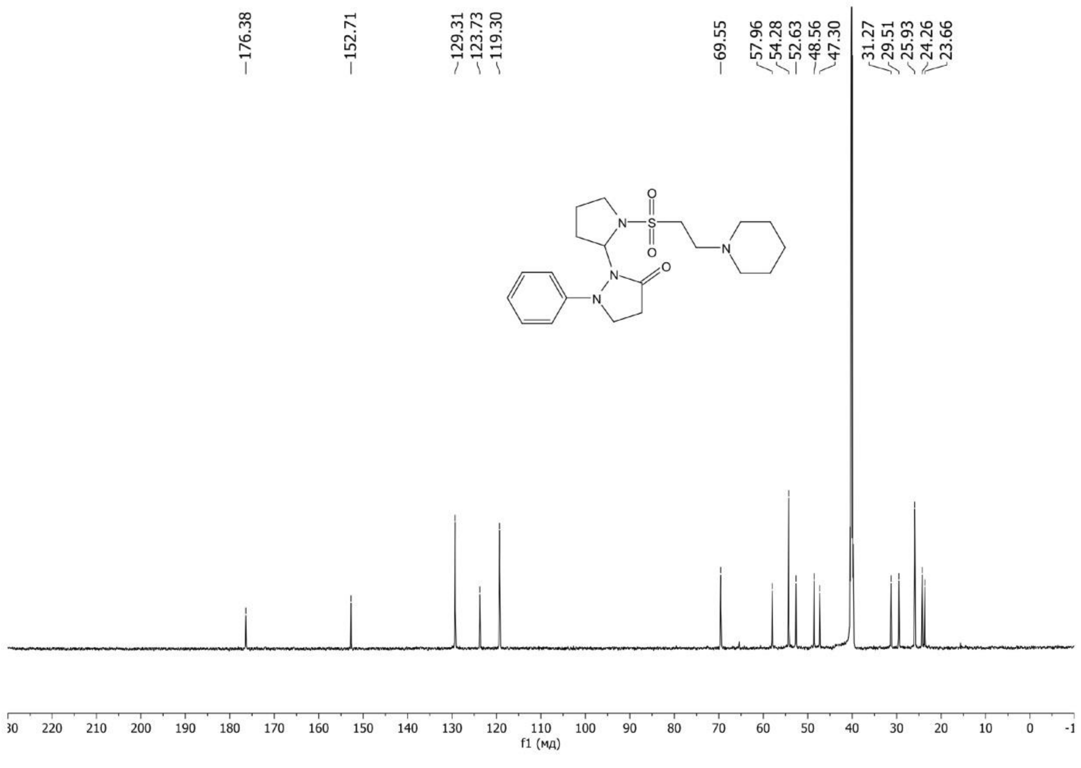

Figure S9. $^{13}C\{^{1}H\}$ NMR (DMSO-d_6, 150 MHz) spectrum of the compound **4b**.

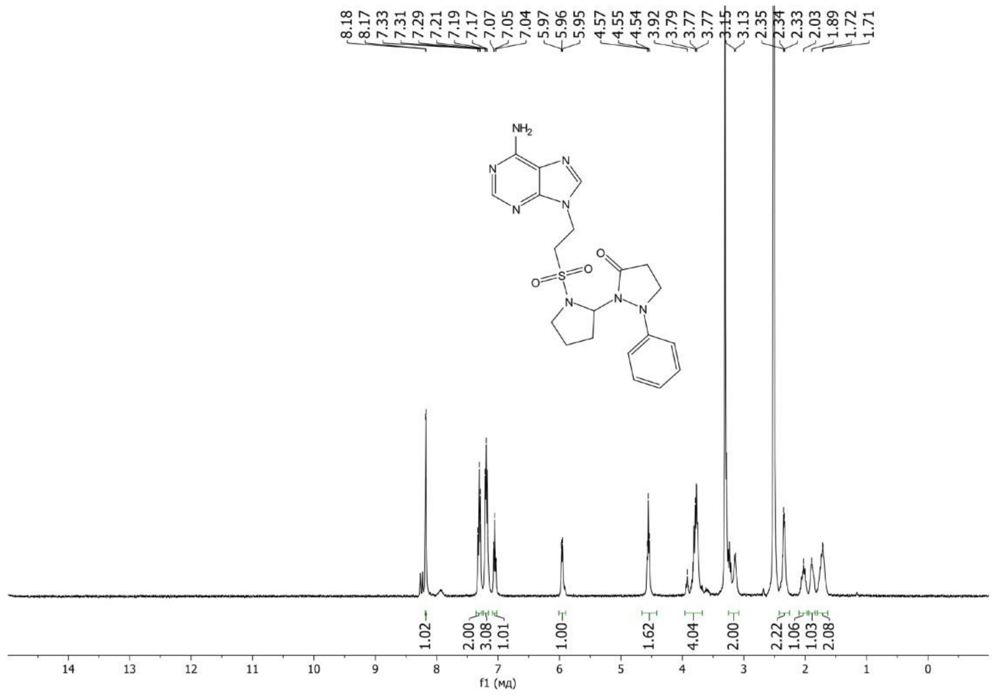

Figure S10. ^{1}H NMR (DMSO-d_6, 600 MHz) spectrum of the compound **4c**.

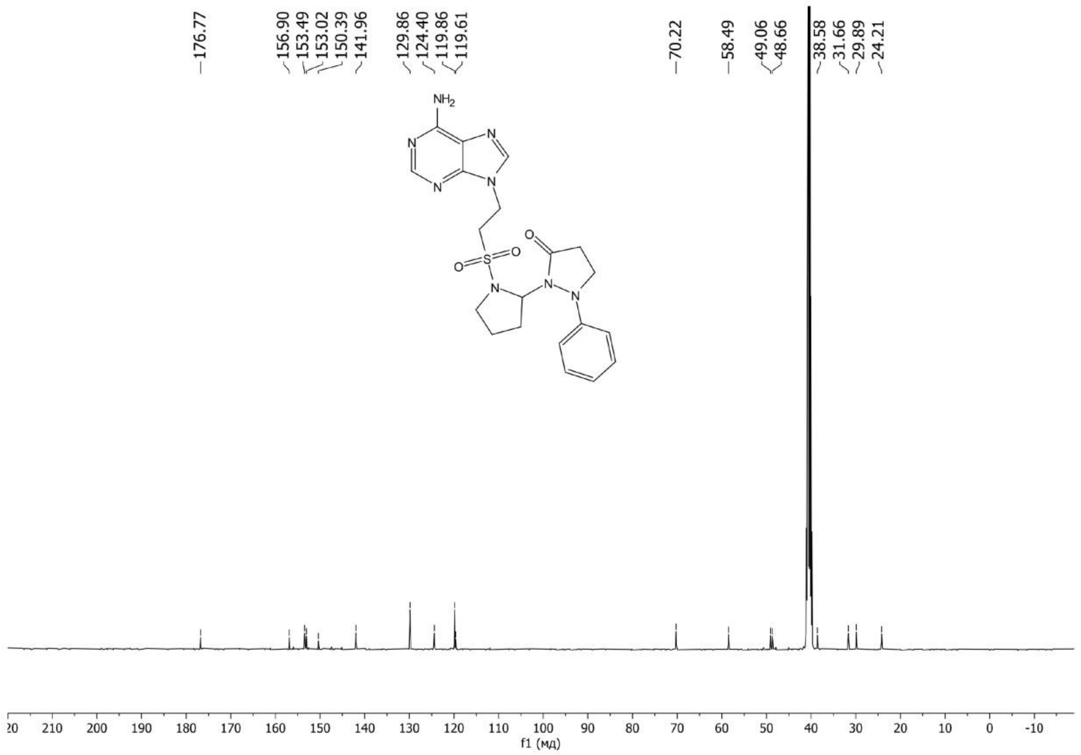

Figure S11. ^{13}C{^{1}H} NMR (DMSO-d_6, 150 MHz) spectrum of the compound **4c**.

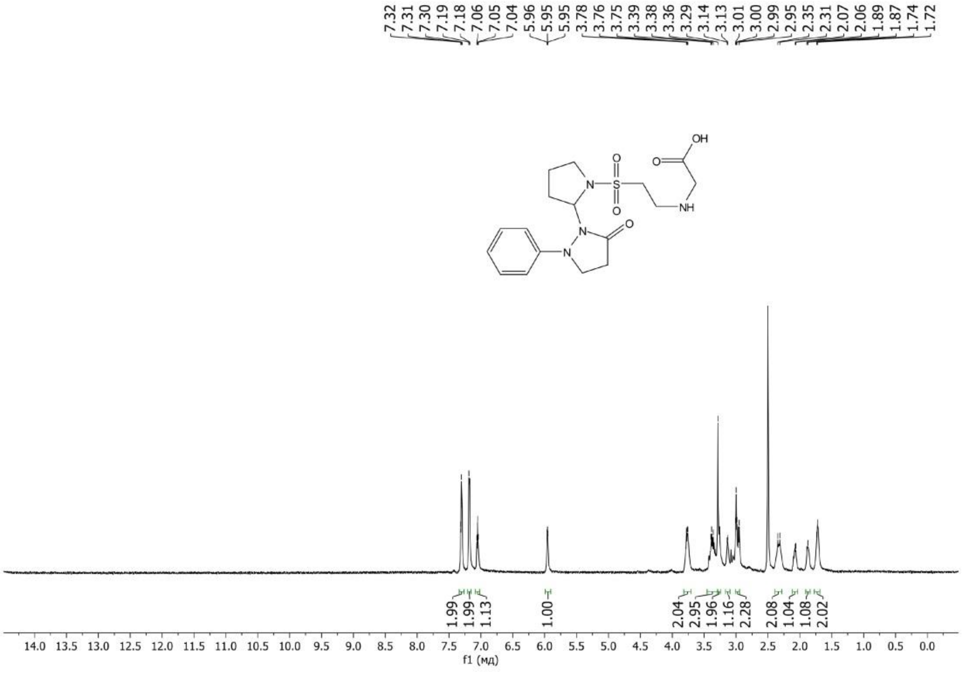

Figure S12. ^{1}H NMR (DMSO-d_6, 600 MHz) spectrum of the compound **4d**.

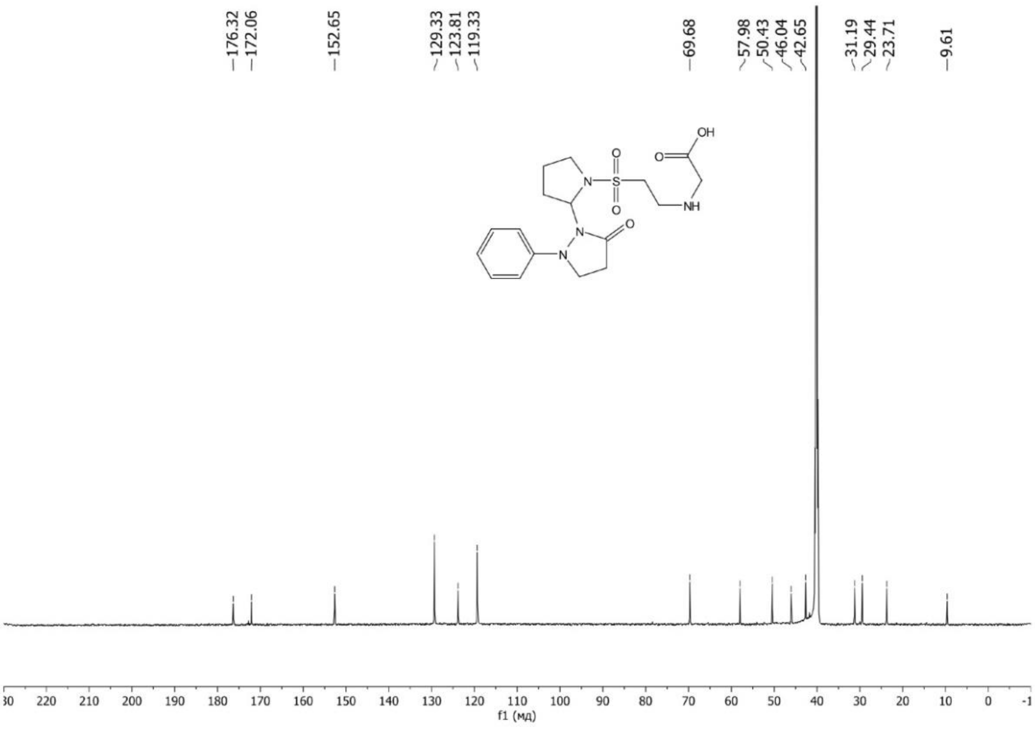

Figure S13. ^{13}C{^{1}H} NMR (DMSO-d_6, 150 MHz) spectrum of the compound **4d**.

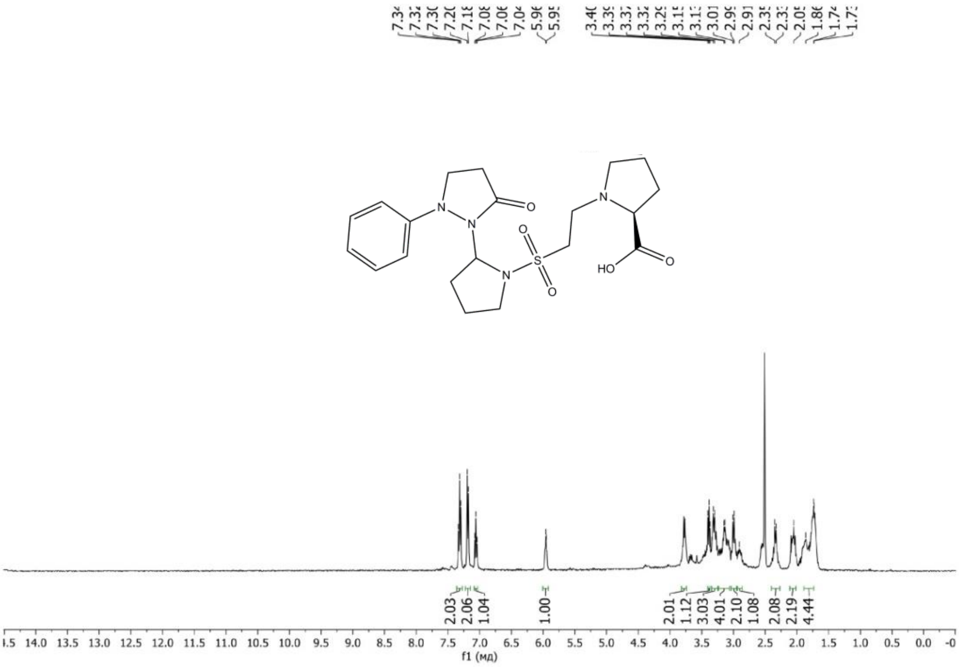

Figure S14. ^{1}H NMR (DMSO-d_6, 600 MHz) spectrum of the compound **4e**.

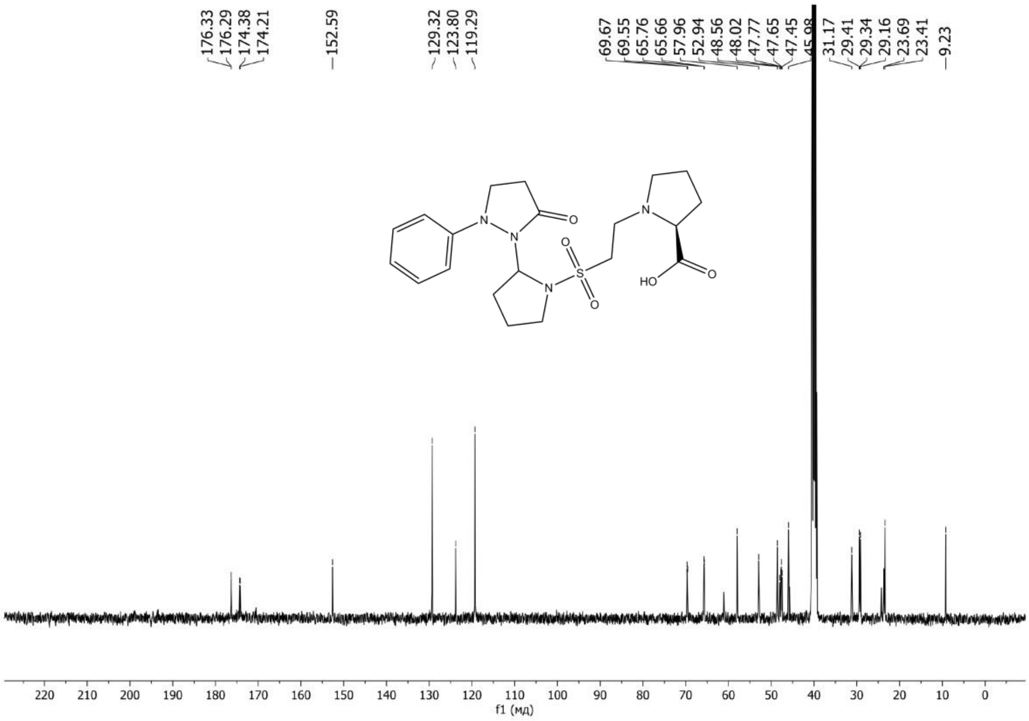

Figure S15. $^{13}C\{^{1}H\}$ NMR (DMSO-d_6, 150 MHz) spectrum of the compound **4e**.

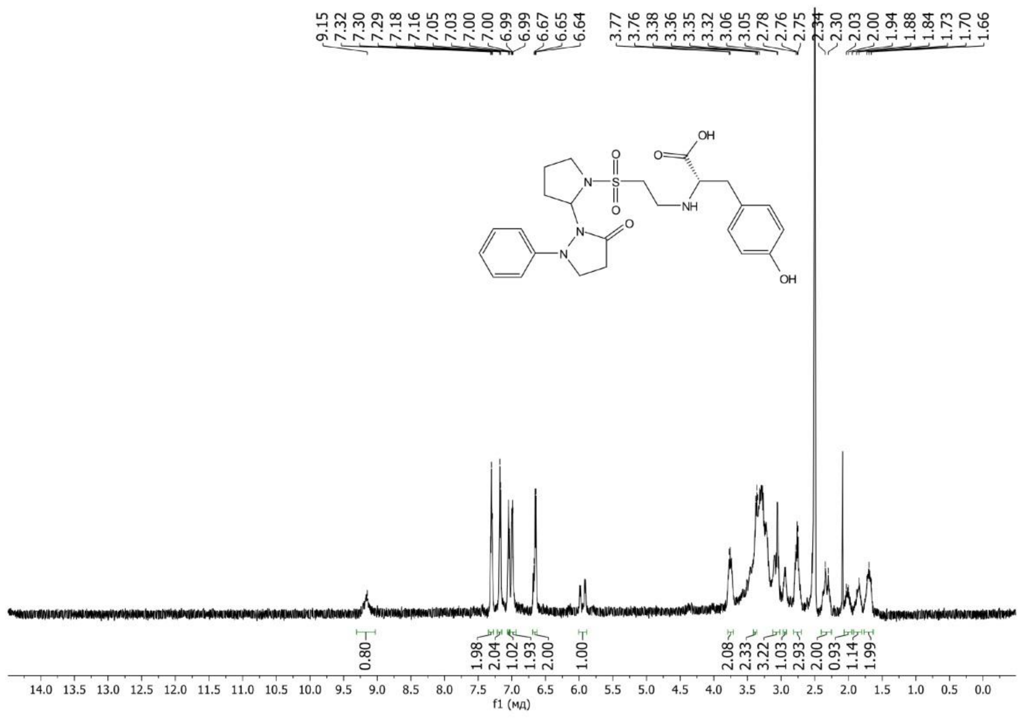

Figure S16. ^{1}H NMR (DMSO-d_6, 600 MHz) spectrum of the compound **4f**.

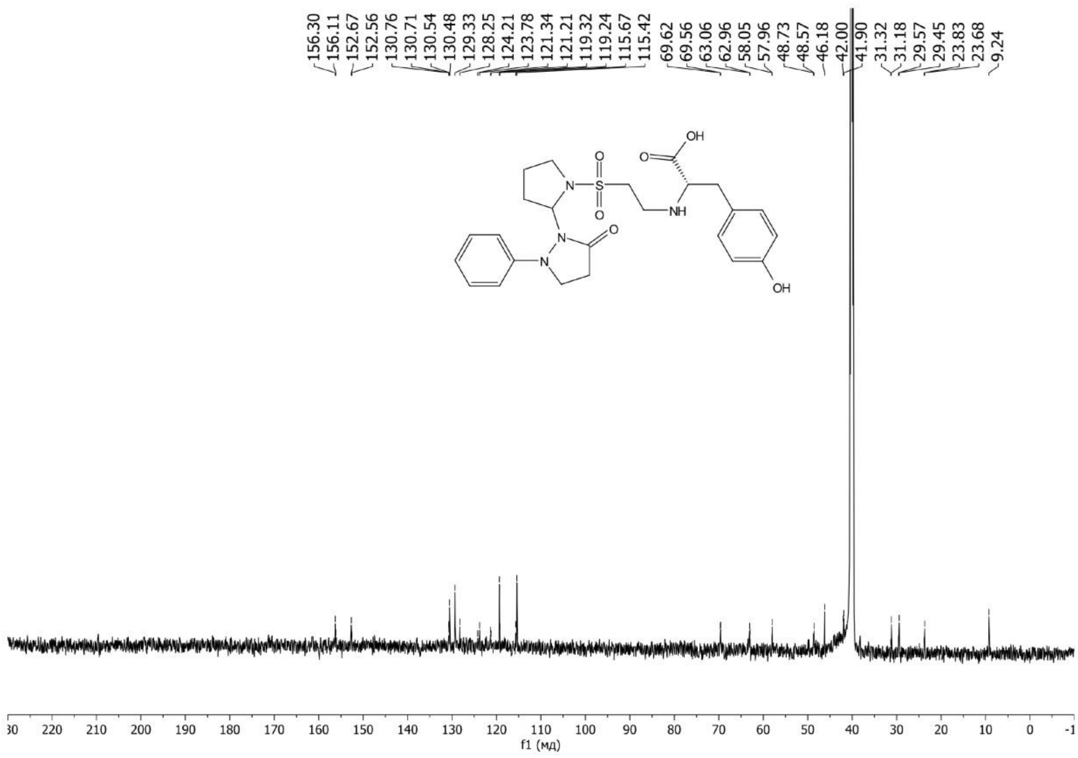

Figure S17. ^{13}C{^{1}H} NMR (DMSO-d_6, 150 MHz) spectrum of the compound **4f**.

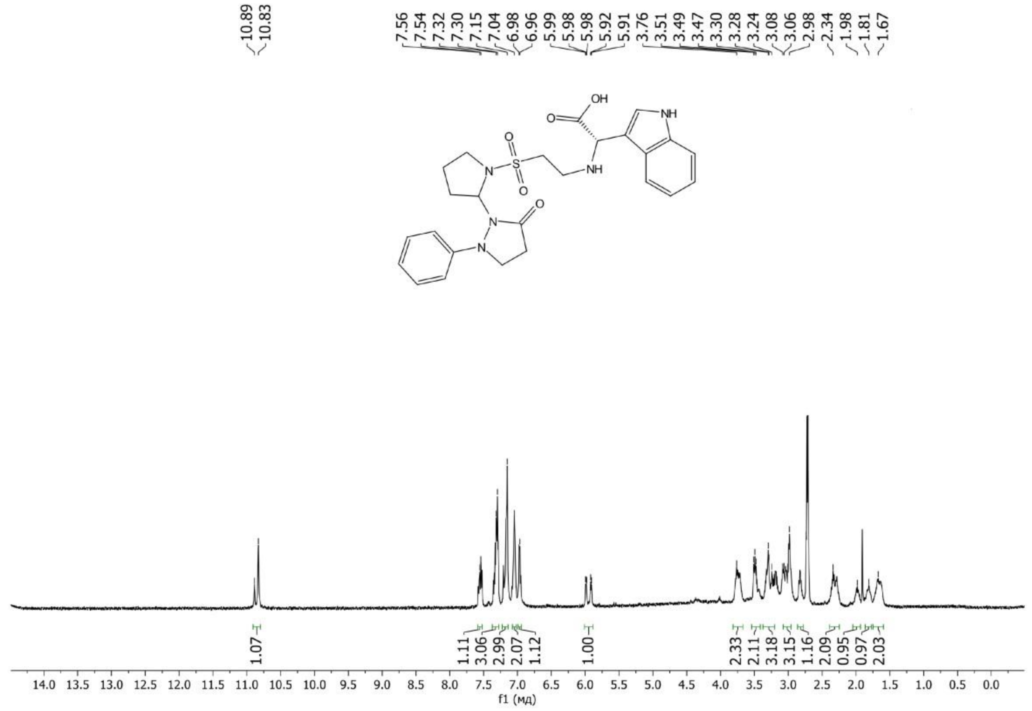

Figure S18. ^{1}H NMR (DMSO-d_6, 600 MHz) spectrum of the compound **4g**.

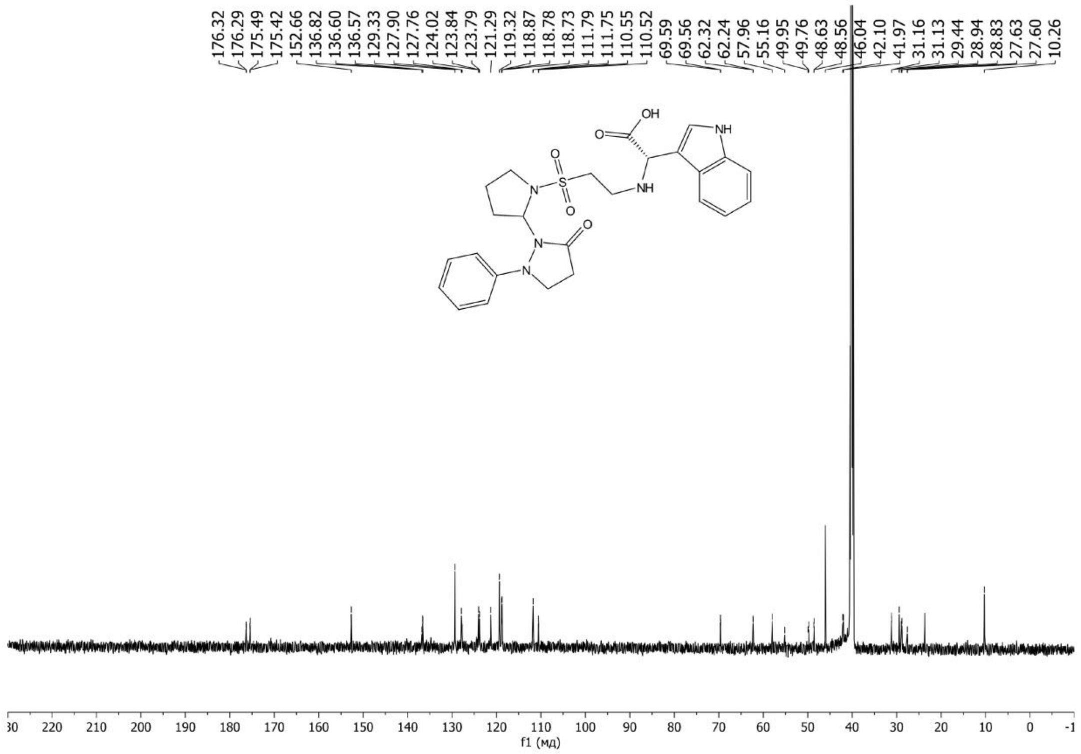

Figure S19. $^{13}C\{^{1}H\}$ NMR (DMSO-d_6, 150 MHz) spectrum of the compound **4g**.

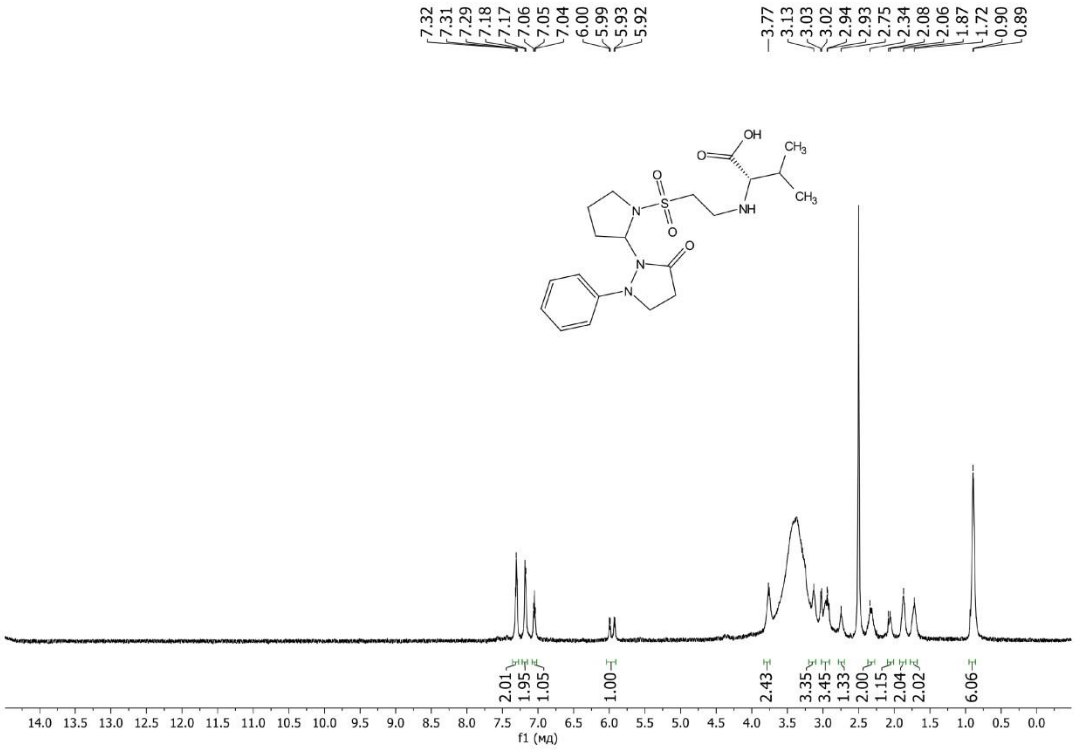

Figure S20. ^{1}H NMR (DMSO-d_6, 600 MHz) spectrum of the compound **4h**.

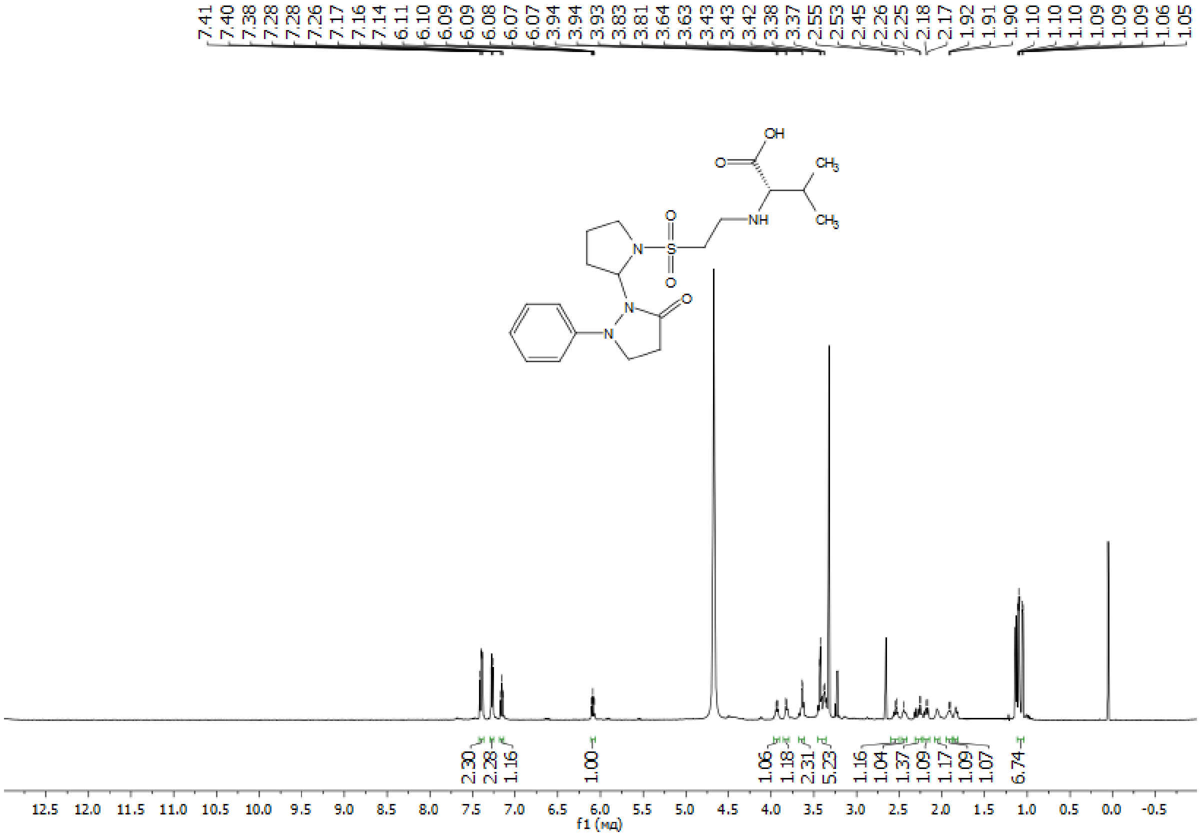

Figure S21. ^{1}H NMR (CD_3OD : DMSO-d_6 / 80% : 20%, 600 MHz) spectrum of the compound **4h**.

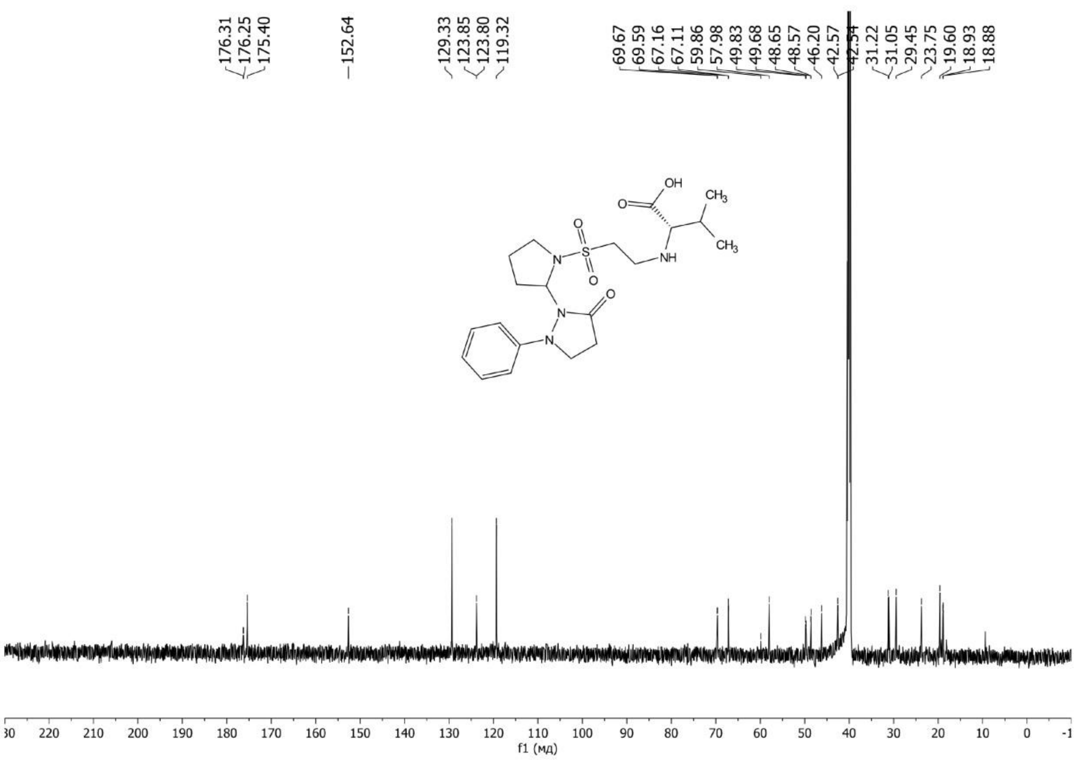

Figure S22. $^{13}C\{^{1}H\}$ NMR (DMSO-d_6, 150 MHz) spectrum of the compound **4h**.

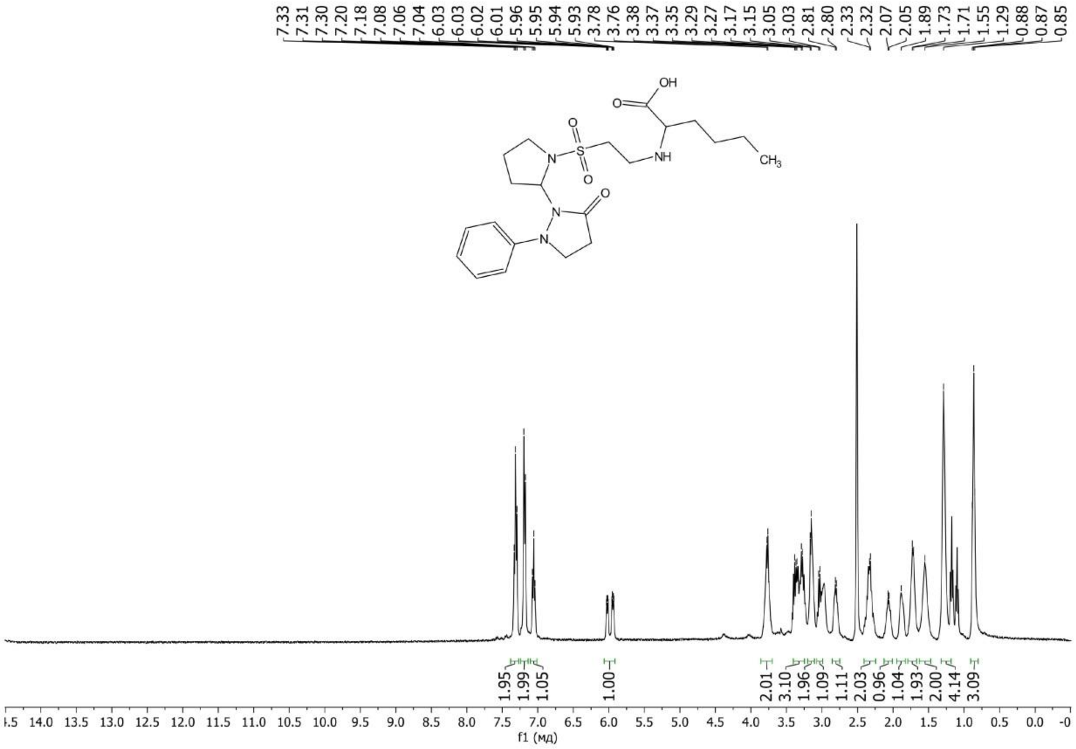

Figure S23. ^{1}H NMR (DMSO-d_6, 600 MHz) spectrum of the compound **4i**.

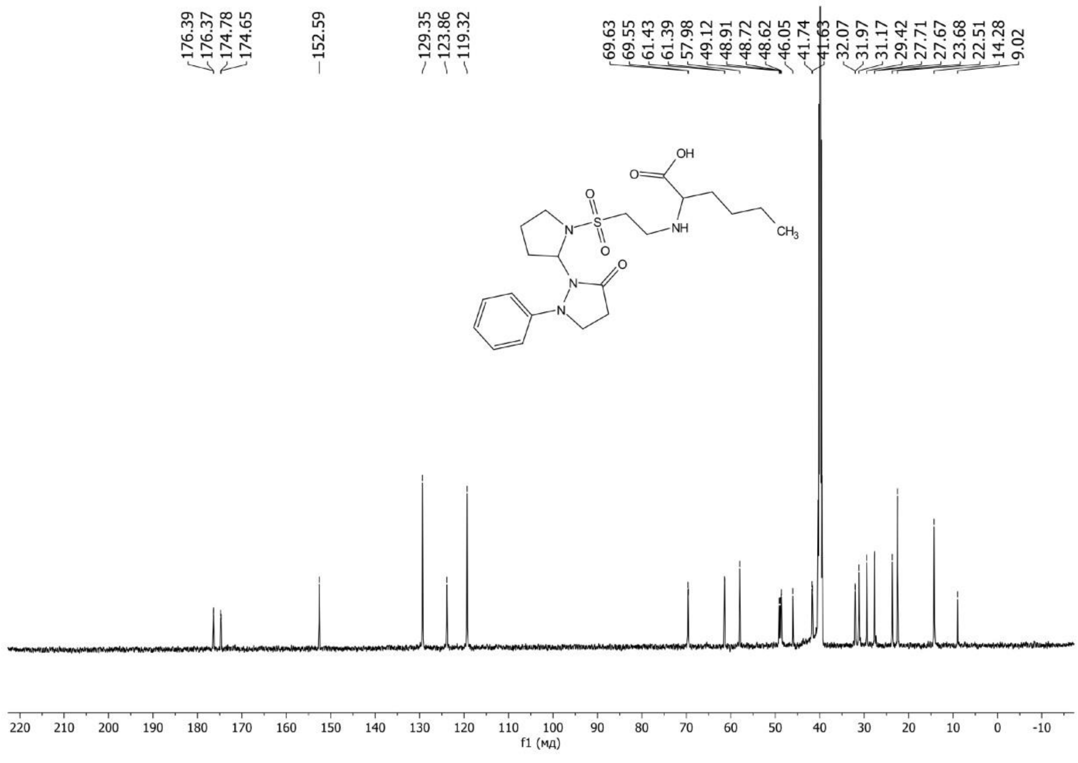

Figure S24. $^{13}C\{^1H\}$ NMR (DMSO-d_6, 150 MHz) spectrum of the compound **4i**.

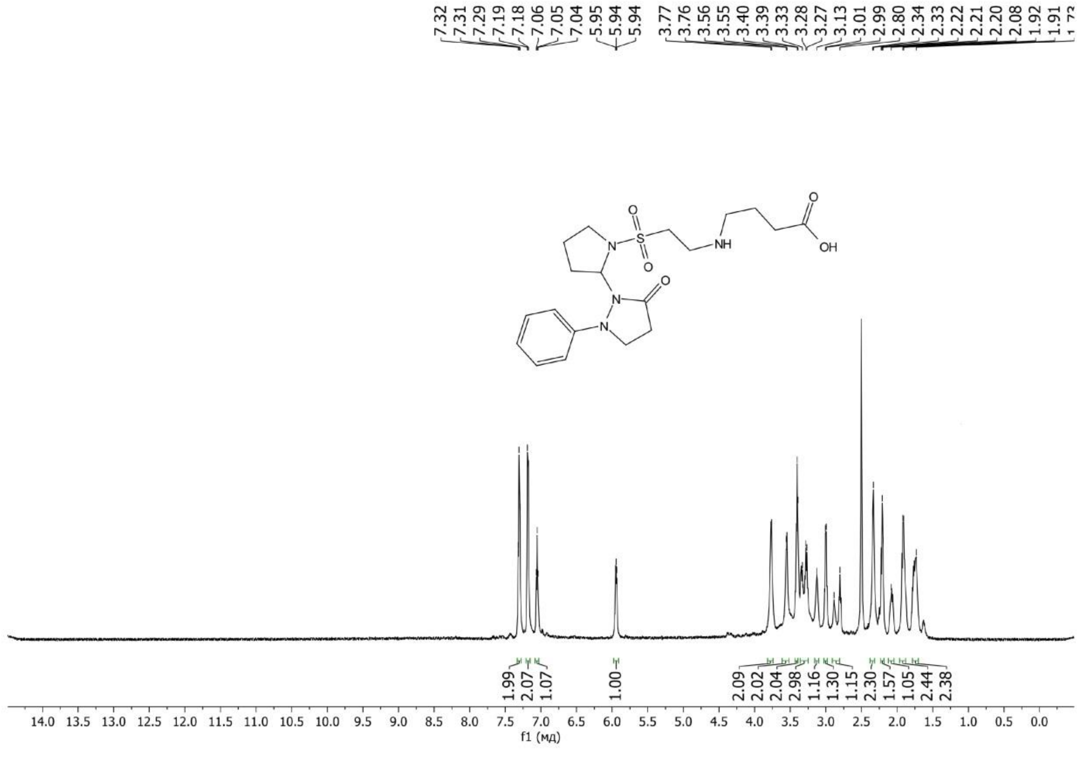

Figure S25. ^1H NMR (DMSO-d_6, 600 MHz) spectrum of the compound **4j**.

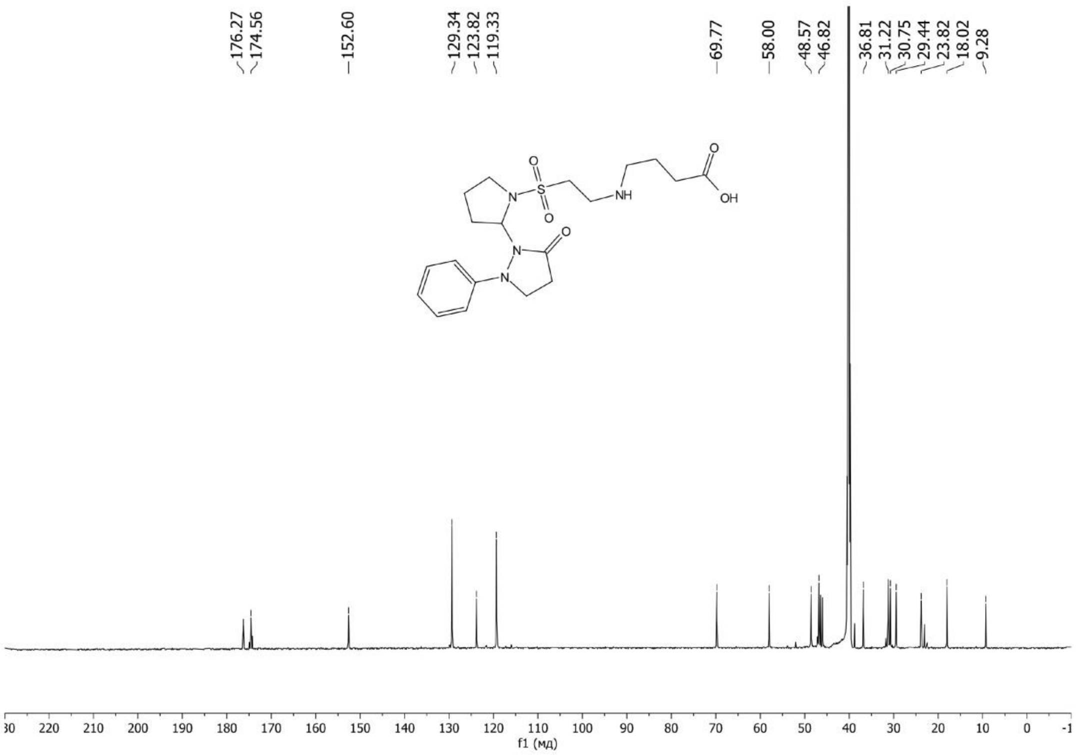

Figure S26. $^{13}C\{^{1}H\}$ NMR (DMSO-d_6, 150 MHz) spectrum of the compound **4j**.

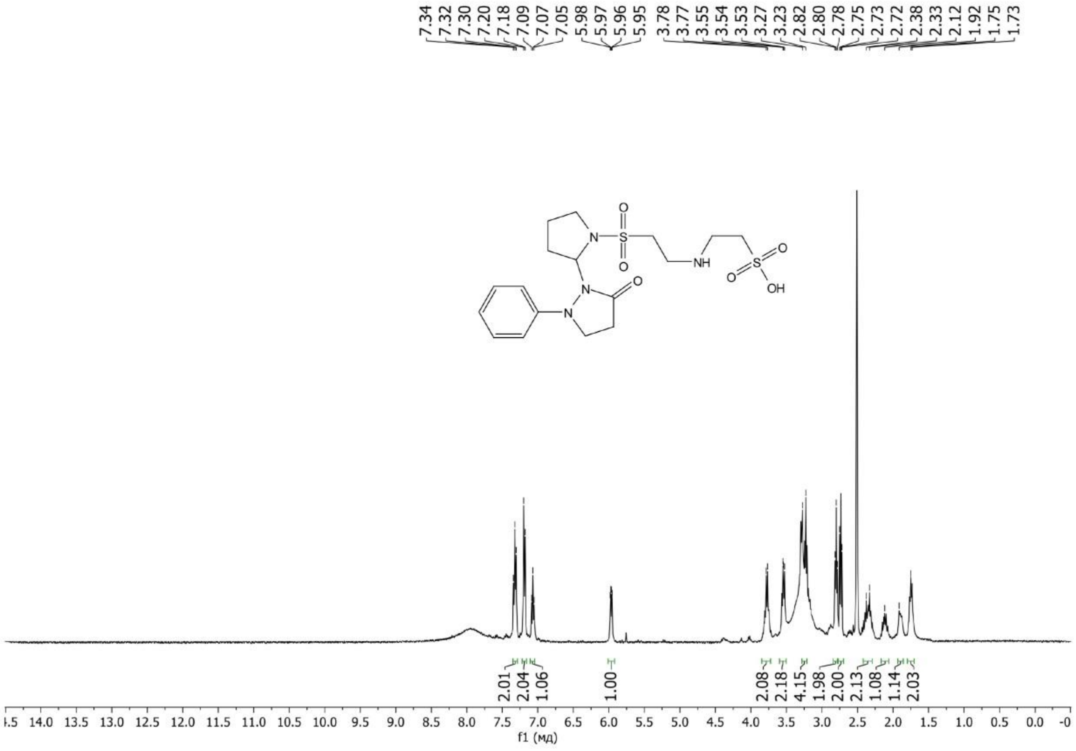

Figure S27. ^{1}H NMR (DMSO-d_6, 600 MHz) spectrum of the compound **4k**.

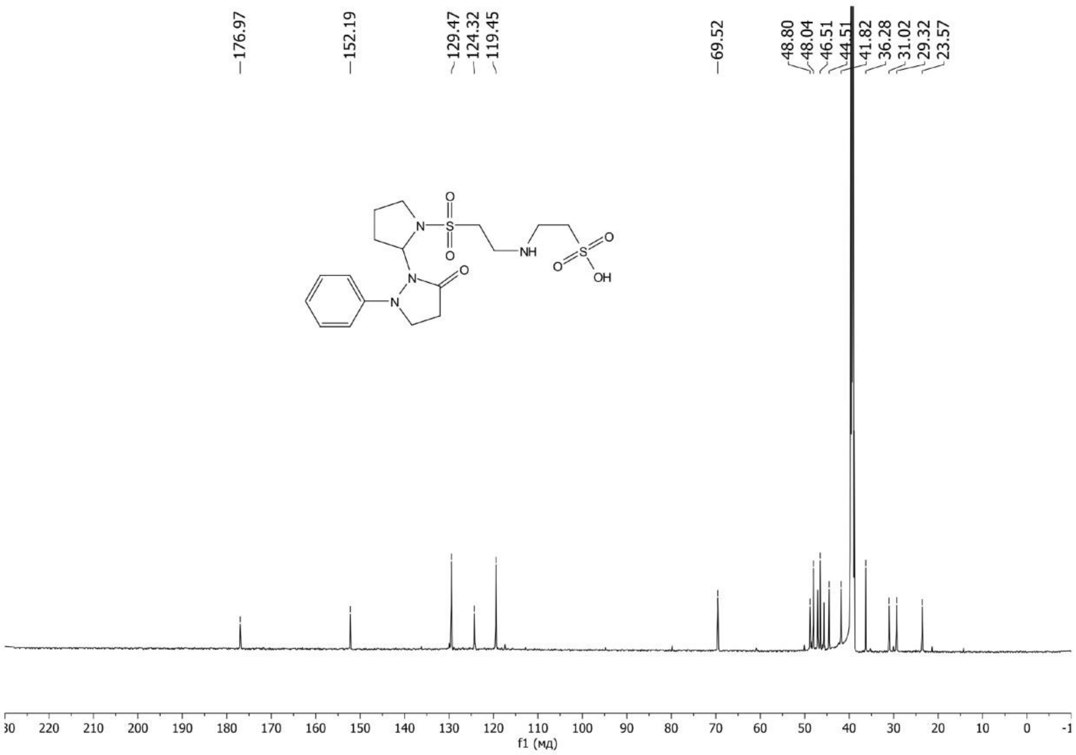

Figure S28. $^{13}C\{^{1}H\}$ NMR (DMSO-d_6, 150 MHz) spectrum of the compound **4k**.

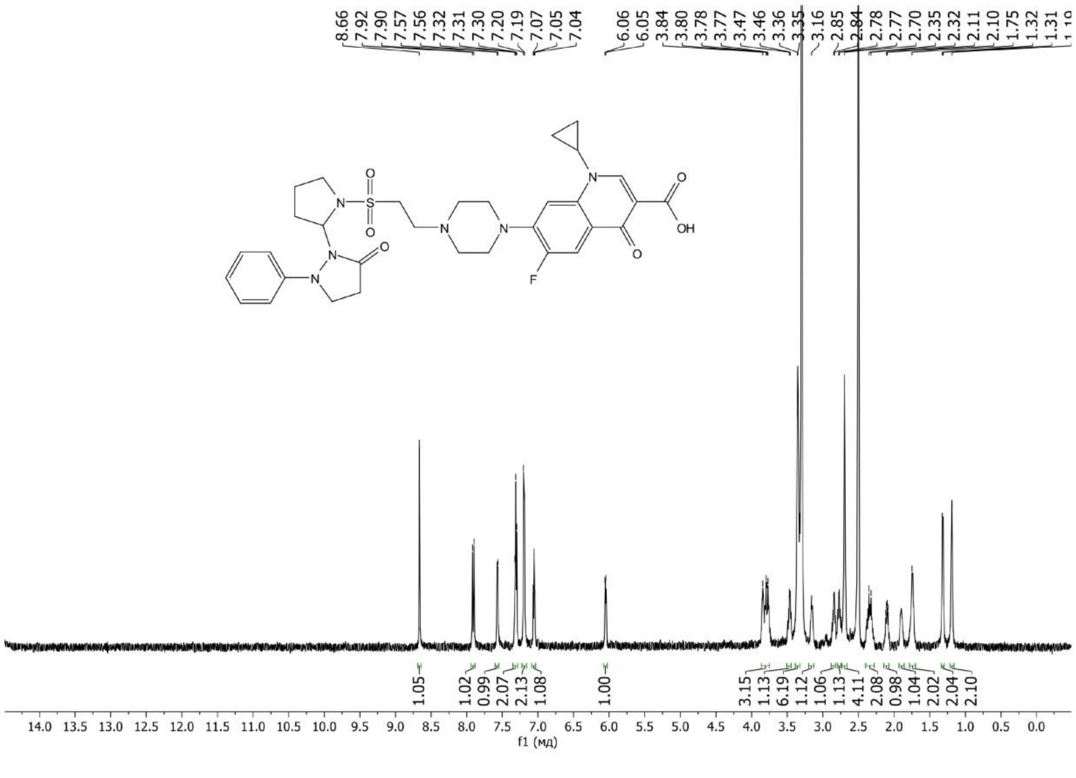

Figure S29. ^{1}H NMR (DMSO-d_6, 600 MHz) spectrum of the compound **4l**.

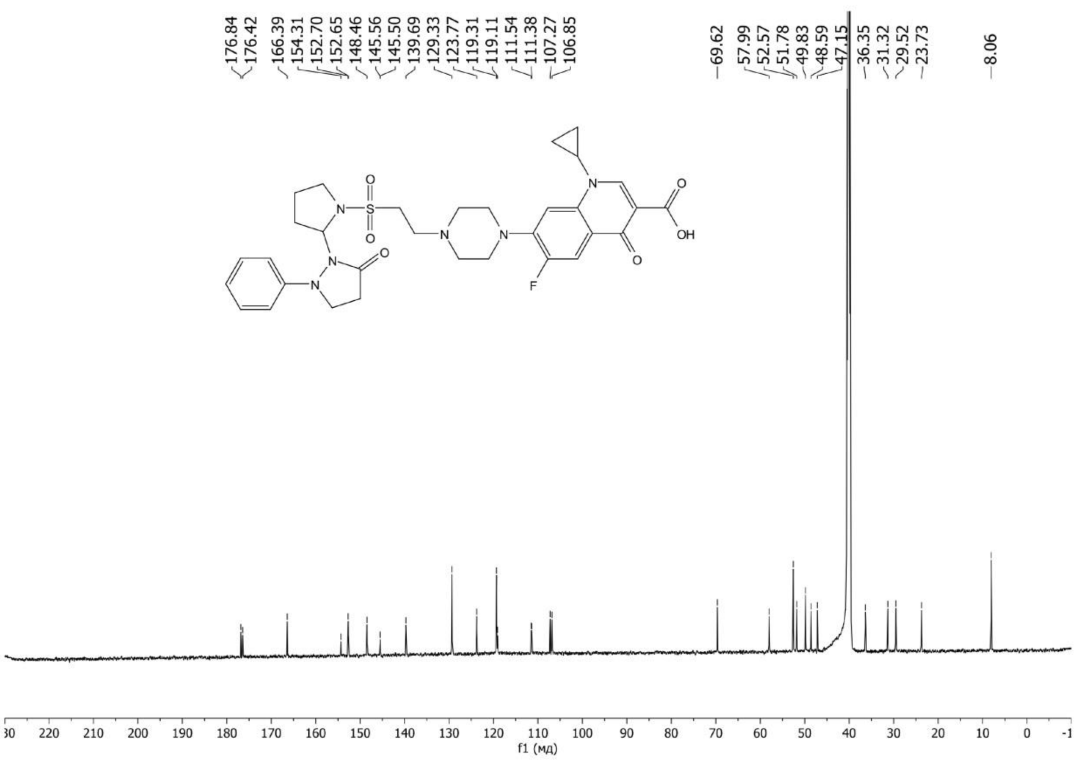

Figure S30. $^{13}C\{^{1}H\}$ NMR (DMSO-d_6, 150 MHz) spectrum of the compound **4l**.

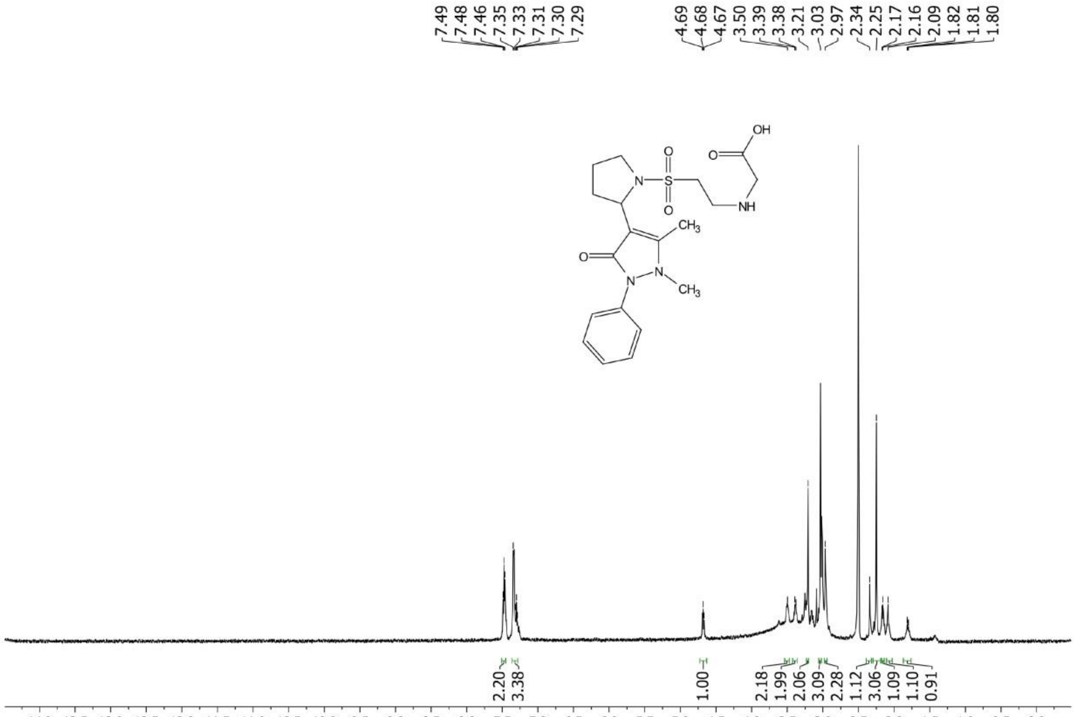

Figure S31. ^{1}H NMR (DMSO-d_6, 600 MHz) spectrum of the compound **4m**.

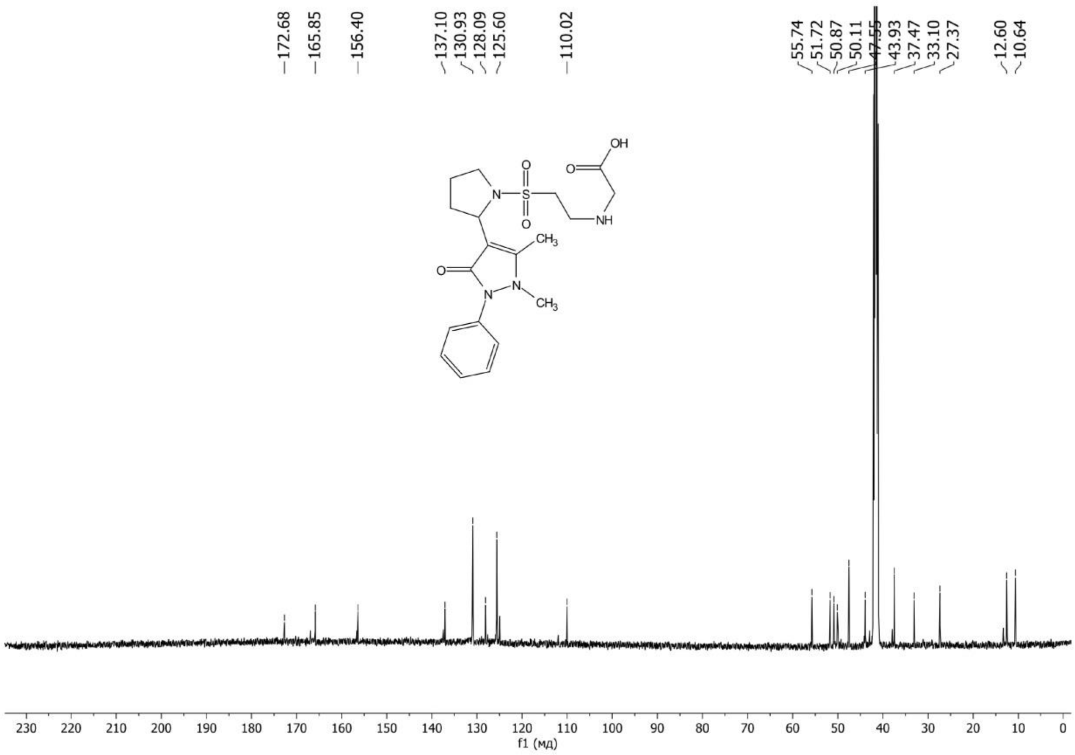

Figure S32. $^{13}C\{^{1}H\}$ NMR (DMSO-d_6, 150 MHz) spectrum of the compound **4m**.

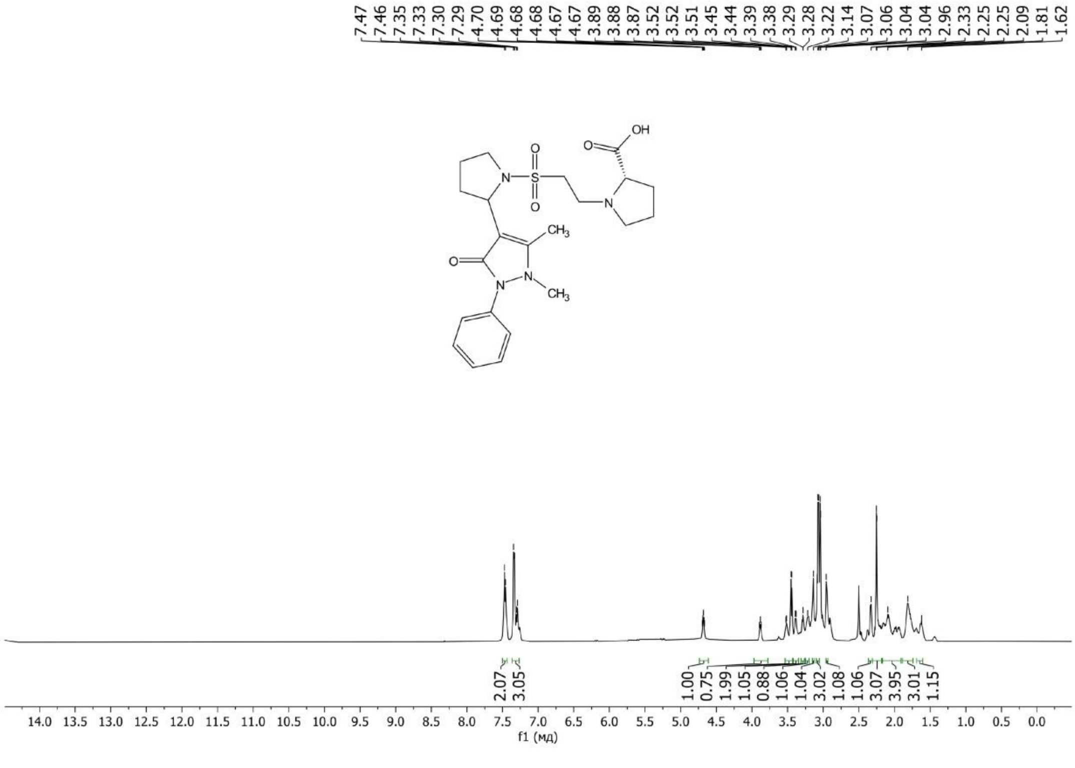

Figure S33. ^{1}H NMR (DMSO-d_6, 600 MHz) spectrum of the compound **4n**.

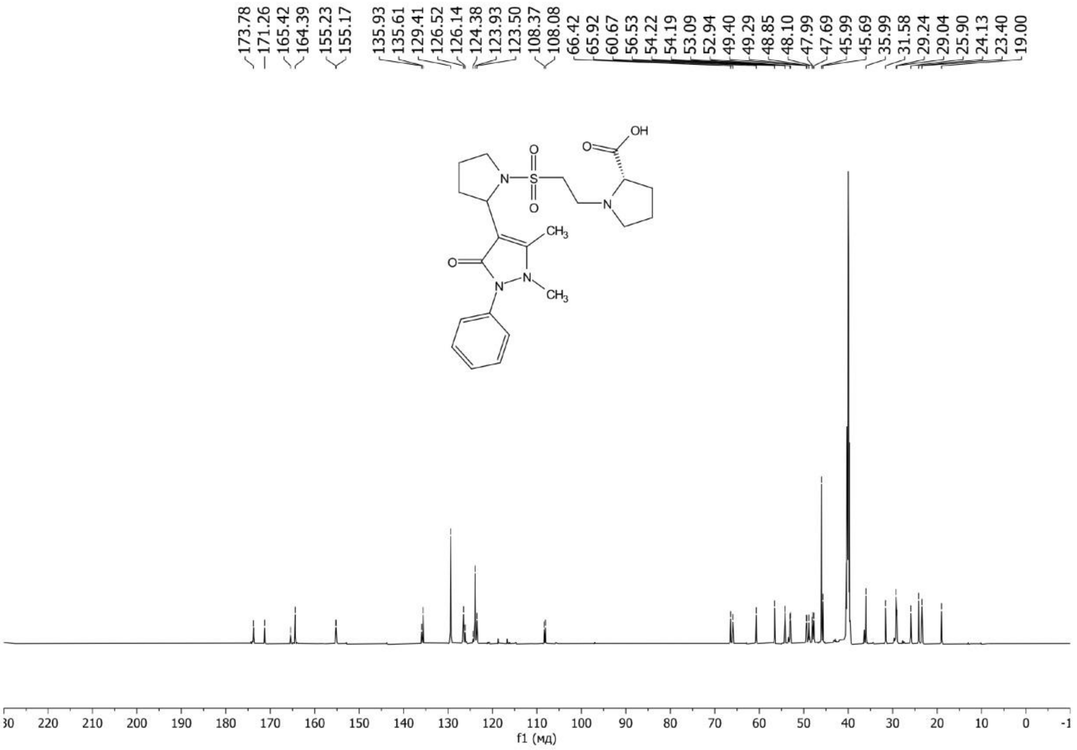

Figure S34. $^{13}C\{^{1}H\}$ NMR (DMSO-d_6, 150 MHz) spectrum of the compound **4n**.

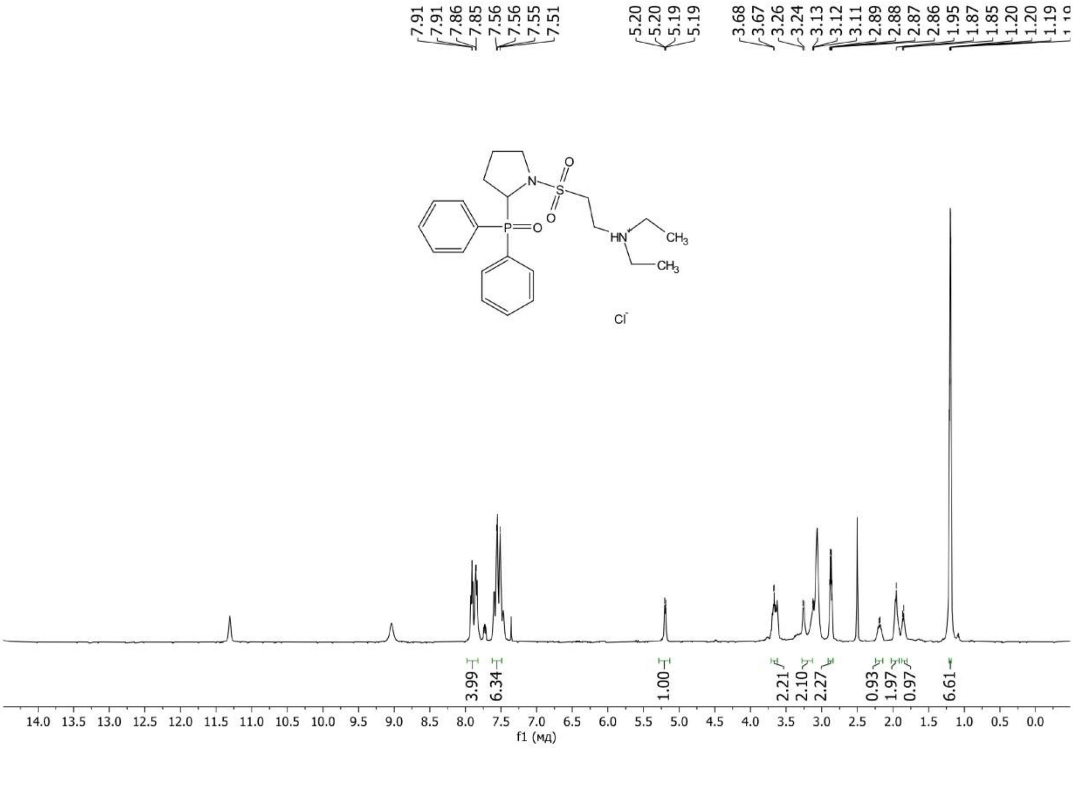

Figure S35. ^{1}H NMR (DMSO-d_6, 600 MHz) spectrum of the compound **4o**.

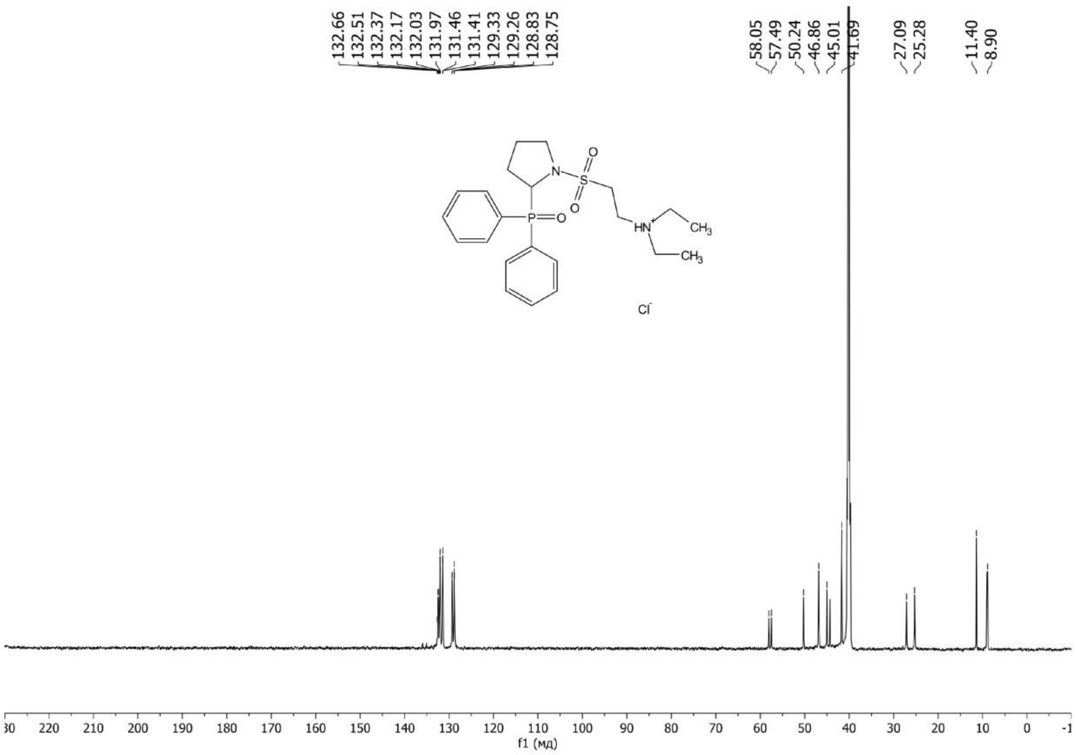

Figure S36. $^{13}C\{^{1}H\}$ NMR (DMSO-d_6, 150 MHz) spectrum of the compound **4o**.

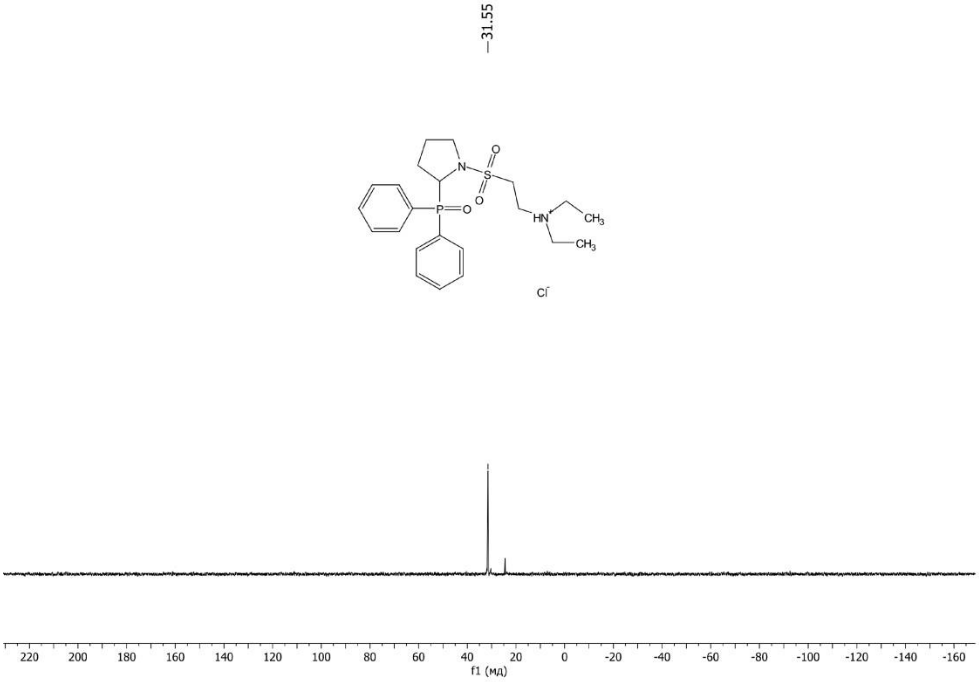

Figure S37. $^{31}P\{^{1}H\}$ NMR spectrum (DMSO-d_6, 161.9 MHz) of the compound **4o**.

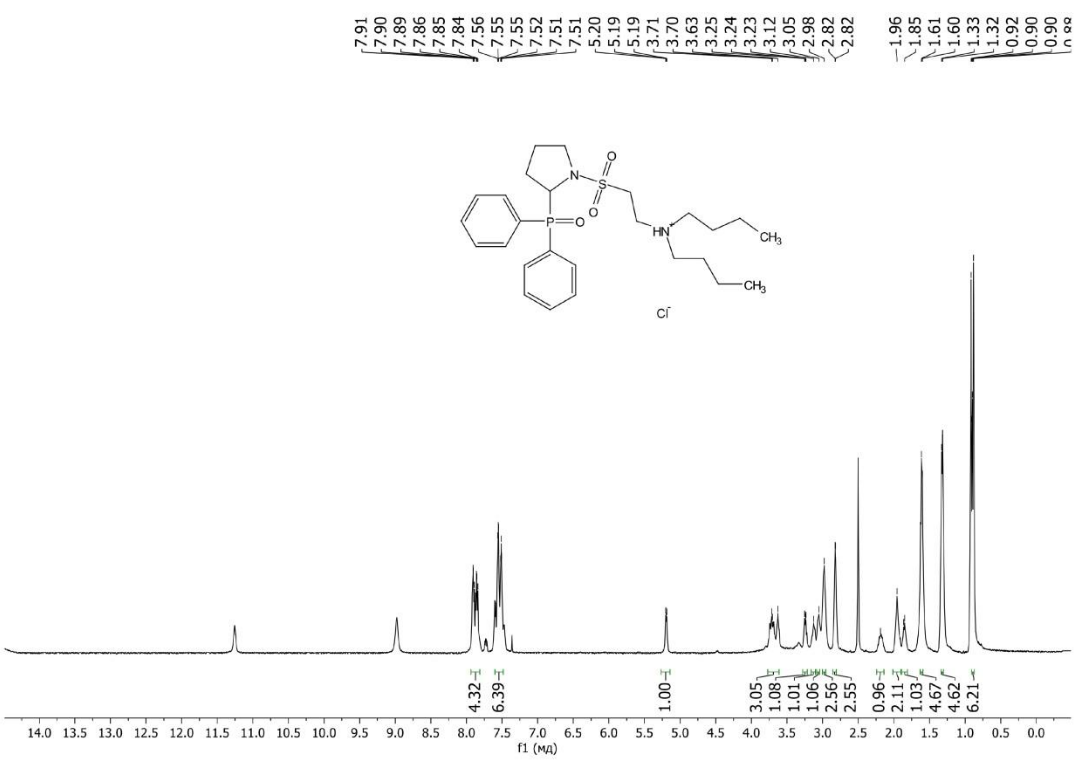

Figure S38. ^{1}H NMR (DMSO-d_6, 600 MHz) spectrum of the compound **4p**.

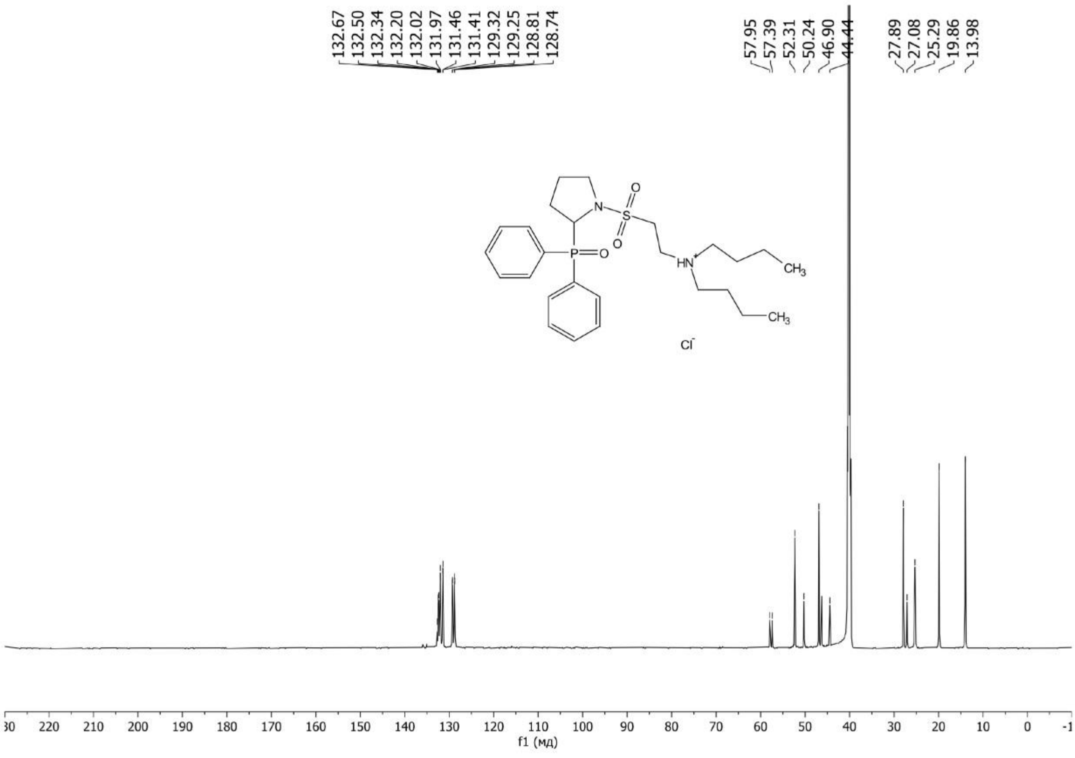

Figure S39. $^{13}C\{^{1}H\}$ NMR (DMSO-d_6, 150 MHz) spectrum of the compound **4p**.

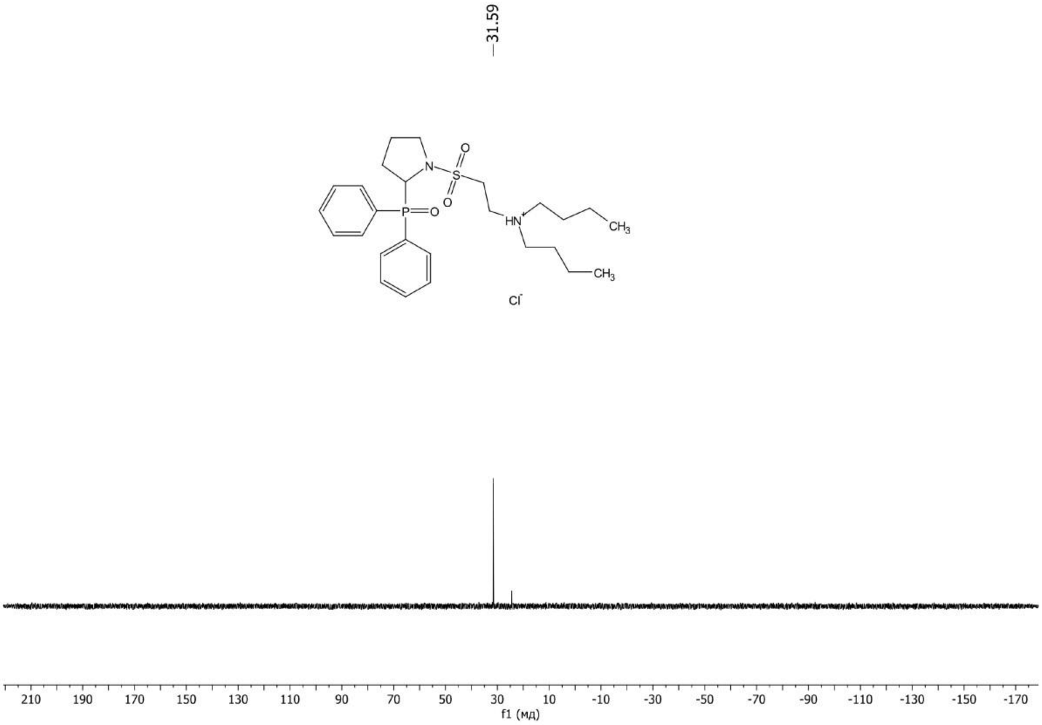

Figure S40. $^{31}P\{^{1}H\}$ NMR spectrum (DMSO-d_6, 161.9 MHz) of the compound **4p**.

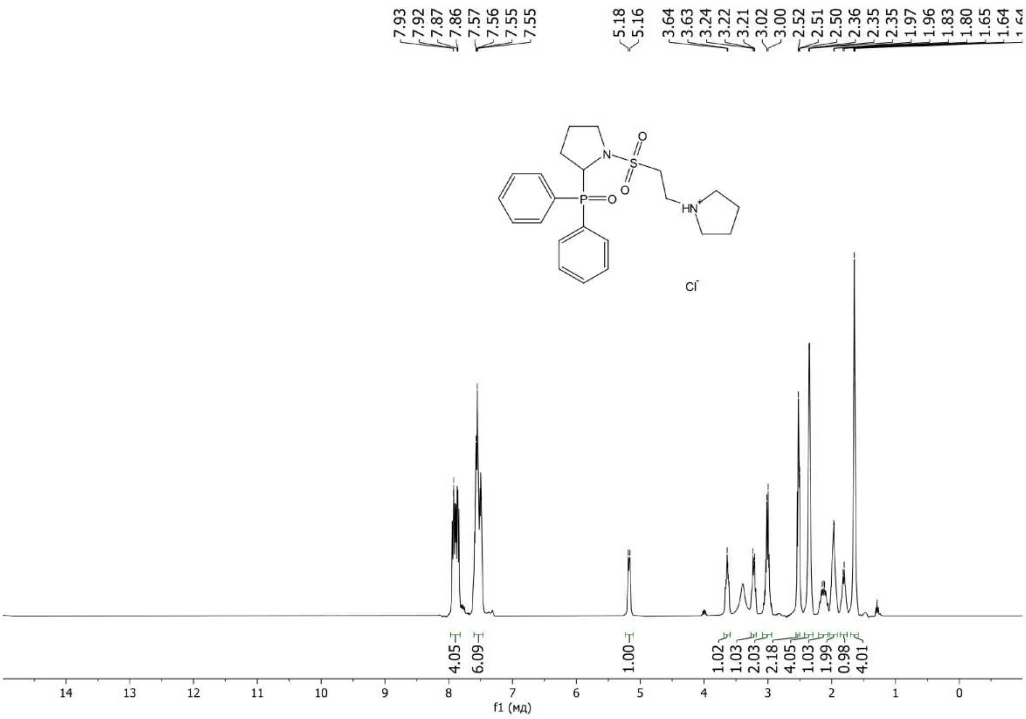

Figure S41. ^{1}H NMR (DMSO-d_6, 600 MHz) spectrum of the compound **4q**.

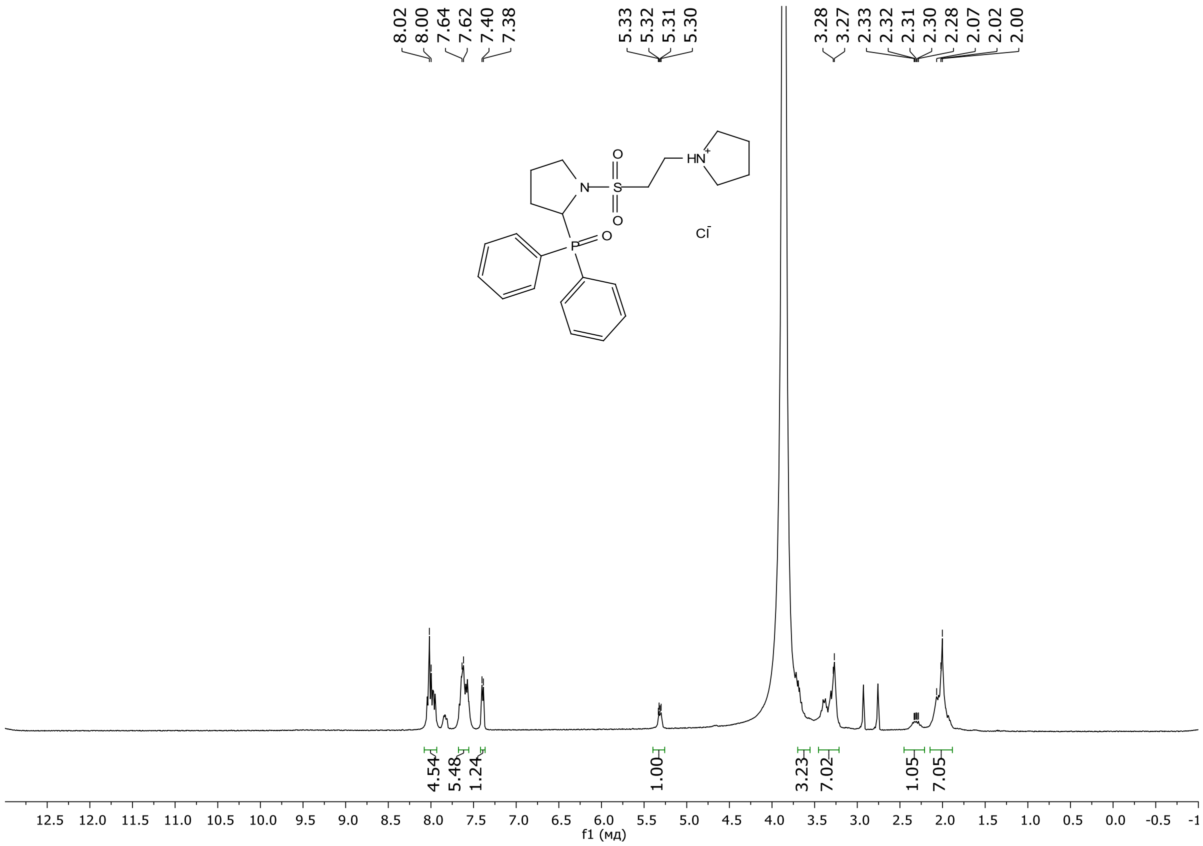

Figure S42. ^{1}H NMR (DMF-d_7, 600 MHz) spectrum of the compound **4q**.

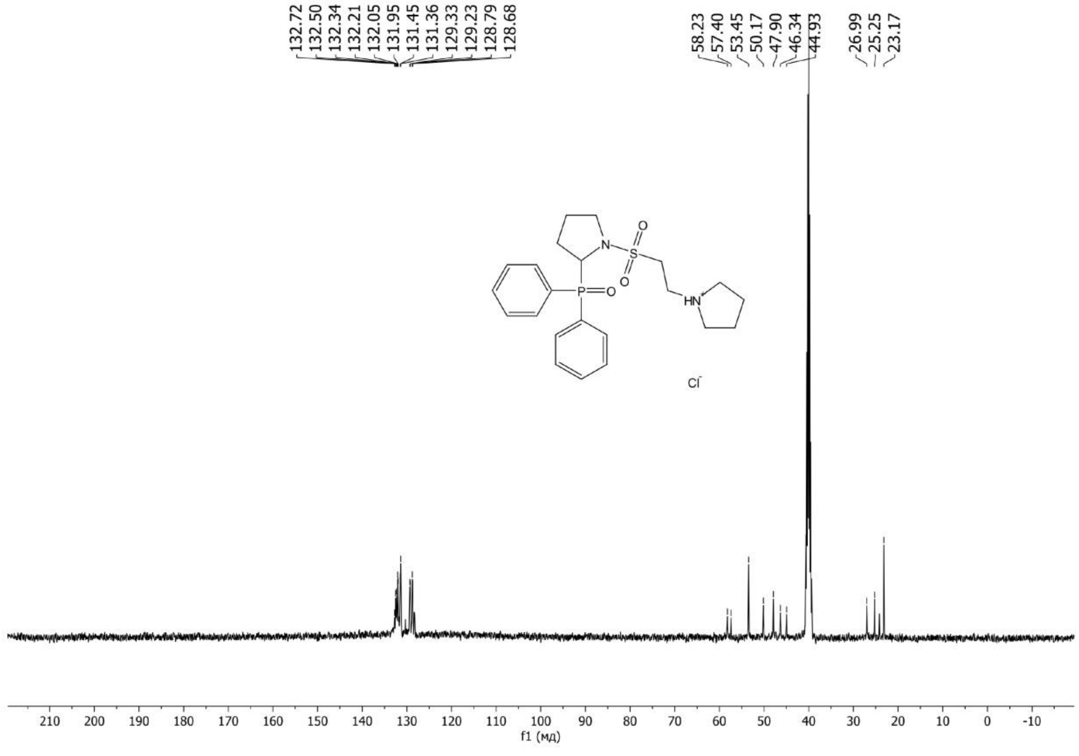

Figure S43. ^{13}C{^{1}H} NMR (DMSO-d_6, 150 MHz) spectrum of the compound **4q**.

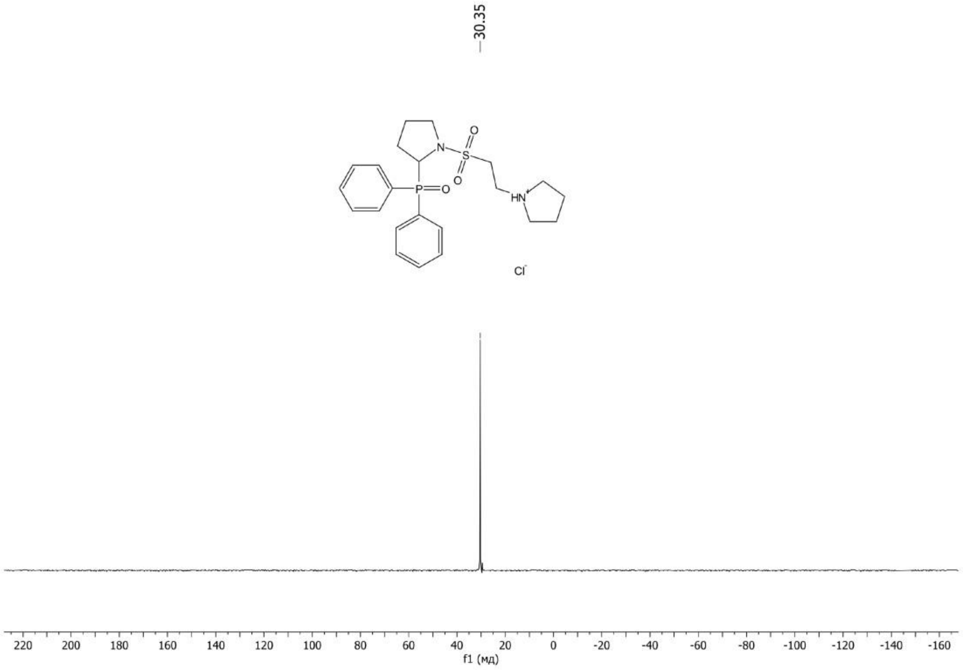

Figure S44. $^{31}P\{^{1}H\}$ NMR spectrum (DMSO-d_6, 161.9 MHz) of the compound **4q**.

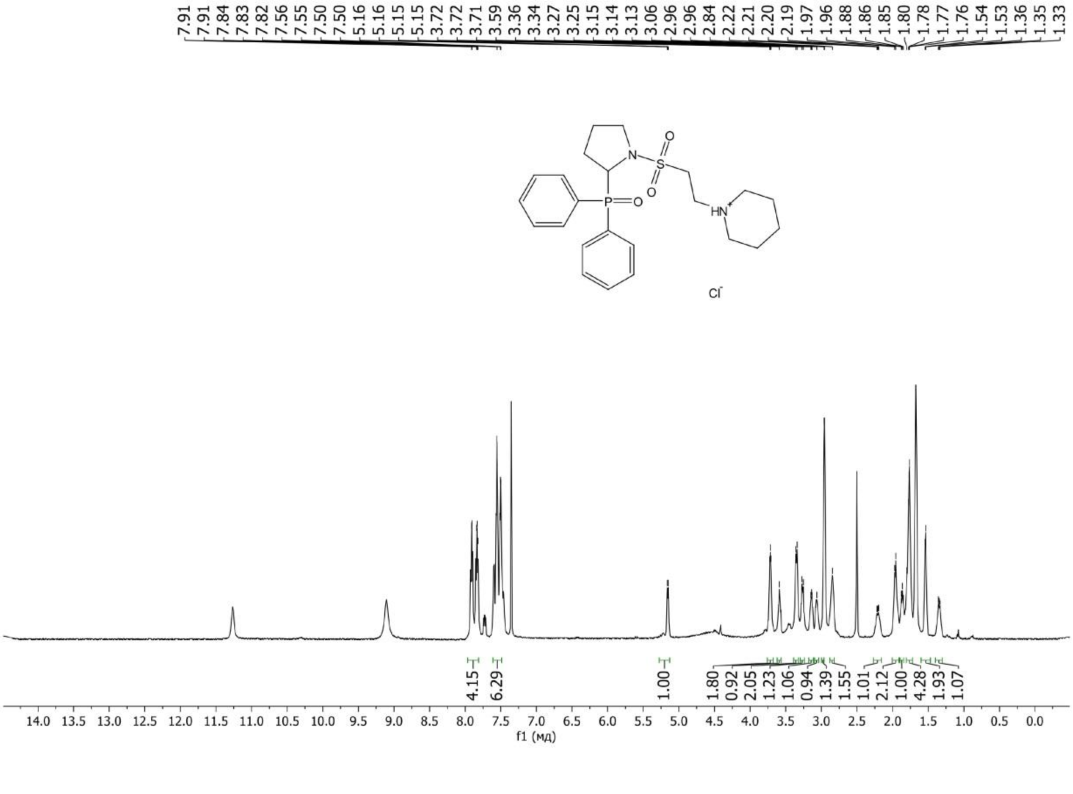

Figure S45. ^{1}H NMR (DMSO-d_6, 600 MHz) spectrum of the compound **4r**.

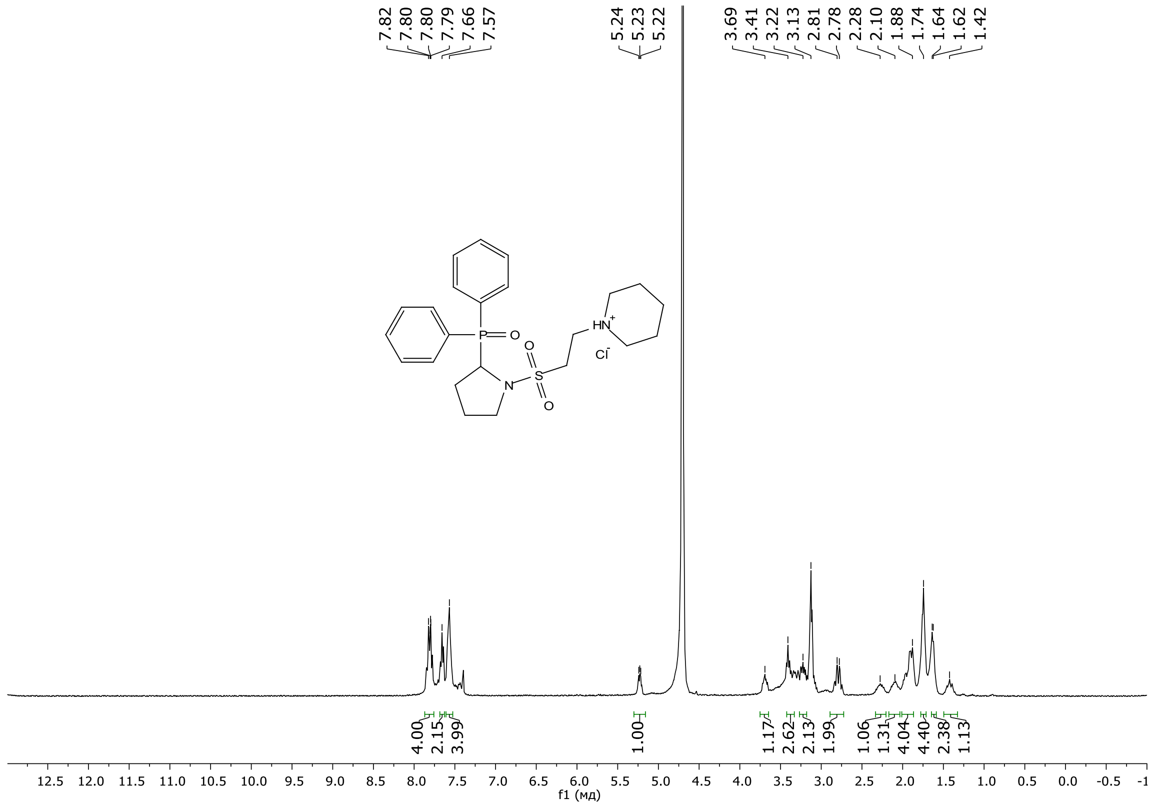

Figure S46. ^{1}H NMR (D_2O, 600 MHz) spectrum of the compound **4r**.

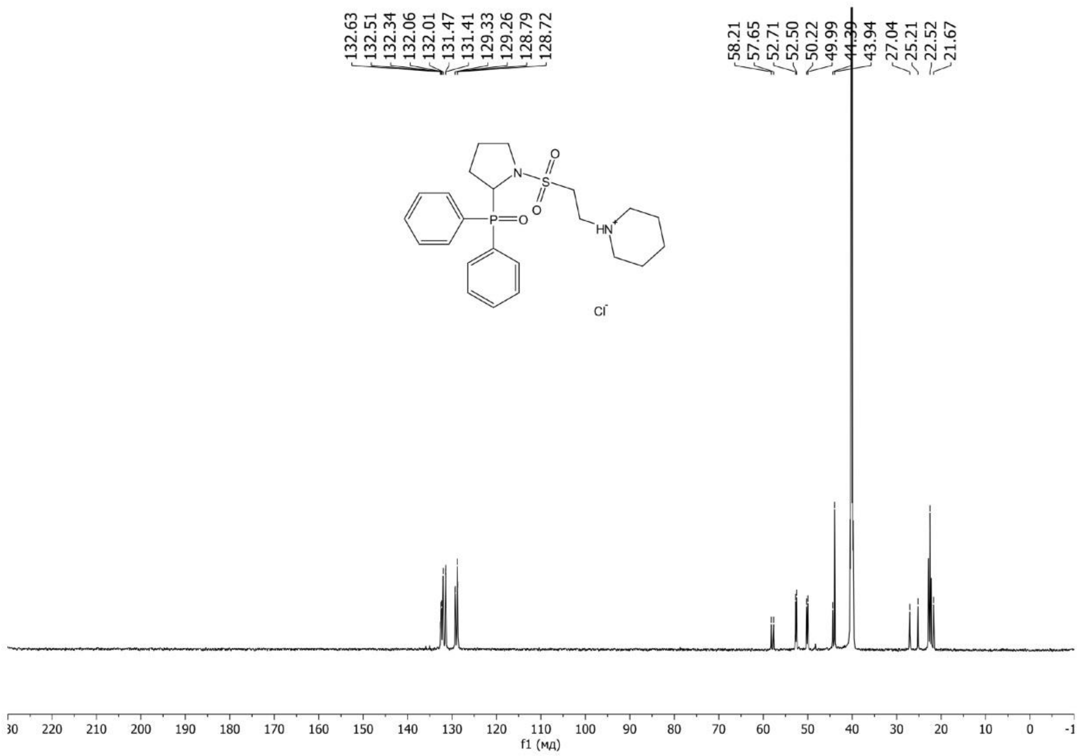

Figure S47. $^{13}C\{^{1}H\}$ NMR (DMSO-d_6, 150 MHz) spectrum of the compound **4r**.

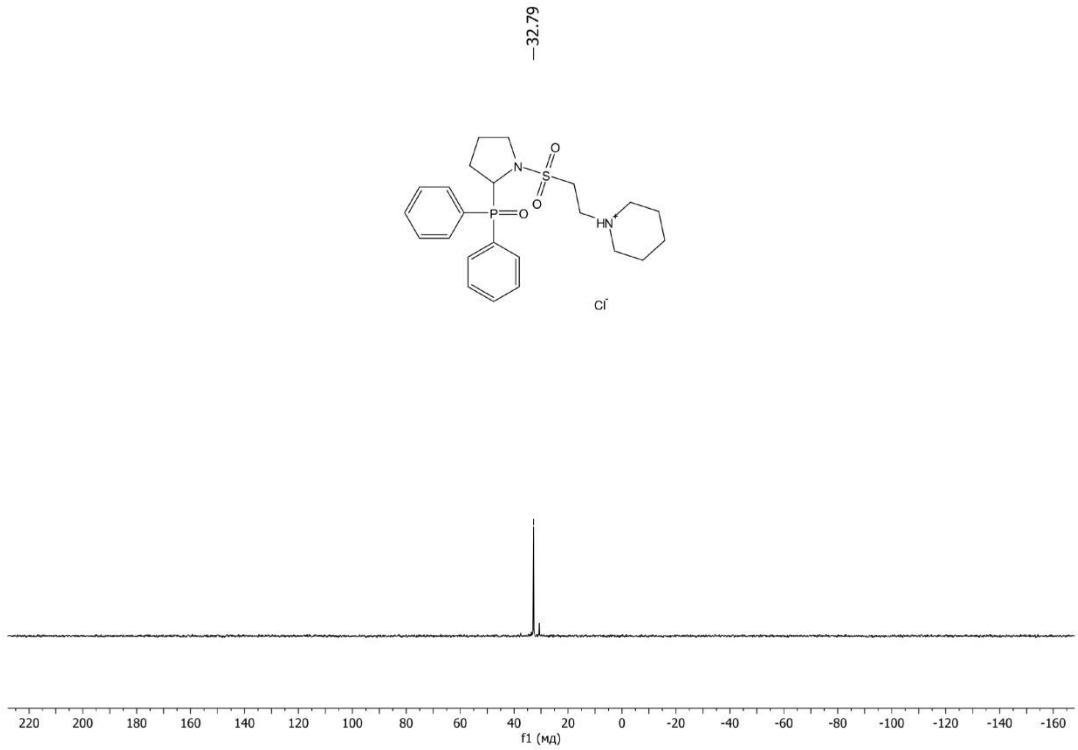

Figure S48. $^{31}P\{^{1}H\}$ NMR spectrum (DMSO-d_6, 161.9 MHz) of the compound **4r**.

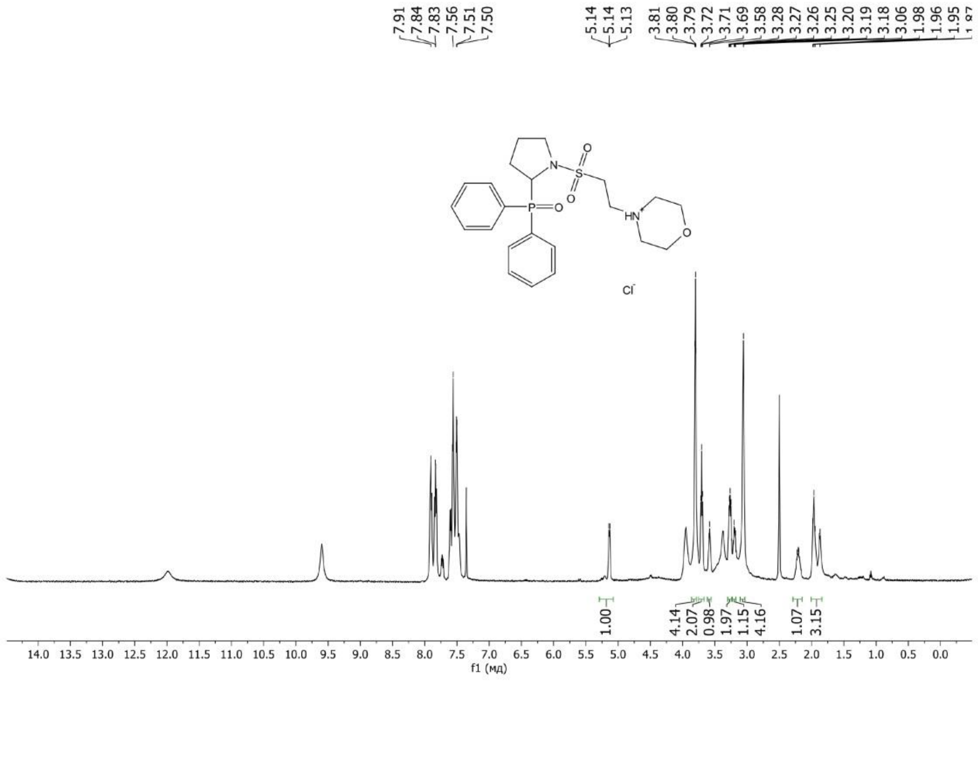

Figure S49. ^{1}H NMR (DMSO-d_6, 600 MHz) spectrum of the compound **4s**.

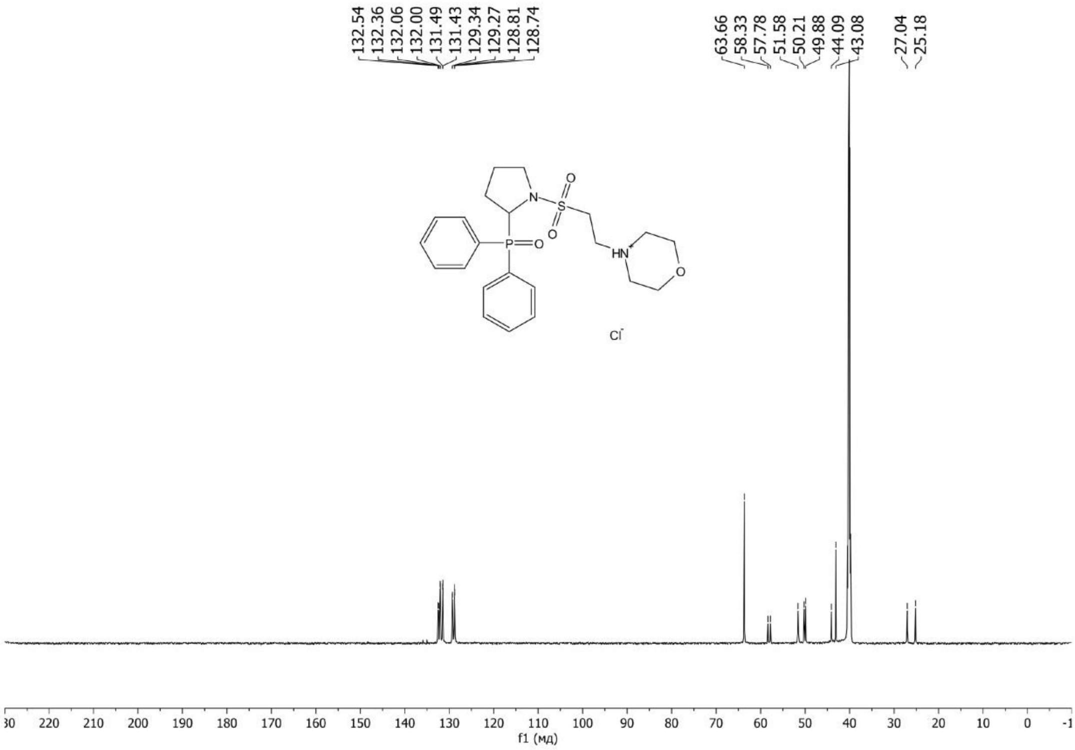

Figure S50. $^{13}C\{^{1}H\}$ NMR (DMSO-d_6, 150 MHz) spectrum of the compound **4s**.

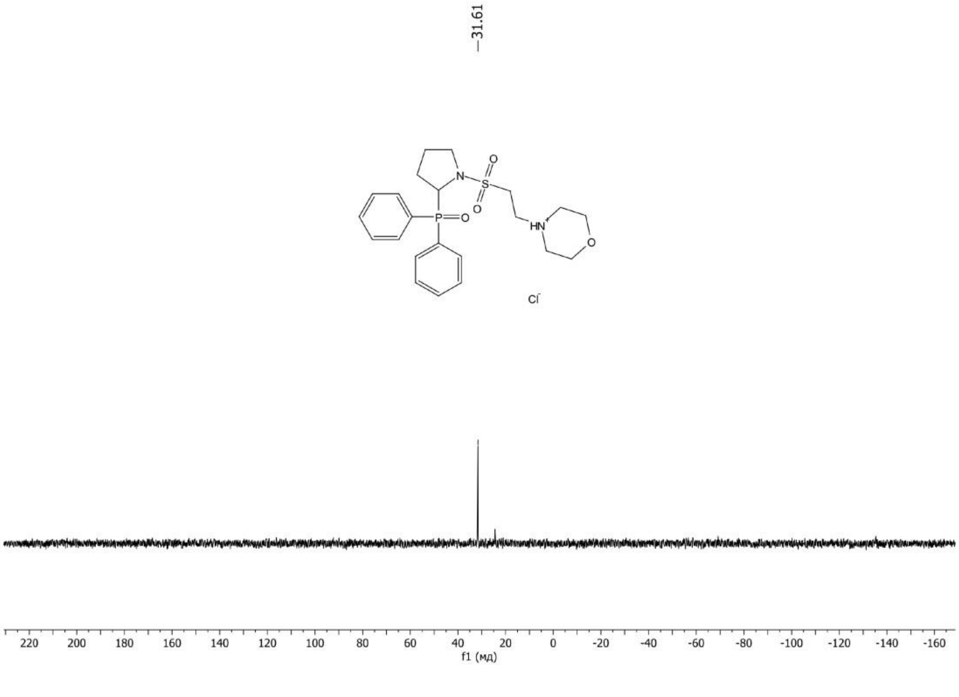

Figure S51. $^{31}P\{^{1}H\}$ NMR spectrum (DMSO-d_6, 161.9 MHz) of the compound **4s**.

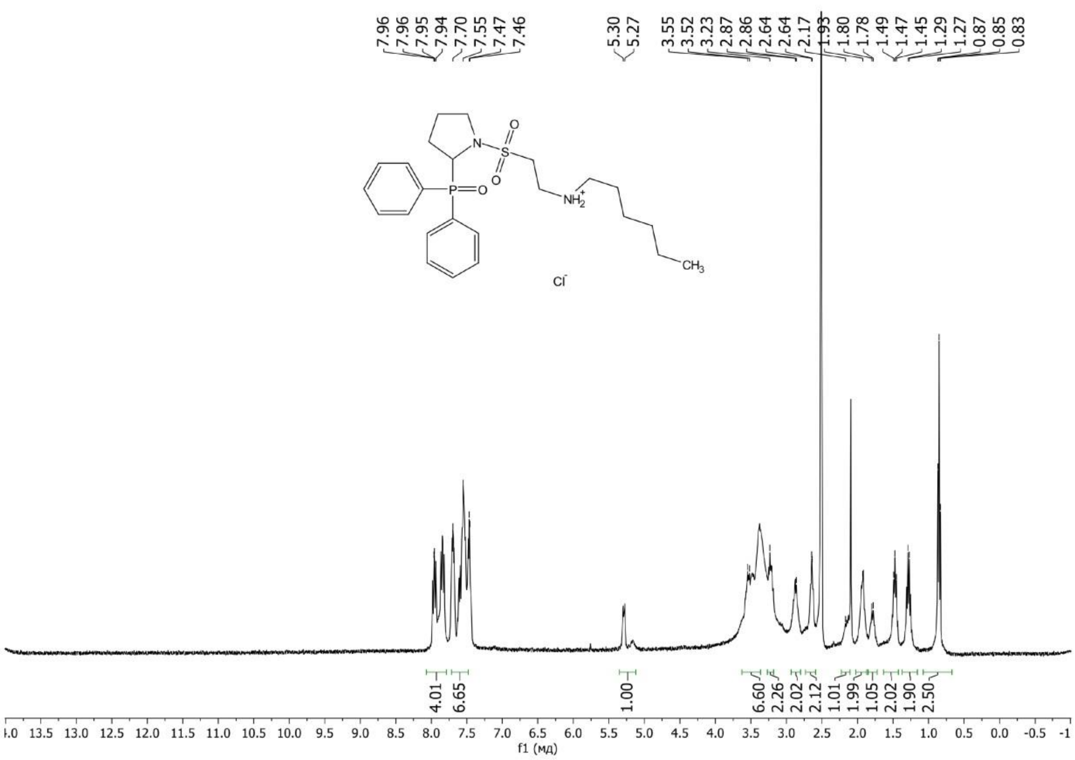

Figure S52. ^{1}H NMR (DMSO-d_6, 600 MHz) spectrum of the compound **4t**.

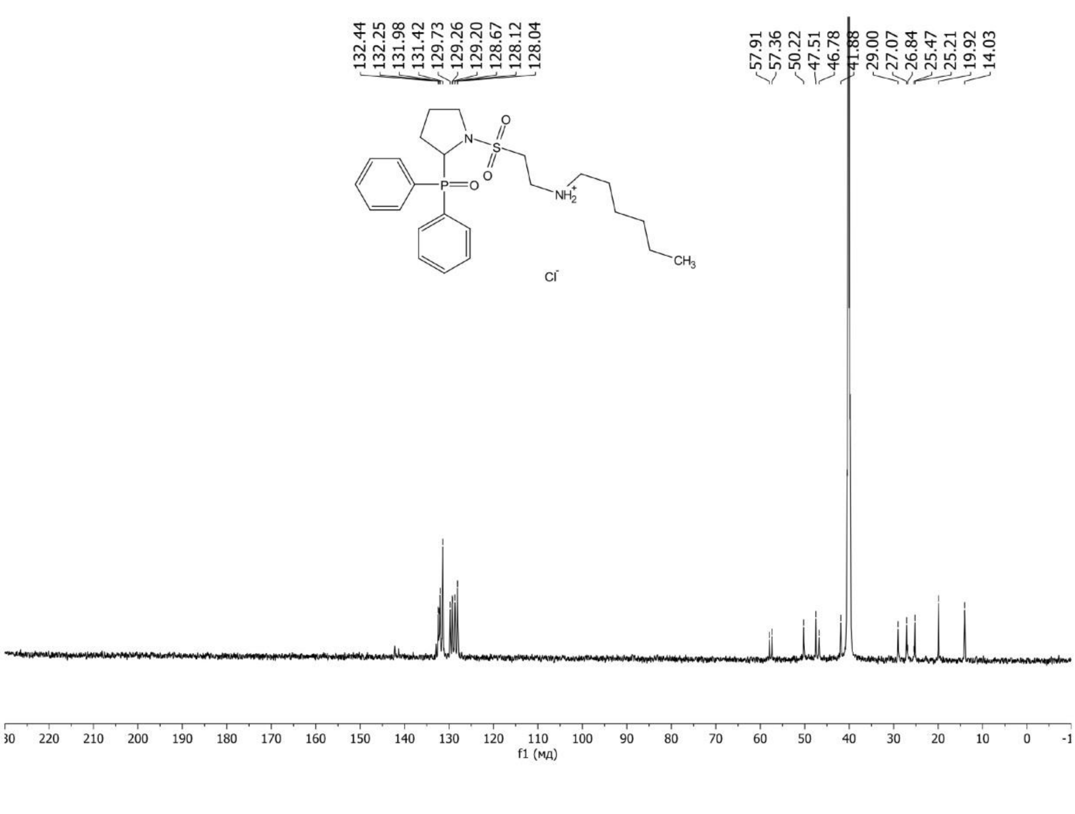

Figure S53. $^{13}C\{^1H\}$ NMR (DMSO-d_6, 150 MHz) spectrum of the compound **4t**.

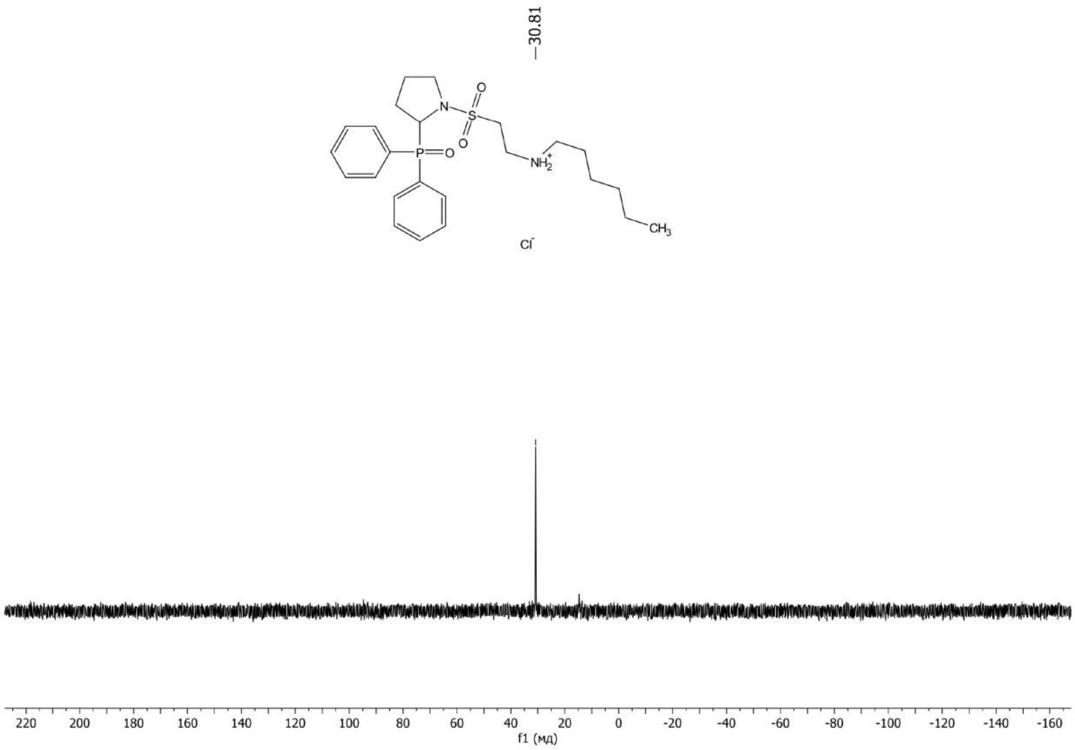

Figure S54. $^{31}P\{^{1}H\}$ NMR spectrum (DMSO-d_6, 161.9 MHz) of the compound **4t**.

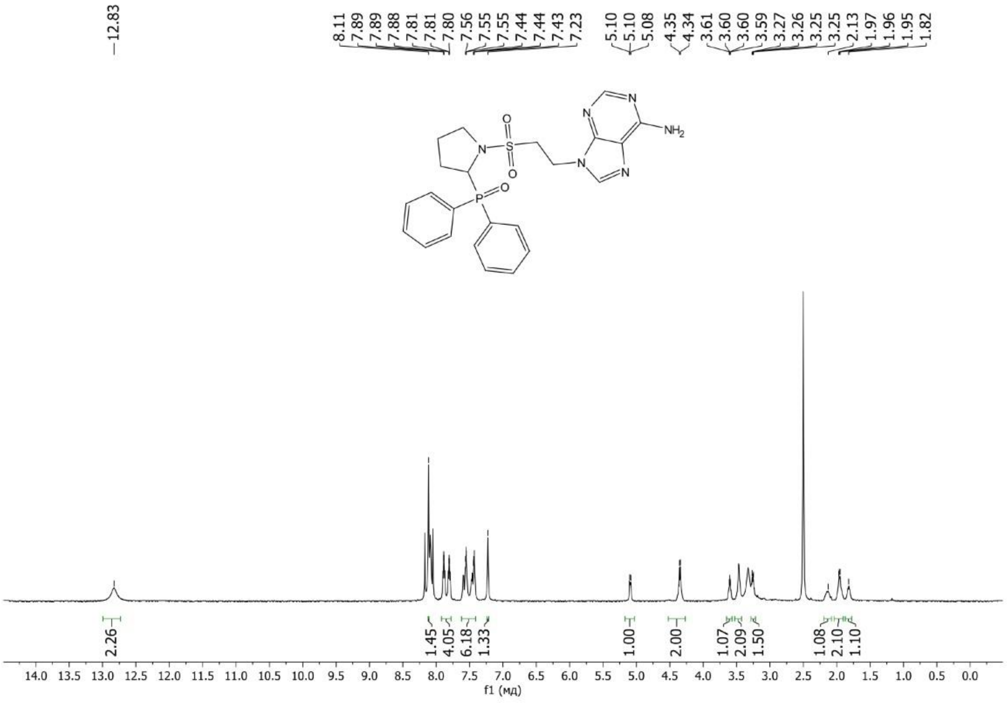

Figure S55. ^{1}H NMR (DMSO-d_6, 600 MHz) spectrum of the compound **4u**.

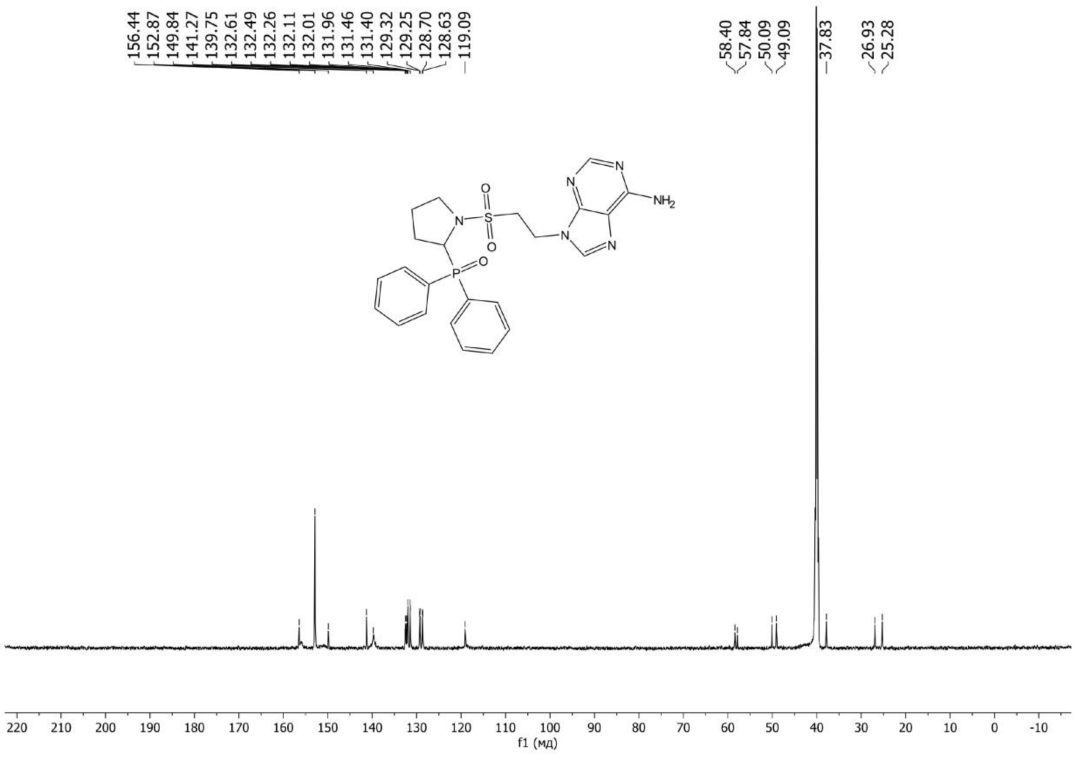

Figure S56. $^{13}C\{^{1}H\}$ NMR (DMSO-d_6, 150 MHz) spectrum of the compound **4u**.

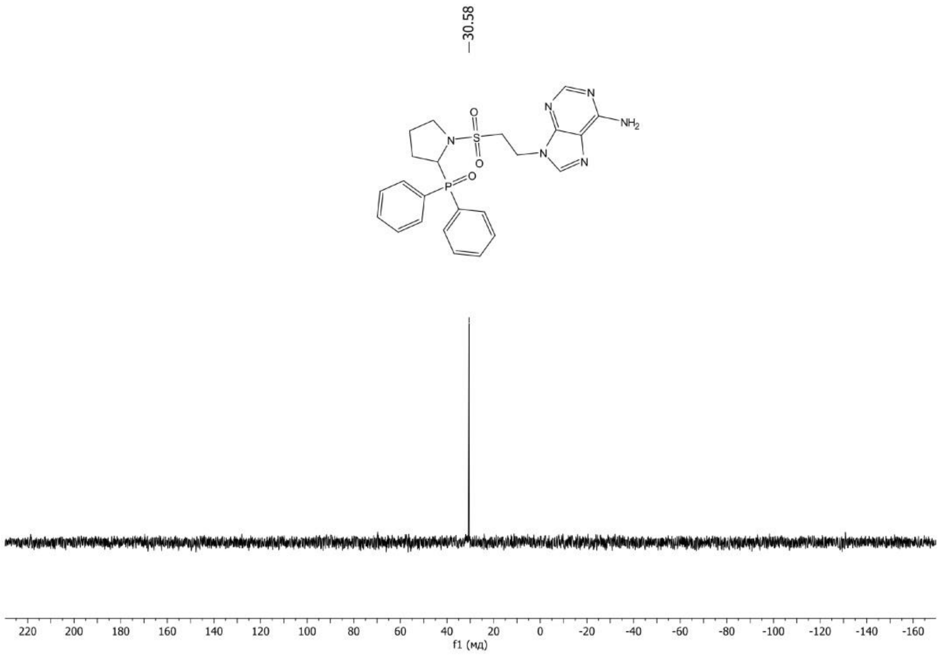

Figure S57. $^{31}P\{^{1}H\}$ NMR spectrum (DMSO-d_6, 161.9 MHz) of the compound **4u**.

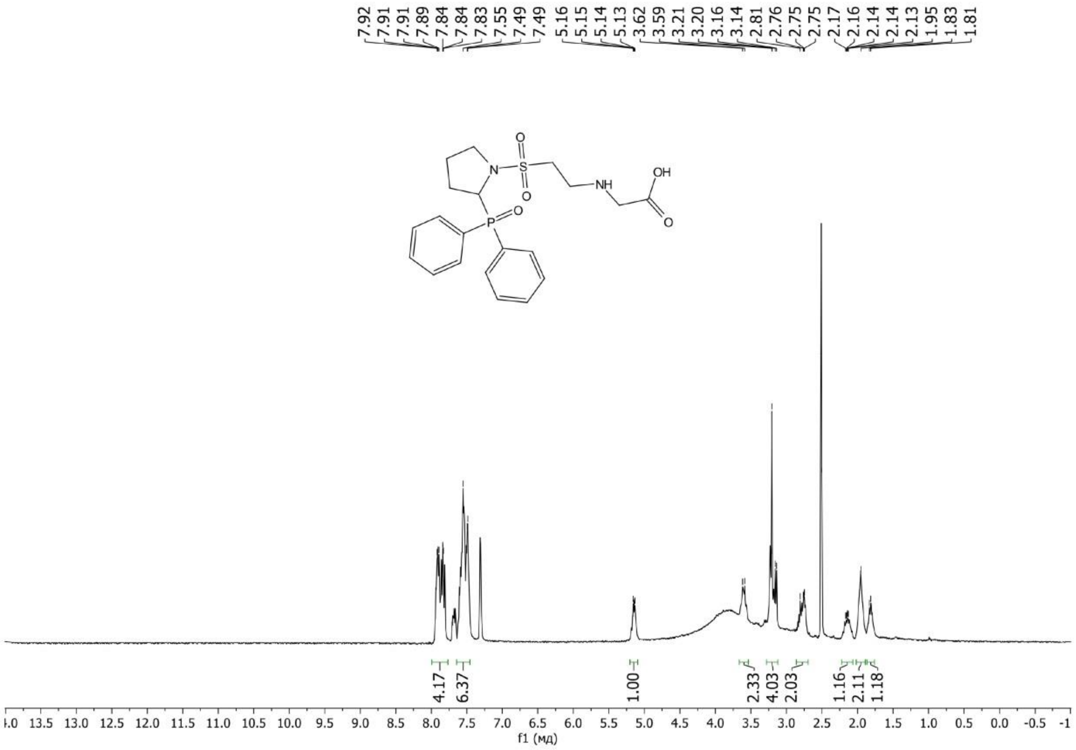

Figure S58. ^{1}H NMR (DMSO-d_6, 600 MHz) spectrum of the compound **4v**.

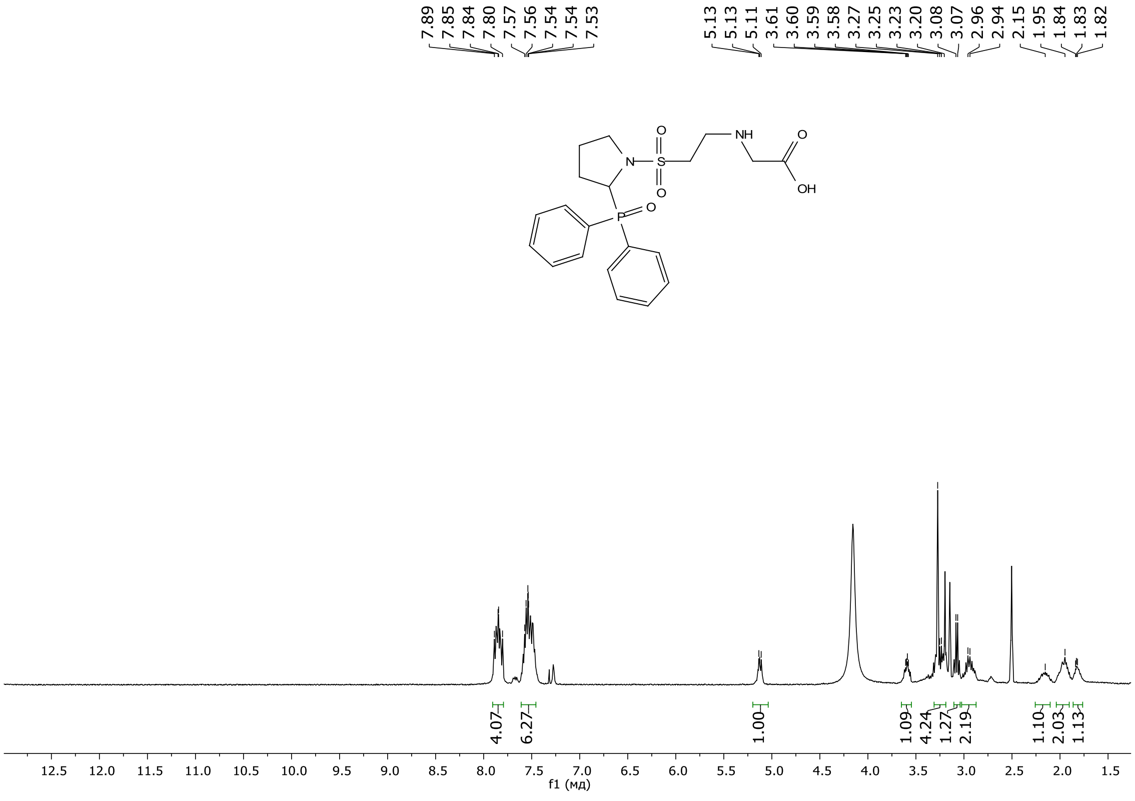

Figure S59. ^{1}H NMR (CD_3OD, 600 MHz) spectrum of the compound **4v**.

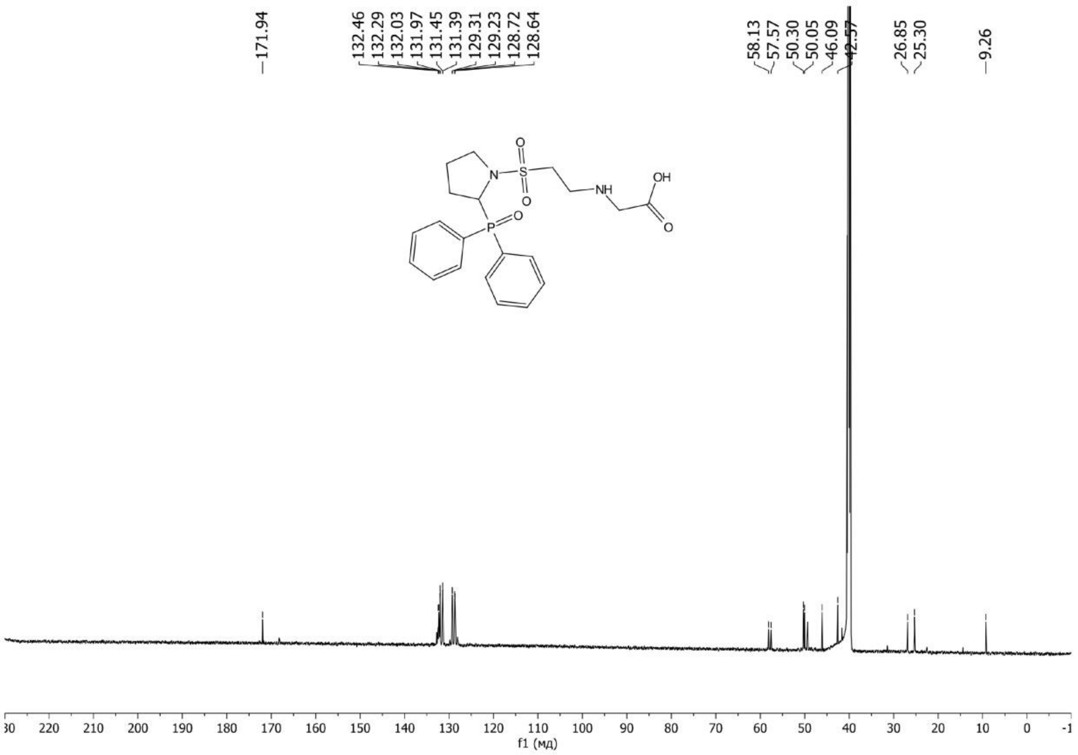

Figure S60. $^{13}C\{^{1}H\}$ NMR (DMSO-d_6, 150 MHz) spectrum of the compound **4v**.

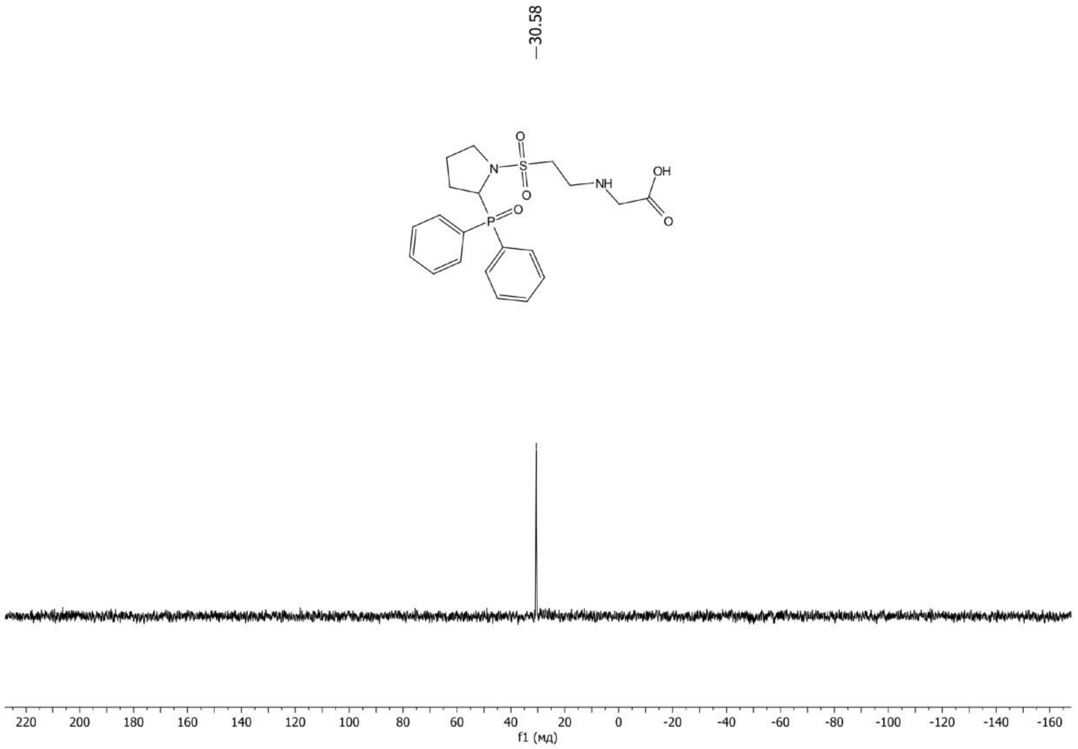

Figure S61. $^{31}P\{^{1}H\}$ NMR spectrum (DMSO-d_6, 161.9 MHz) of the compound **4v**.

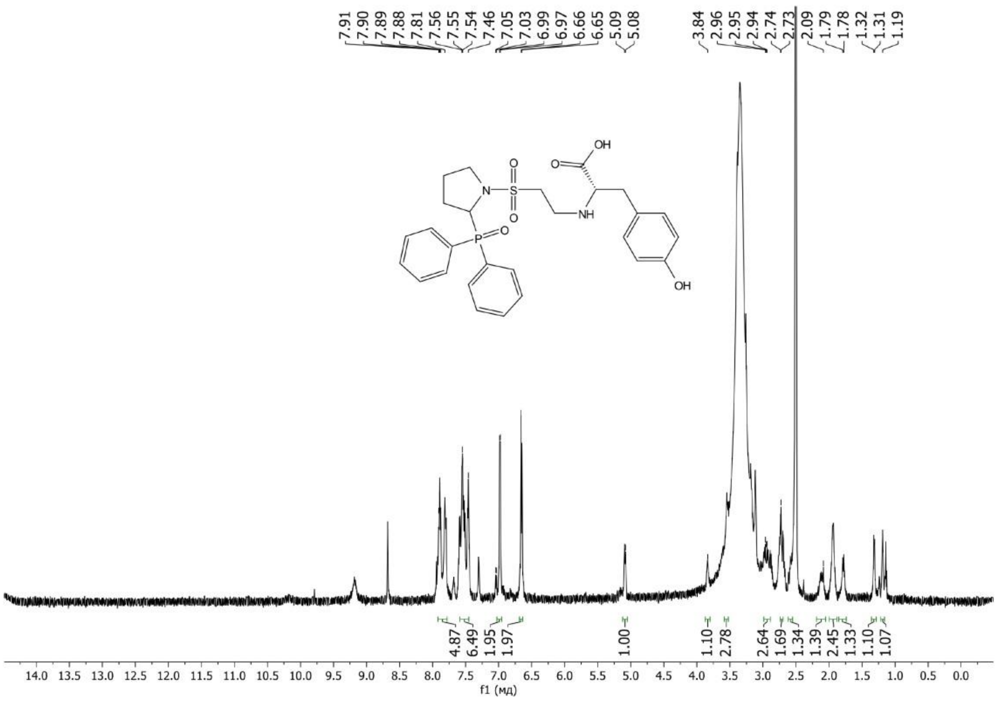

Figure S62. ^{1}H NMR (DMSO-d_6, 600 MHz) spectrum of the compound **4w**.

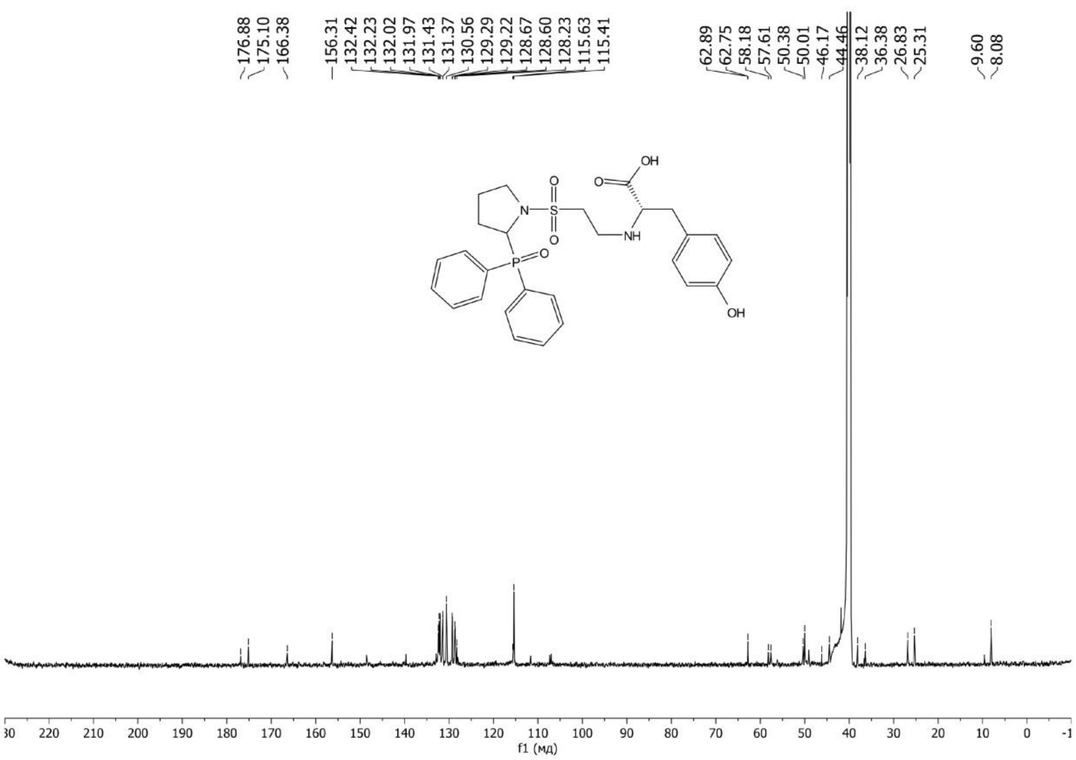

Figure S63. ^{13}C{^{1}H} NMR (DMSO-d_6, 150 MHz) spectrum of the compound **4w**.

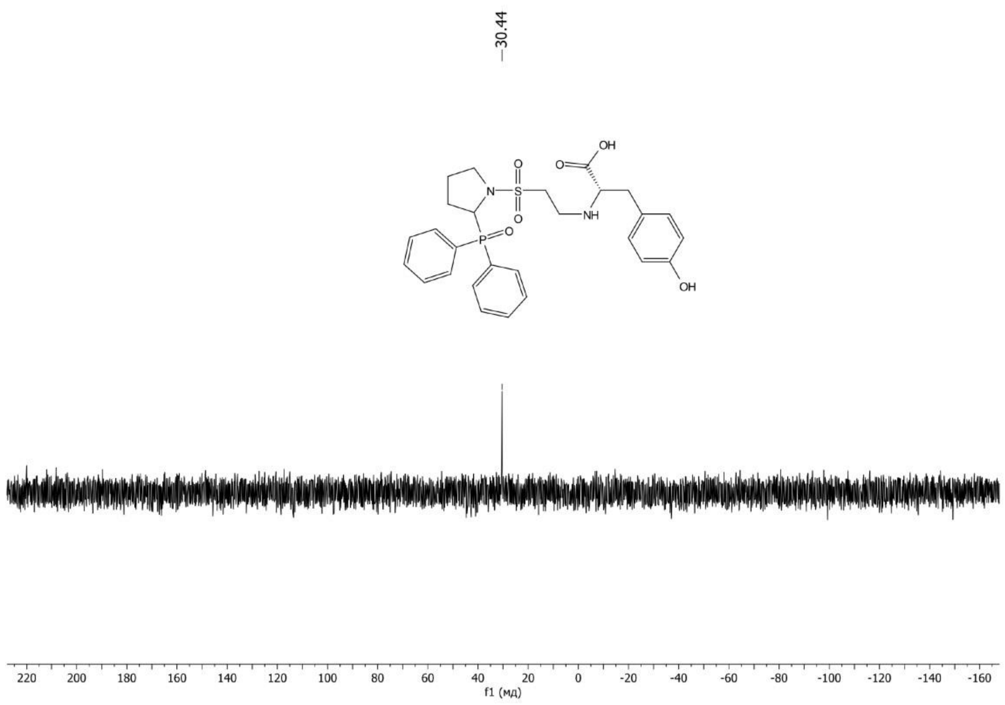

Figure S64. $^{31}P\{^{1}H\}$ NMR spectrum (DMSO-d_6, 161.9 MHz) of the compound **4w**.

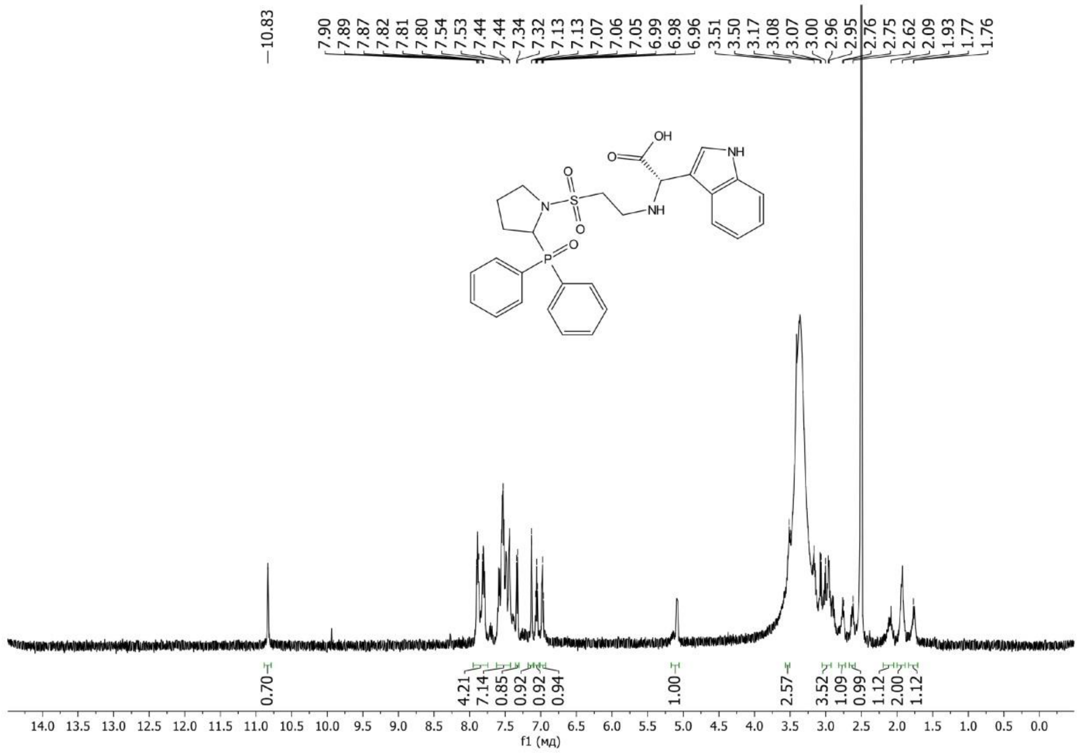

Figure S65. ^{1}H NMR (DMSO-d_6, 600 MHz) spectrum of the compound **4x**.

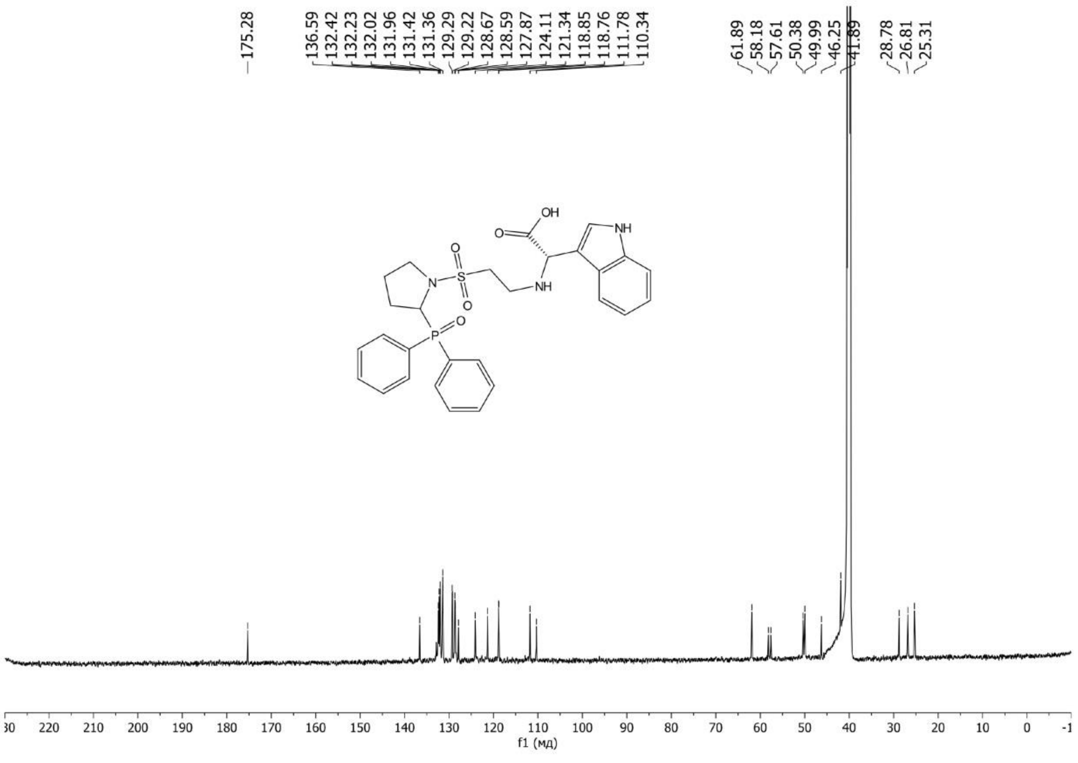

Figure S66. $^{13}C\{^1H\}$ NMR (DMSO-d_6, 150 MHz) spectrum of the compound **4x**.

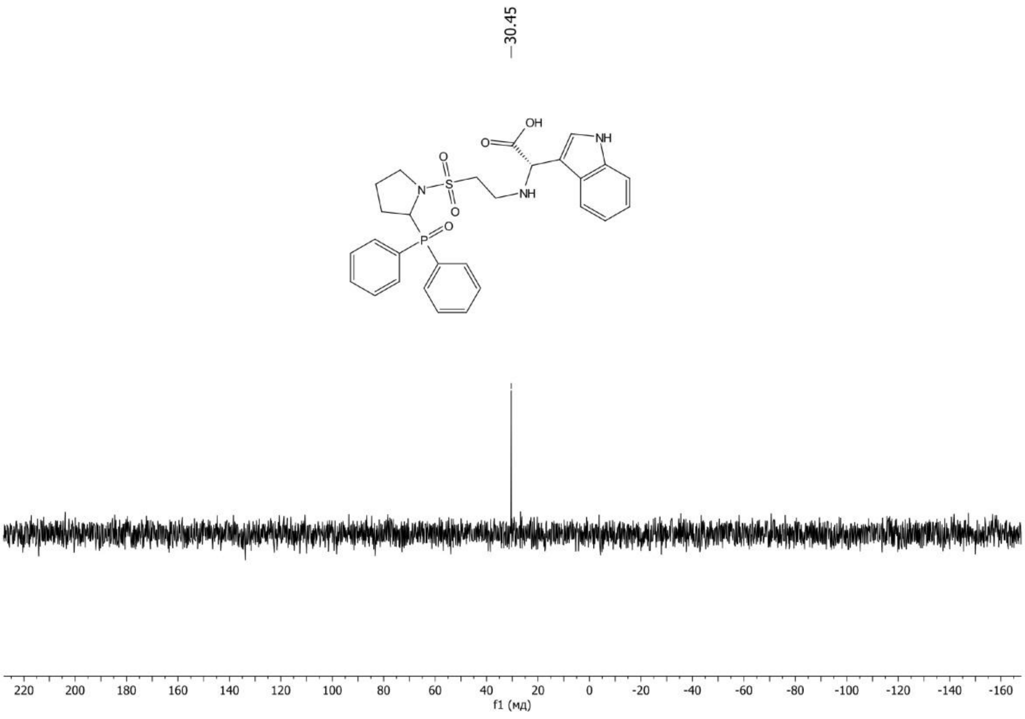

Figure S67. $^{31}P\{^1H\}$ NMR spectrum (DMSO-d_6, 161.9 MHz) of the compound **4x**.

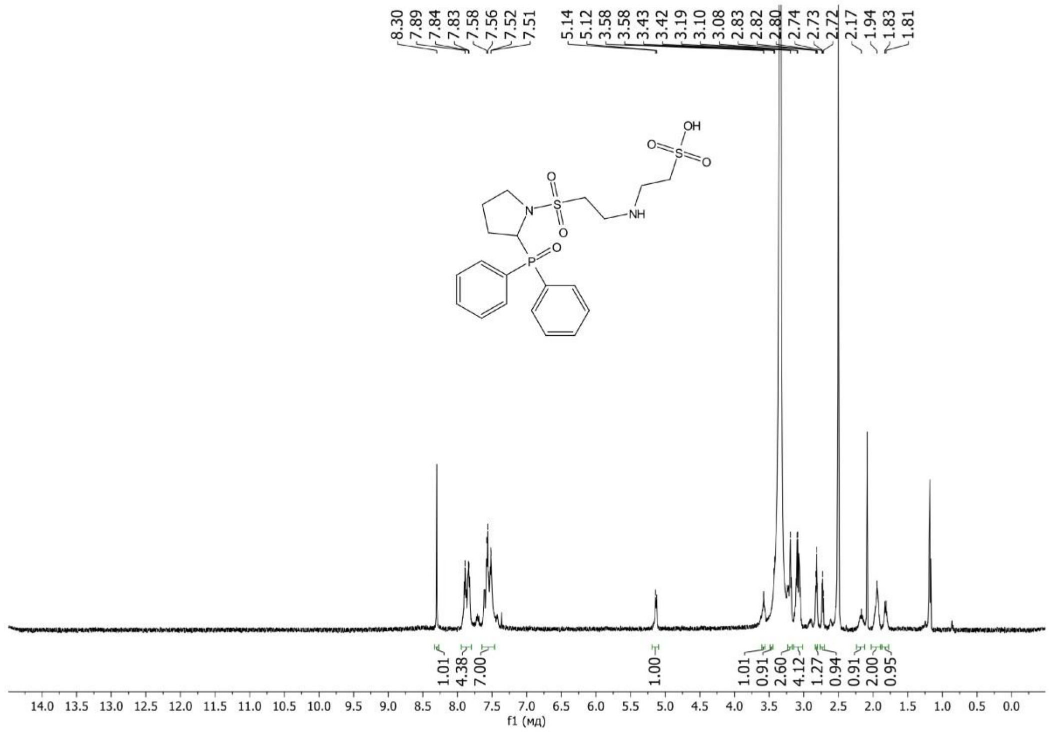

Figure S68. ^{1}H NMR (DMSO-d_6, 600 MHz) spectrum of the compound **4y**.

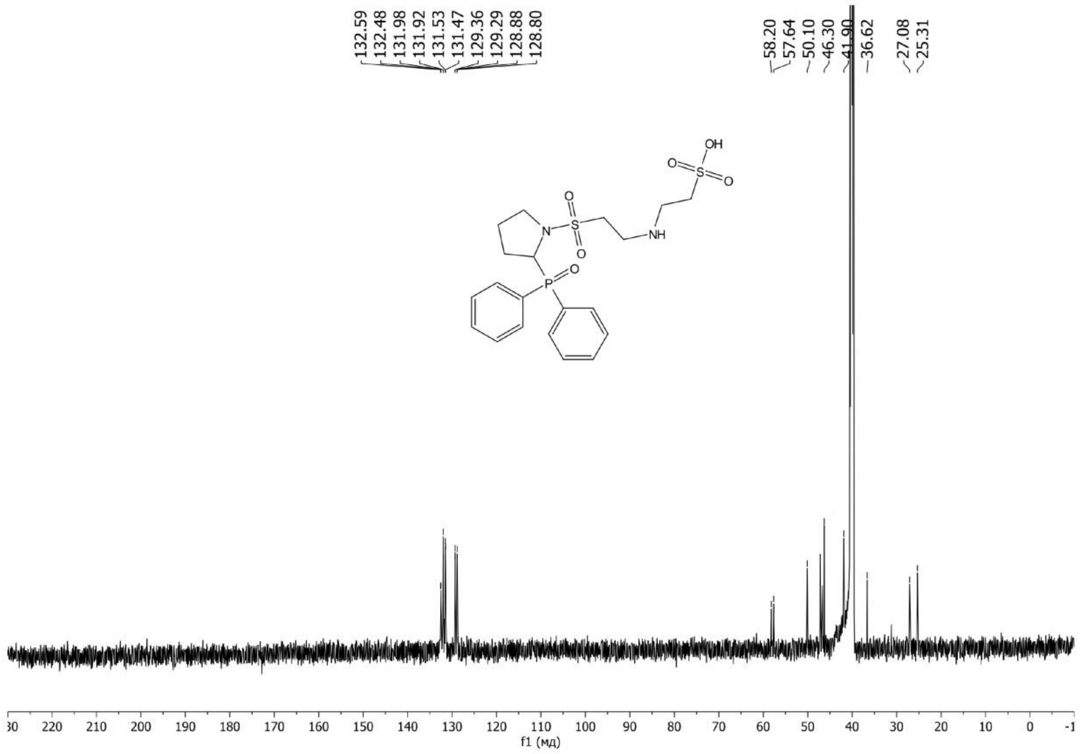

Figure S69. ^{13}C{^{1}H} NMR (DMSO-d_6, 150 MHz) spectrum of the compound **4y**.

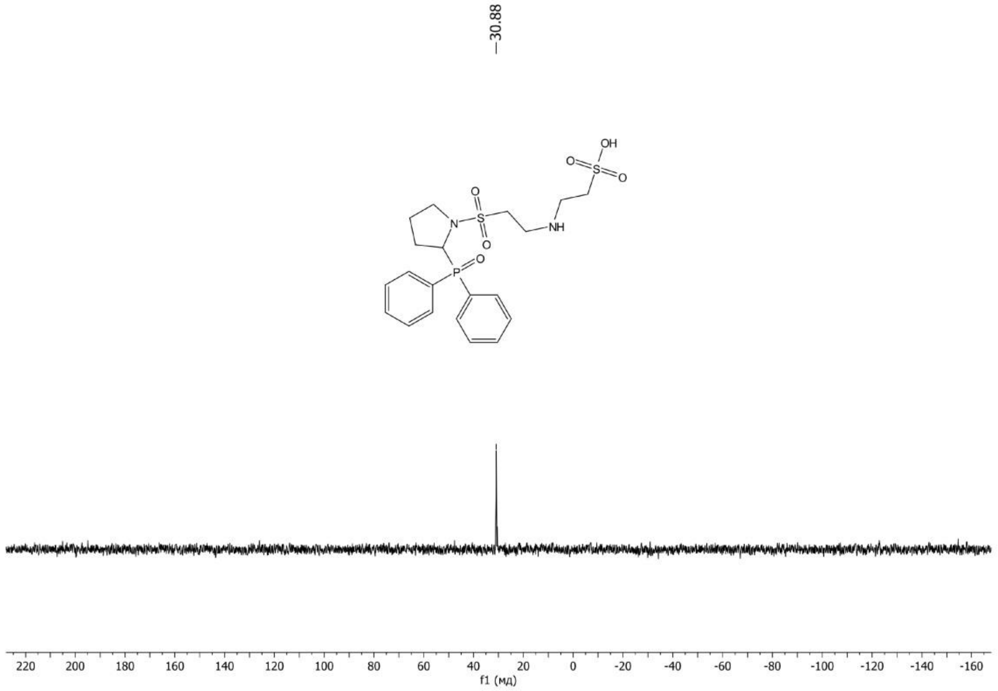

Figure S70. $^{31}P\{^{1}H\}$ NMR spectrum (DMSO-d_6, 161.9 MHz) of the compound **4y**.

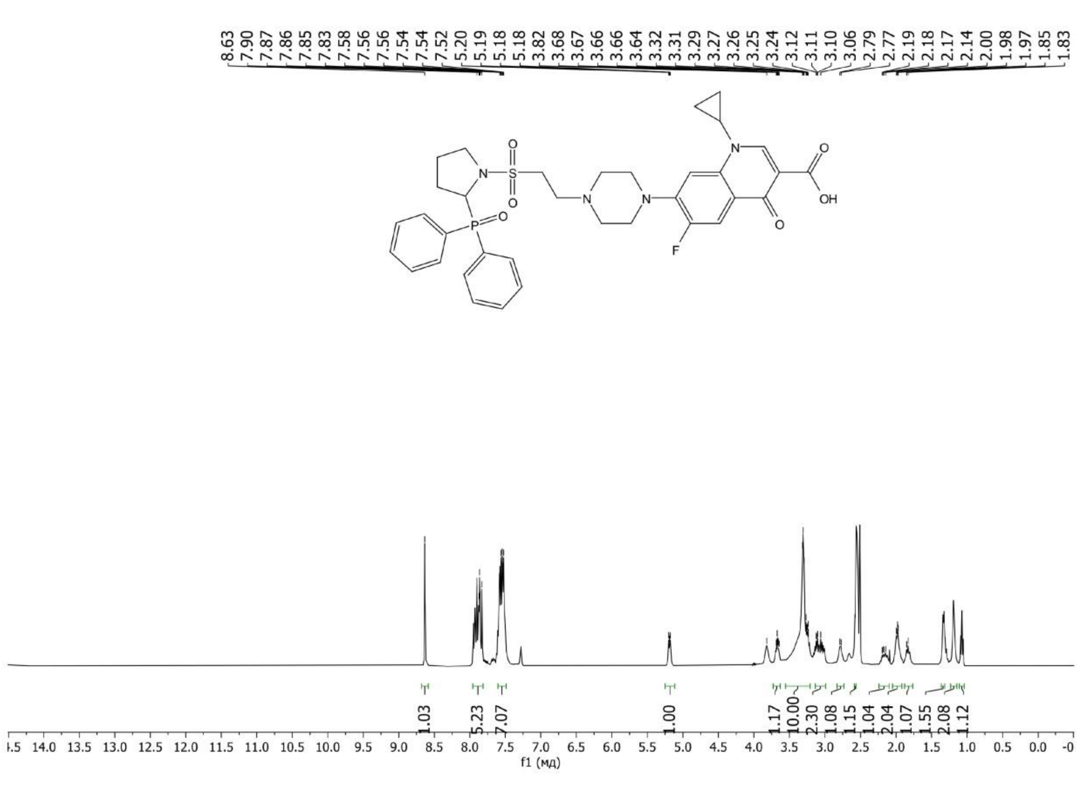

Figure S71. $^{13}C\{^{1}H\}$ NMR (DMSO-d_6, 150 MHz) spectrum of the compound **4z**.

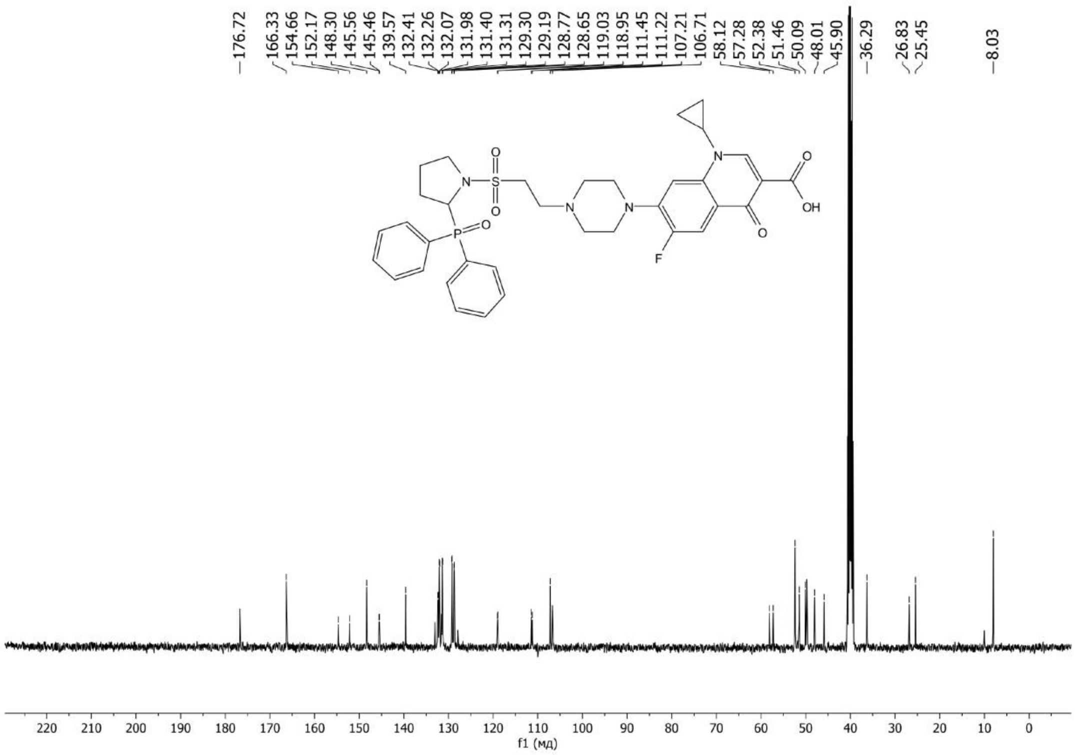

Figure S72. $^{13}C\{^1H\}$ NMR (DMSO-d_6, 150 MHz) spectrum of the compound **4z**.

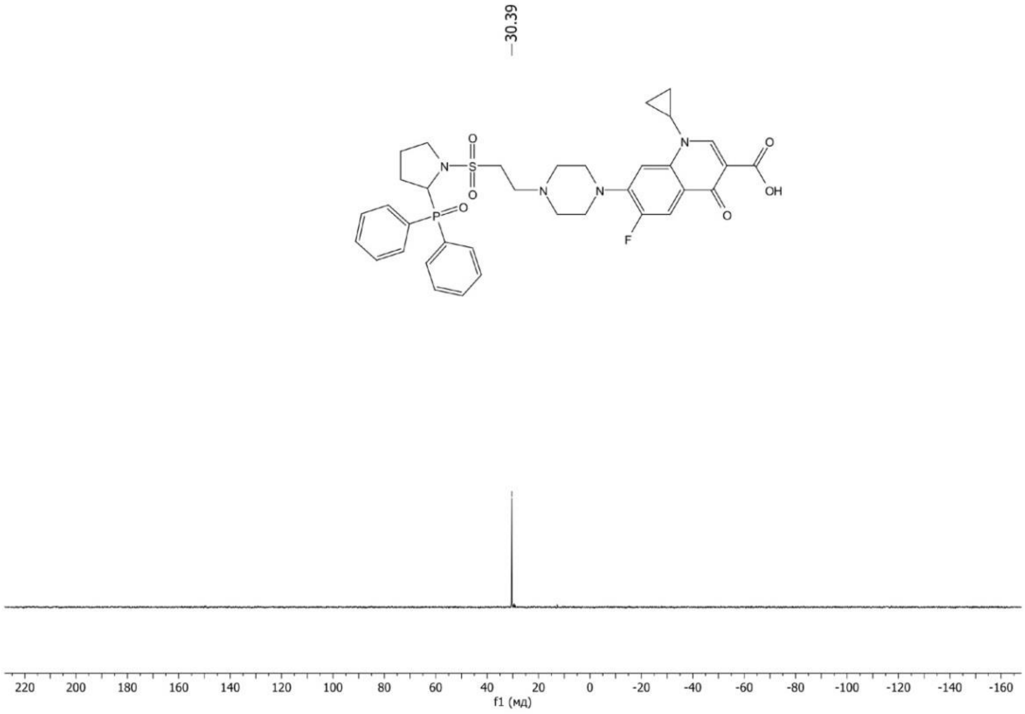

Figure S73. $^{31}P\{^1H\}$ NMR spectrum (DMSO-d_6, 161.9 MHz) of the compound **4z**.
